# Epithelial Yap/Taz are required for functional alveolar regeneration following acute lung injury

**DOI:** 10.1172/jci.insight.173374

**Published:** 2023-09-07

**Authors:** Gianluca T. DiGiovanni, Wei Han, Taylor P. Sherrill, Chase J. Taylor, David S. Nichols, Natalie M. Geis, Ujjal K. Singha, Carla L. Calvi, A. Scott McCall, Molly M. Dixon, Yang Liu, Ji-Hoon Jang, Sergey S. Gutor, Vasiliy V. Polosukhin, Timothy S. Blackwell, Jonathan A. Kropski, Jason J. Gokey

**Affiliations:** 1 Division of Allergy, Pulmonary and Critical Care Medicine, Department of Medicine, Vanderbilt University Medical Center, Nashville, Tennessee, USA.; 2 Department of Cell and Developmental Biology, Vanderbilt University, Nashville, Tennessee, USA.; 3 Department of Veterans Affairs Medical Center, Nashville, Tennessee, USA.

**Keywords:** Pulmonology, Fibrosis, Mouse stem cells

## Abstract

A hallmark of idiopathic pulmonary fibrosis (IPF) and other interstitial lung diseases is dysregulated repair of the alveolar epithelium. The Hippo pathway effector transcription factors YAP and TAZ are implicated as essential for type 1 and type 2 alveolar epithelial cell (AT1 and AT2) differentiation in the developing lung, yet aberrant activation of YAP/TAZ is a prominent feature of the dysregulated alveolar epithelium in IPF. In these studies, we sought to define the functional role of YAP/TAZ activity during alveolar regeneration. We demonstrated that Yap and Taz were normally activated in AT2 cells shortly after injury, and deletion of Yap/Taz in AT2 cells led to pathologic alveolar remodeling, failure of AT2-to-AT1 cell differentiation, increased collagen deposition, exaggerated neutrophilic inflammation, and increased mortality following injury induced by a single dose of bleomycin. Loss of Yap/Taz activity prior to an LPS injury prevented AT1 cell regeneration, led to intraalveolar collagen deposition, and resulted in persistent innate inflammation. These findings establish that AT2 cell Yap/Taz activity is essential for functional alveolar epithelial repair and prevention of fibrotic remodeling.

## Graphical abstract

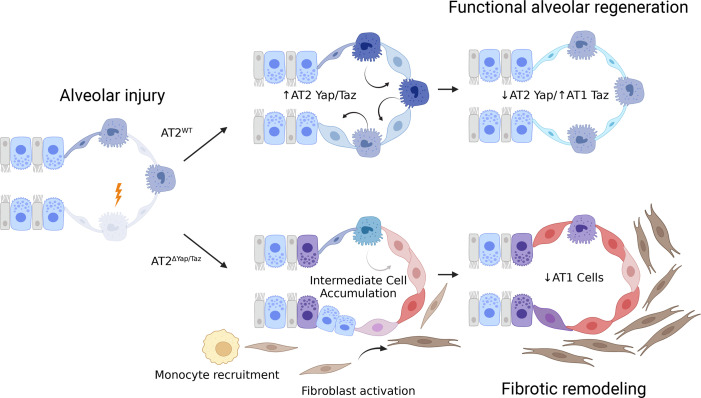

## Introduction

Since the lung is subjected to a large number of injurious agents throughout life, including viral infection, tobacco smoke, and environmental particulates, the capacity for functional repair is essential for maintenance of normal gas exchange. The alveolus, which is responsible for gas exchange, is composed of two primary types of alveolar epithelial cell. Alveolar type 1 (AT1) cells are large, thin, irregularly shaped cells that normally are located in close proximity to alveolar capillaries and facilitate gas exchange in the lung. The AT2 cell secretes pulmonary surfactant and functions as a facultative progenitor cell for AT1 cells, thus supporting both alveolar structure and function (1, 2). Injury to AT1 and AT2 cells requires proliferation and differentiation of progenitor cells to reestablish the epithelial barrier and regenerate functional alveoli. The mechanisms directing this initial proliferative response, followed by differentiation into functional AT1 cells, remain incompletely understood. Recent work from our group and others has implicated the Hippo-Yap/Taz signaling pathway as a regulator of alveolar epithelial development and repair (3–5), and dysregulation of this pathway is a prominent feature of the distal lung epithelium in idiopathic pulmonary fibrosis (6).

The Hippo-Yap/Taz core pathway consists of components mammalian serine threonine kinases *MST1/2* (referred to as serine threonine kinases *Stk4/3* in the mouse) that phosphorylate large tumor suppressor kinases (LATS1/2) that then phosphorylates transcriptional cofactors Yap and Taz (*Wwtr1*). In the presence of this phosphorylation cascade, Yap and Taz are sequestered in the cytoplasm, where they can undergo 14-3-3 ubiquitination and degradation. In the absence of the Hippo phosphorylation cascade, Yap and Taz shuttle to the nucleus, where they interact with binding partners TEAD1–4 (7, 8), as well as Runx2 and Smad2/3, to activate transcription for target genes (9, 10).

In the airway epithelium, there is evidence that temporally regulated Yap activity is required to maintain homeostasis. Sustained activation of Yap leads to basal cell metaplasia, whereas Yap deletion accelerates terminal differentiation of basal cells and impairs basal cell progenitor function, indicating a tight regulation of Yap activity during airway development and repair (11–14). During alveologenesis, ectopic Yap/Taz activation leads to increased AT2 cell proliferation and an increased number of AT1 cells (as defined by Hopx^+^ and Ager^+^ expression), while Yap deletion results in decreased proliferating AT2 cells and a decreased number of AT1 cells (4, 5). We and others have shown that Yap deletion in AT2 cells increases “mature” AT2 cell marker expression, including *Sftpc* and *Abca3* (4, 15, 16). These findings collectively provide evidence that Yap/Taz activity regulates alveolar epithelial cell fate specification during postnatal lung development. However, there has been limited investigation of the Hippo-Yap/Taz pathway during alveolar repair in the adult lung (3).

Herein, we sought to understand the dynamics of Yap/Taz activity during normal alveolar repair and to establish whether Yap/Taz activity is required for functional alveolar regeneration following injury. Using a genetic model of AT2 cell–specific deletion of Yap and Taz, mouse lungs were injured with i.t. delivery of bleomycin or LPS. We found that Yap/Taz deletion leads to persistent alveolar inflammation and results in exaggerated fibroblast activation and lung fibrosis in both bleomycin and LPS injury models. These findings demonstrate that AT2 cell–specific Yap/Taz activation is essential for initiation of alveolar repair and that, without Yap/Taz activation, there is a failure to regenerate AT1 cells necessary for functional alveoli.

## Results

### Alveolar epithelial Yap/Taz activity exhibits cell type–specific dynamic changes during lung injury and repair.

**Figure 1:**
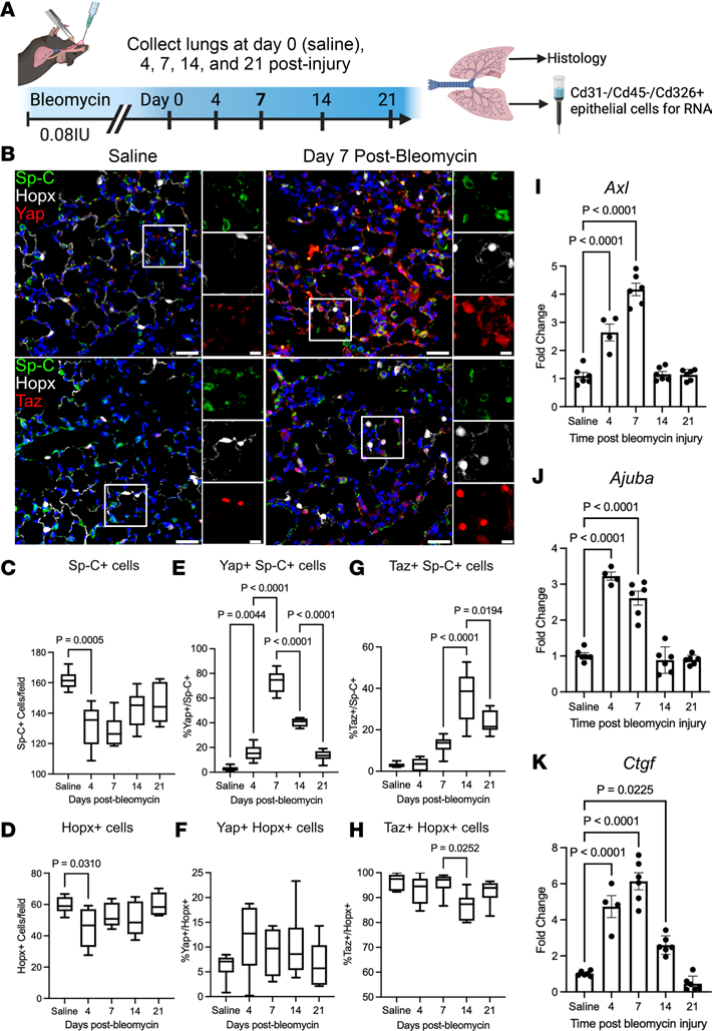
YAP/TAZ are dynamically regulated during acute bleomycin lung injury. (**A**) Schematic of injury model and time points when lungs were assessed. (**B**) Immunofluorescence analysis of Yap or Taz (red) in Sp-C^+^ (green) AT2 and Hopx^+^ (white) AT1 cells. (**C** and **D**) Quantification of Sp-C^+^ AT2 and Hopx^+^ AT1 cells at respective injury repair time points. (**E** and **F**) Quantification of Yap^+^ nuclei in Sp-C^+^ AT2 and Hopx^+^ AT1 cells. (**G** and **H**) Quantification of Taz^+^Sp-C^+^ AT2 and Hopx^+^ AT1 cells. Scale bars: 50 µm, 10 µm (insets). In box-and-whisker plots, whiskers are minimum and maximum, and data represent *n* = 6 mice. (**I**, **J**, and **K**) qPCR analysis of Yap/Taz target genes *Axl* (**I**), *Ajuba* (**J**), and *Ctgf* (**K**) during bleomycin injury repair. *n* = 6 mice for saline, 7, 14, and 21 days after injury and *n* = 4 mice at day 4. Data are shown as mean ± SEM. Statistical analysis was performed using 1-way ANOVA and Tukey’s post hoc comparison.

To understand the dynamics of Yap/Taz activity during alveolar injury and repair, WT C57BL/6J mice were challenged with a single dose of i.t. bleomycin (0.08 IU) and sacrificed 4, 7, 14, and 21 days after bleomycin injury (Figure 1A). In control mice, nuclear Yap or Taz was rarely detected in Sp-C^+^ AT2 cells (Figure 1, B, E, and G), and nuclear Yap was detected in a small minority of Hopx^+^ AT1 cells (5.1% ±1.4%); however, nuclear Taz was present in nearly all (97.9% ± 1.1%) AT1 cells (Figure 1, F and H). After bleomycin-induced injury, quantification of Sp-C^+^ AT2 and Hopx^+^ AT1 cells revealed that both cell types are reduced by day 4 and return to baseline numbers by 21 days after injury (Figure 1, C and D). At 7 days after bleomycin, nuclear Yap and Taz were found in 72.7% ± 2.9% and 12% ± 2.1% of Sp-C^+^ cells, respectively (Figure 1). At 14 days after injury, 40.0% ± 2.2% of Sp-C^+^ AT2 cells had nuclear Yap, which decreased further to 13.3% ± 1.5% by day 21. In comparison, nuclear Taz was identified in 35.2% ± 4.2% of AT2 cells at day 14 and to 23.1% ± 2.8% at 21 days after injury. Yap was present in approximately 10%–13% of Hopx^+^ AT1 cells during repair (days 4, 7, and 14) and returned to 6.7% ± 1.6% by day 21. In contrast, Taz was readily detected in > 93% of Hopx^+^ AT1 cells throughout homeostasis and repair, with the exception of 14 days after injury, when Taz was detected in 86.2% ± 1.9% of Hopx^+^ AT1 cells. Quantitative PCR (qPCR) analysis of Yap/Taz targets in sorted Cd326^+^ epithelial cells identified a similar trend in RNA levels of *Ctgf*, *Axl*, and *Ajuba*, as targets are highest expressed by day 7 after injury and return to baseline levels by day 21 (Figure 1, I–K). These findings indicate that alveolar injury is followed by an asynchronous activation of Yap/Taz in regenerating alveolar epithelial cells, peaking at days 7 and 14, followed by downregulation of Yap and stabilization of Taz in AT1 cells as the lung returns to homeostasis.

### Yap is required for initiation of alveolar repair following bleomycin injury.

**Figure 2:**
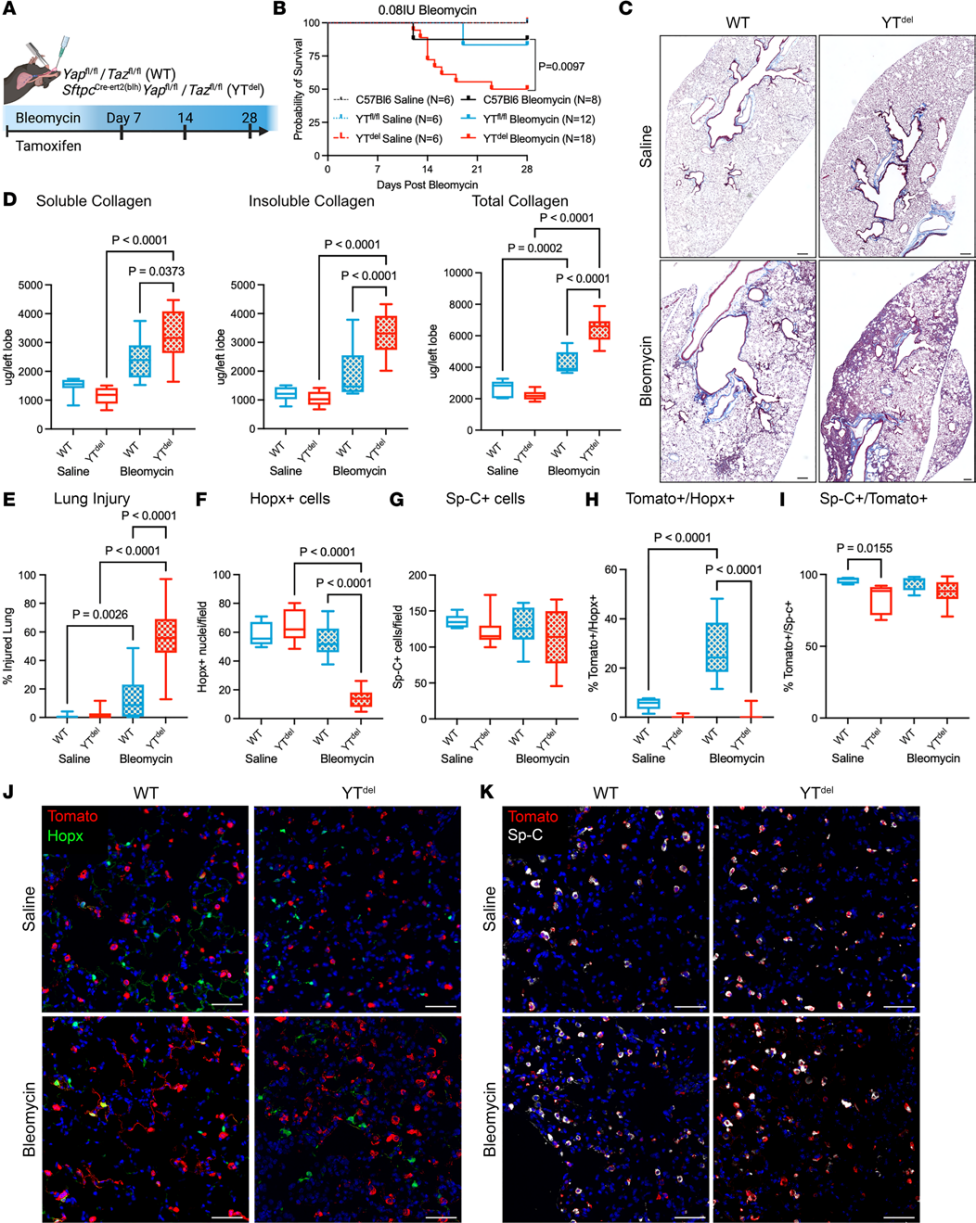
Deletion of YAP/TAZ leads to failed alveolar repair following single-dose bleomycin induced lung injury. (**A**) Schematic of injury model in which mice were treated with tamoxifen the same day as bleomycin-induced lung injury. (**B**) Survival curve of WT and YT^del^ bleomycin- or saline-treated mice out to 28 days after injury. Statistics determined using Mantel-Cox test. (**C**) Masson’s trichrome staining of tissue sections from WT and YT^del^ mice at day 28 after bleomycin or PBS. (**D**) Soluble, insoluble, and total collagen quantification from WT and YT^del^ at day 28 after saline or bleomycin. (**E**) Quantification of total injured lung area in respective groups at day 28 after saline/bleomycin. *n* = 7 WT saline, *n* = 9 YT^del^ saline, *n* = 9 WT bleomycin, and *n* = 12 YT^del^ bleomycin mice. (**F** and **G**) Quantification of total Hopx^+^ cells or Sp-C^+^ cells per 20***×*** field of view in each group. *n* = 10 WT saline-, *n* = 9 YT^del^ saline-, *n* = 12 WT bleomycin-, and *n* = 18 YT^del^ bleomycin-treated mice. (**H** and **I**) Quantification of Sp-C^tomato^ lineage–labeled Hopx^+^ or Sp-C^+^ AT2 cells 28 days after bleomycin. (**J**) Immunofluorescence analysis of Sp-C^tomato^ lineage labeled cells (red) and Hopx^+^ (green) AT1 cells at 28 days after injury. (**K**) Immunofluorescence analysis of Sp-C^tomato^ lineage–labeled cells (red) and Sp-C^+^ (white) AT2 cells at 28 days after injury. *n* = 5 WT saline, *n* = 5 YT^del^ saline, *n* = 10 WT bleomycin, and *n* = 15 YT^del^ bleomycin mice. Scale bars: 200 µm (**C**), 50 µm (**J** and **K**). Statistical analysis in **D**–**I** was performed using 1-way ANOVA and Tukey’s post hoc comparison.

Having observed early activation of Yap/Taz in AT2 cells following injury, we then sought to determine the role of AT2 cell Yap/Taz in regulating alveolar repair. *Yap^fl/fl^ Taz^fl/fl^* mice were crossed with the AT2 cell–specific promoter *SftpcCre^ert2^* (17) to drive tamoxifen-inducible deletion of Yap and Taz (hereafter referred to as YT^del^ mice) in 8- to 10-week-old adult mice 2 weeks prior to bleomycin-induced lung injury (Supplemental Figure 1A; supplemental material available online with this article; https://doi.org/10.1172/jci.insight.173374DS1). YT^del^ mice injured with i.t. bleomycin resulted in high mortality during the first 2 weeks after injury. I.t. delivery of 0.08 IU/mouse resulted in approximately 75% mortality (Supplemental Figure 1, B and C). Therefore, to establish a system in which the epithelial-repair response could be assessed, we treated mice with tamoxifen to delete Yap/Taz at the time of bleomycin injury (Figure 2A), and this resulted in increased survivability compared with the previous model, which had 50% mortality in YT^del^ mice compared with WT Yap^fl/fl^Taz^fl/fl^ and C57BL/6J control mice at 28 days after injury (Figure 2B). Masson’s trichrome staining (Figure 2C) and Sircol collagen analysis revealed that bleomycin-treated YT^del^ mouse lungs had increased soluble and total collagen compared with control mice (Figure 2D). YT^del^ mice had increased injured lung area as assessed by histological analysis of areas with collagen deposition or immune infiltrate (18), 28 days after bleomycin compared with WT sibling mice (Figure 2E). Immunofluorescence analysis showed that YT^del^ mice had reduced Hopx^+^ cells, while numbers of Sp-C^+^ cells remained similar to unchallenged mice 28 days after injury (Figure 2, F and G).

Observing reduced AT1 cell numbers in YT^del^ mice, we next sought to directly determine the ability of YT^del^ AT2 cells to act as progenitor cells to regenerate AT1 and AT2 cell populations. We performed lineage-tracing studies using YT^del^ mice crossed with Rosa26-lox-stop-lox-tDtomato reporter mice, in which a Tomato fluorescent reporter is activated in AT2 cells when mice are treated with tamoxifen (19). While the number of lineage-labeled Sp-C cells (AT2 cells) was similar in bleomycin injured YT^del^ and WT mice, the number of lineage-labeled Hopx^+^ cells (AT1 cells) was reduced from 27.8% ± 3.3% in WT mice to 1.1% ± 0.4% in YT^del^ mice at 28 days after injury (Figure 2, H–K). Next, to test whether the effects of YT deletion on AT2 progenitor function were mediated through an autonomous mechanism, we isolated Cd45^–^/Cd31^–^/Cd326^+^ epithelial cells from lineage-labeled naive WT and YT^del^ mice and established feeder-free organoids using recently described methodlology (20) (Supplemental Figure 2A). By day 14, quantification of Tomato^+^ organoids revealed a dramatic reduction in the number (57.8 ± 3.9 compared with 5.1 ± 0.70) and size (77.8 ± 1.4 compared with 45.8 ± 1.9 mm) of organoids generated from YT^del^ AT2 cells compared with those generated with WT AT2 cells (Supplemental Figure 2, B and C). Together, these findings demonstrate that loss of Yap and Taz in AT2 cells prevents AT2-to-AT1 differentiation and impairs AT2 progenitor function.

### AT2 cell–specific deletion of Yap/Taz leads to increased intermediate alveolar epithelial cell numbers, activation of fibroblasts, and increased immune cell populations.

**Figure 3:**
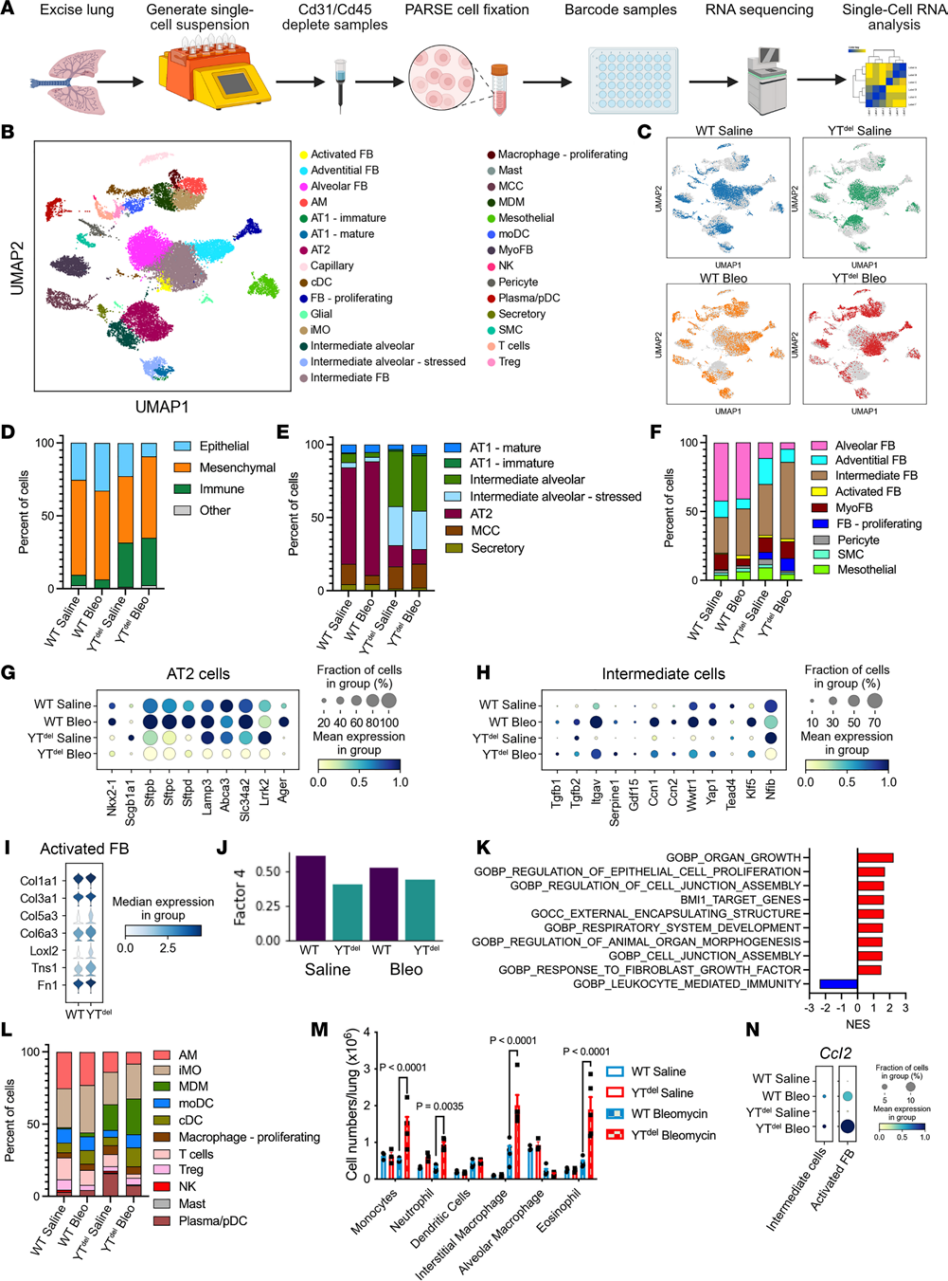
YAP/TAZ deletion prior to bleomycin injury leads to altered epithelial cell populations, activated fibroblast, and disrupted immune response. (**A**) Schematic of experimental strategy to generate single-cell RNA-Seq analysis from mouse lungs. (**B** and **C**) UMAP embedding demonstrating clustering of the 29 cell types identified in the mouse lungs (**B**) and depicting cells recovered from respective genotype and treatment groups (**C**). (**D**–**F**) Relative proportions of each cell types/states within total cell populations (**D**), epithelial cell populations (**E**), and fibroblast cell populations between each treatment group (**F**). (**G** and **H**) Dot plot showing expression levels of AT2 cell lineage markers (**G**) and intermediate epithelial cell marker expression (**H**) in each treatment group. (**I**) Violin plot showing relative expression of activated fibroblast markers in WT or YT^del^ bleomycin-treated mice. (**J**) Output from LIANA demonstrating loss of Factor 4 ligand-receptor signaling in YT^del^ mice. (**K**) Gene ontology–associated programs identified as misregulated by LIANA. (**L**) Relative proportions of immune cell populations from each treatment group. (**M**) Flow cytometry analysis of immune cell subpopulations present in mice 7 days after bleomycin injury. *n* = 3 WT saline- and YT^del^ saline-, *n* = 5 WT bleomycin- and YT^del^ bleomycin-treated mice. (**N**) Dot plot showing expression of *Ccl2* in intermediate alveolar epithelial cells and activated (including both intermediate and activated from **F**) fibroblast population.

To investigate the mechanisms through which AT2 cell deletion of YT alters epithelial cell transcriptional programs and worsens experimental lung fibrosis, we performed single-cell RNA-Seq of lung single-cell suspensions generated from YT^del^ and WT saline- and bleomycin-treated mouse lungs at 28 days after injury (Figure 3A). Analysis identified 29 cell types (Figure 3B and Supplemental Figure 3), including numerous bleomycin-emergent and/or enriched populations (Figure 3, D–F). Compared with WT mice, there were fewer epithelial cells and increased proportions of fibroblasts and immune cells in YT^del^ mice (Figure 3D). In particular, intermediate fibroblasts (characterized by increased *Col1a1* and *Col3a1* expression and lower levels of *Pdgfra*) and activated fibroblasts (identified by the highest expression of *Col1a1* and *Cthrc1*) (21) comprised the majority of fibroblasts recovered from YT^del^ bleomycin-treated lungs (Figure 3, C and E, and Supplemental Figure 3). In addition to increased numbers of intermediate, activated, and proliferating fibroblasts, combined analysis of these emergent fibroblasts demonstrated increased expression of fibrillar collagens *Col1a1*, *Col3a1*, *Col5a3*, and *Col6a3* as well as *Fn1* in YT^del^ mice compared with controls (Figure 3, F and I). There was a shift toward increased *Gdf15^+^* intermediate alveolar epithelial cells and decreased AT2 cells in the YT^del^ bleomycin-treated populations (Figure 3, E and H). As expected, YT^del^ AT2 cells had reduced Yap/Taz signaling and, after bleomycin, exhibited lower levels of transitional/intermediate/AT1 cell markers, Yap/Taz binding partner *Nfib*, and canonical YT target genes *Ccn1* and *Ccn2* but increased expression of canonical monocyte chemotactic factor *Ccl2* and TGF-β–activating integrin aV (*Itgav*) (Figure 3, D and G). To assess how deletion of Yap/Taz in the AT2 cell population may be leading to activation of the fibroblast, we performed ligand-receptor based cellular communication analysis using Tensor-cell2cell (22) implemented with LIANA (Figure 3, J and K) (23). This approach uses unsupervised tensor decomposition to extract Factors that can reflect context-specific communication. We observed that Factor 4 differed most significantly in YT^del^ mice compared with controls, and pathway enrichment of top ligand-receptor pairs suggested dysregulation of FGF and IGF signaling as well as reduced signaling associated with cell junctions (Figure 3K). Complete output from this analysis is available in the supplemental Gene Set Enrichment Analysis (GSEA) file. YT^del^ bleomycin-injured lungs had increased inflammatory cells overall, with increased monocyte-derived macrophages compared with WT (Figure 3, L and K). With evidence of exaggerated and persistent inflammation 28 days after bleomycin, we performed flow cytometry to quantify immune subpopulations at the time of peak inflammation (7 days after bleomycin) (Supplemental Figure 4). YT^del^ lungs had increased numbers of monocytes, neutrophils, and interstitial macrophages 7 days after bleomycin injury (Figure 3M). Analysis of single-cell gene expression found that *Ccl2* was increased in both the intermediate alveolar epithelial cells and the activated fibroblast population (consisting of both the activated and intermediate subpopulations) (Figure 3N).These findings support the concept that Yap/Taz deletion specifically in AT2 cells leads to abnormal alveolar epithelial cell repair, promotes recruitment of monocyte-derived macrophages, and enhances profibrotic activation and proliferation of resident fibroblasts.

### Deletion of Yap/Taz leads to failed alveolar repair and fibrotic remodeling after LPS injury.

**Figure 4:**
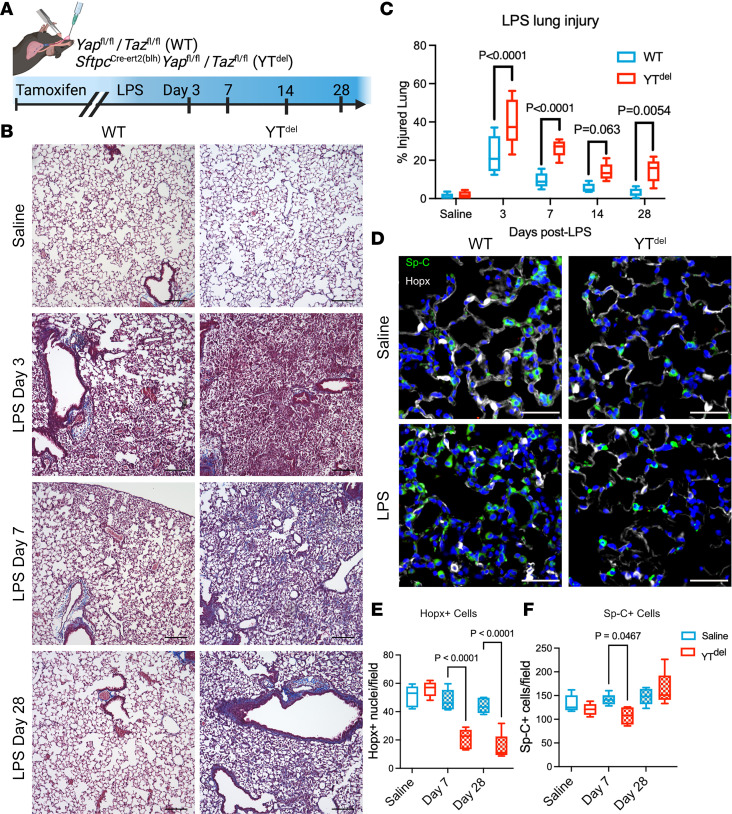
Loss of YAP/TAZ prior to LPS-induced lung injury leads to failed alveolar regeneration and increased collagen deposition. (**A**) Schematic of LPS lung injury model in which mice were treated with tamoxifen 2 weeks prior to LPS lung injury. (**B**) Masson’s trichrome staining of WT and YT^del^ mice treated with saline or 3, 7, or 28 days after LPS injury. *n* = 6 mice per group. (**C**) Quantification of injured lung area in WT and YT^del^ mice 3 to 28 days after LPS. (**D**) Immunofluorescence analysis of Hopx^+^ (white) AT1 cells and Sp-C^+^ (green) AT2 cells 7 days after LPS injury. (**E** and **F**) Quantification of Hopx^+^ and Sp-C^+^ cells in WT or YT^del^ lungs treated with saline or LPS at 7 or 28 days after injury; *n* = 5 mice per group. Statistics were performed using 1-way ANOVA and Tukey’s post hoc comparison. Scale bars: 50 µm (**D**), 200 µm (**B**).

To determine whether AT2 cell Yap/Taz is more generally required for adaptive alveolar repair, we then performed a series of experiments in which Yap/Taz were deleted 2 weeks prior to i.t. instillation of 3 mg/kg of *E*. *coli* LPS (Figure 4A). By 7 days after injury, while WT mice had largely resolved acute inflammation and injury, YT^del^ mice exhibited accumulation of patchy parenchymal fibrotic remodeling that persisted at 28 days after injury, long after LPS injury has typically resolved (Figure 4B). Throughout the LPS time course, YT^del^ mice had more severe and persistent injury compared with controls, with more damaged alveolar regions at 3, 7, and 14 days that persisted at 28 days after injury (Figure 4C). Immunofluorescence analysis of AT1 (Hopx) and AT2 (Sp-C) cell markers demonstrated a persistent loss of AT1 cells at 7 and 28 days after injury; while there was an initial decrease in AT2 cells at day 7, the AT2 cell population recovered by 28 days after injury in YT^del^ mice (Figure 4, D–F). These findings reveal that, in the acute LPS injury model, loss of Yap/Taz in AT2 cells led to a persistent defect in alveolar regeneration that resulted in increased lung injury, inflammation, and nonresolving alveolar collagen deposition. To define whether Yap/Taz deletion is affecting AT2 cell proliferation following either bleomycin or LPS injury, immunofluorescence analysis of proliferating AT2 cells (Ki67^+^/Sp-C^+^) was performed at 7 days after injury. Deletion of Yap/Taz resulted in reduced proliferating AT2 cells in both injury models, confirming the role of Yap/Taz in regulating cell proliferation (Supplemental Figure 5).

### AT2 cell deletion of Yap/Taz disrupts the adaptive innate immune response following LPS.

**Figure 5:**
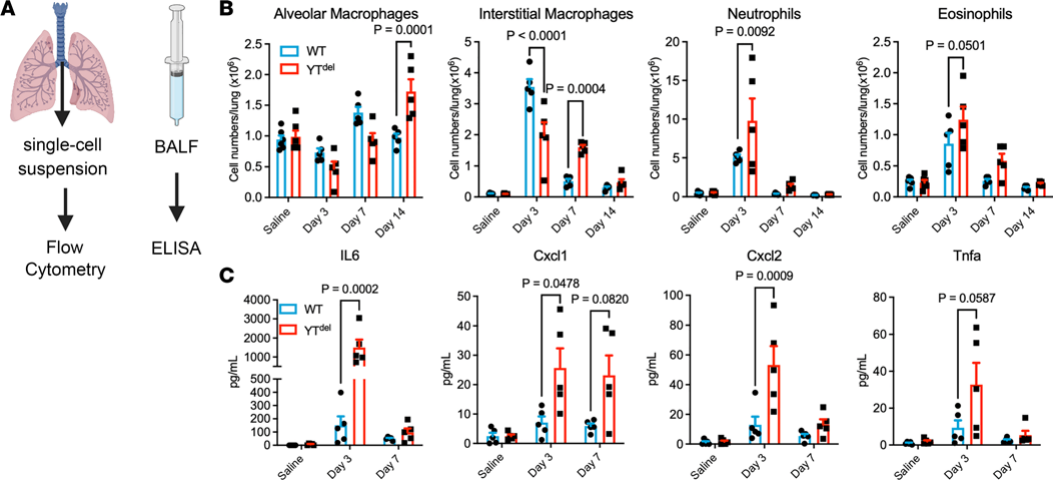
AT2 cell–specific deletion of YAP/TAZ leads to altered immune response after LPS injury. (**A**) Schematic showing experimental strategy to assess immune response in mice injured with LPS. (**B**) Quantification of immune cell types by flow cytometry in saline- or LPS-treated mouse lungs at 3, 7, or 14 days after injury. *n* = 6 saline-treated and *n* = 5 LPS-treated mice per group. (**C**) ELISA of immune cytokines isolated from bronchoalveolar lavage fluid from mice 3 or 7 days after LPS injury or saline controls. *n* = 5 mice per group. One-way ANOVA and Tukey’s post hoc comparison were used to assess statistical significance. Dots represent individual values per mouse.

Having observed augmented inflammatory cell recruitment in YT^del^ mice following LPS injury, we then sought to determine the mechanisms through which AT2 cell Yap/Taz regulated acute inflammatory responses. We performed flow cytometry of immune cell populations through a time course after LPS (Figure 5A). At day 3 after LPS, YT^del^ mice had reduced numbers of interstitial macrophages but increased neutrophils and eosinophils. By 7 days after injury, YT^del^ mice had increased interstitial macrophages, neutrophils, and eosinophils, indicating prolonged inflammatory response; lymphocyte numbers were similar between groups. These increased inflammatory populations had returned to near WT levels by 14 days after injury (Figure 5B and Supplemental Figure 6). ELISA of immune cytokines in bronchoalveolar lavage fluid isolated from these mice demonstrated that IL-6, Cxcl1, and Cxcl2 were all increased 3 days after injury, and they returned to WT levels by day 7 after injury (Figure 5C). These data indicate that loss of AT2 cell Yap/Taz worsens alveolar inflammation at least in part via exaggerated expression of neutrophil and macrophage chemotactic factors.

## Discussion

Functional repair and regeneration of the alveolar epithelium is a complex process requiring progenitor cells to proliferate and subsequently mature into structurally and functionally distinct AT2 and AT1 cells. We demonstrated that Yap/Taz activation in AT2 cells was essential for regeneration of both AT2 and AT1 cells during alveolar repair. Further, using complementary models of alveolar injury, we found that AT2 cell Yap/Taz activity restrained innate immune activation and that, without Yap/Taz activation in AT2 cells, ineffective alveolar epithelial repair resulted in persistent parenchymal injury and fibrosis. Our results here indicate that, following injury, YT^del^ lineage–labeled AT2 cells failed to proliferate or give rise to morphologically or transcriptionally mature AT1 cells, suggesting that, during injury repair, at least transient Yap/Taz activity was required to mediate both AT2 proliferative responses and AT1 cell maturation.

Our results build upon and extend recent studies that highlight the complex, dynamic, and cell type–specific roles that the Hippo-Yap/Taz pathway plays in lung development, homeostasis, and injury responses. We have previously demonstrated aberrant and persistent YAP activation in the IPF lung epithelium (6), while several groups have implicated YAP/TAZ as mediators of fibroblast activation driven by mechanical forces (24–27). This has led to interest in YAP/TAZ antagonism as a potentially novel antifibrotic strategy in the lung, similar to investigations in other organs (28–33). Together with complementary studies from our group and several others, our findings suggest that a more nuanced consideration of the Hippo-Yap/Taz pathway is likely required. This necessity for temporal and cell type–specific regulation supports a concept analogous to the airway epithelium, in which loss of Yap/Taz activity leads to failure of progenitor cell expansion and inhibits epithelial repair, but persistent activation and inability to downregulate Yap/Taz in airway epithelial cell results in basal cell metaplasia and prevents terminal differentiation/maturation of secretory and multiciliated cells, also leading to failure to repair (11, 12, 14).

During lung development, we and others have previously shown that Yap/Taz is required for AT1 cell commitment (5, 15, 34, 35), although recent reports have yielded inconsistent results as to the specific roles of Yap and Taz at different developmental stages and using different models (4, 36, 37). Our results here indicate that, following single-dose bleomycin or LPS injury, combined deletion of Yap and Taz in AT2 cells prevents AT2 proliferation and AT1 differentiation, indicating that some level of Yap/Taz activity is required for both of these critical aspects of alveolar regeneration. Based on our single-cell RNA-Seq data and recent work by Warren et al. (36), Yap/Taz deletion in AT2 cells appears to directly impair AT1 differentiation during the reparative phase after injury, resulting in the persistence of an intermediate alveolar epithelial cell state that recent reports indicate may be pathologic (38, 39). These data complement prior work from our group and others that suggests deletion of Yap/Taz in AT2 cells leads to an “exaggerated AT2 cell phenotype” (16), suggesting that, even under homeostatic conditions, low-level Yap/Taz activity may be involved in “priming” AT2 cells for differentiation. In addition, deletion of Yap/Taz in Hopx lineage-labeled cells resulted in reversion to an AT2-like cellular profile (15). Together, these findings suggest that Yap/Taz is essential for AT2 cell proliferation, AT1 differentiation, and AT1 cell maturation/maintenance. While it is somewhat counter intuitive that these distinct processes may all require Yap/Taz, recent studies raise the possibility that different Yap/Taz functions could be regulated through differential binding partner affinity. This concept is supported by recent work finding a dynamic interaction of Yap/Taz with NKX2-1 and/or NFIB and the AT1-associated KLF5 during postnatal lung development (4, 16, 34, 40, 41).

We also observed that, in two different models of sterile alveolar injury, loss of Yap/Taz activity in AT2 cells was accompanied by increased and persistent alveolar inflammation. These findings are consistent with previous work that found deletion of Yap/Taz leads to an increased inflammatory response to *Streptococcus pneumoniae* infection through impaired IκBα expression and exaggerated NF-κB signaling (3). It is not yet clear whether these effects on acute inflammation are due to direct effects of Yap/Taz on regulation of inflammatory programs in AT2 cells, are consequences of ineffective alveolar repair, and/or are both. Beyond the role of AT2 cell type–specific Yap/Taz activity regulating the immune response of the injured lung, Yap/Taz also plays a role, which has recently been reviewed (42), in immune cells through several mechanisms.

In addition to autonomous effects on epithelial cell fate/function and immune regulation, we also found that deletion of Yap/Taz in AT2 cells worsens fibrotic remodeling following bleomycin injury and was associated with enhanced fibroblast activation and proliferation. More surprisingly, we observed that, without AT2 cell Yap/Taz, low-dose LPS led to alveolar collagen deposition and persistent parenchymal remodeling out to at least 28 days after LPS. Together, these data add to a growing body of evidence that suggests specifically that inability to effectively mature AT1 cells is sufficient to promote lung parenchymal fibrosis (43, 44), although the specific mechanism through which the pathologic cellular crosstalk drives this process is not yet clear. Our analysis of receptor-ligand crosstalk demonstrated loss of FGF and IGF signaling between the epithelium and mesenchyme. Potentially complicating this aberrant fibroblast activation in the fibrotic lung, there is an increase in stretch response as the lung stiffens during fibrotic remodeling, and fibroblast-focused studies have shown that Yap/Taz are activated in the presence of mechanical stretch (24–26, 45). Additional recent findings have shown that Yap/Taz activation is involved in a stretch-associated modulation of chromatin remodeling (46) by the extracellular matrix, raising the possibility that failure of alveolar epithelial repair alters the local mechanical environment to promote Yap/Taz activation in alveolar fibroblasts and/or that adjacent alveolar epithelial cells to promote tissue fibrosis could generate a feed-forward mechanism. Yap/Taz have also been shown to interact with several other pathways that have been implicated in promoting fibrosis, including TGF-β (47, 48), Wnt (49–52), FGF (53), Notch (54), and mTOR/Pi3K (55).

There are several limitations of this data. First, our studies rely on genetic strategies using transgenic mice to modulate Yap/Taz activity in AT2 cells, and the dosing and timing of tamoxifen administration likely impacts the spectrum of recombined cells. Our lineage-tracing studies suggest that our recombination efficiency was high in this model, and incomplete recombination would likely bias between-group differences toward the null. There is also an inherently limited ability to discern AT2 cell–autonomous effects of Yap/Taz deletion from more general consequences of ineffective alveolar repair and loss of epithelial barrier. Future studies carefully interrogating the temporal evolution of barrier function and the immune response using lineage-labeled mice in the LPS injury model may yield additional insight into primary versus secondary mechanisms. We also did not seek to determine separable roles of Yap versus Taz in AT2 cells following injury, since recent reports demonstrate some redundancy in these roles or at least that deletion of Yap activates Taz (36).

Together, our findings support the concept that the Hippo-Yap/Taz pathway must be tightly regulated to facilitate adaptive repair and that either failure to activate the pathway to initiate alveolar repair or failure to downregulate the pathway to promote alveolar epithelial cell homeostasis both are maladaptive and lead to progressive fibrotic remodeling. These findings collectively support the concept that future therapeutic strategies to modulate YAP/TAZ activity have potential to promote lung regeneration but that timing and cell targeting considerations will be important.

## Methods

### Animal husbandry and deletion of YAP/TAZ.

*SftpcCre^ert2(blh)^Yap^fl/fl^Taz^fl/fl^* mice were crossed with *Yap^fl/fl^Taz^fl/fl^* mice to generate experimental mouse cohorts similar to our previous postnatal studies (4). Cre^–^ littermates are used for WT controls. *SftpcCre^ert2rosatdTomato^Yap^fl/fl^Taz^fl/fl^* mice were crossed with *Yap^fl/fl^Taz^fl/fl^* mice to generate mice for lineage-tracing studies. *SftpcCre^ert2rosetdTomato^* were used as lineage-trace control mice. Mice were administered tamoxifen 100 mg/kg or corn oil control by i.p. injection either 2 weeks prior to, or at the same time as, respective injury to induce gene deletion and lineage tracing. For assessment of adaptive repair in bleomycin-injured mice and as additional control mice for injury experiments, 8- to 10-week-old C57BL/6J mice were ordered from The Jackson Laboratory (stock no. 000664).

### Lung injury.

Bleomycin (0.08 IU or 0.04 IU) or *E*. *coli* LPS (3 mg) were suspended in sterile saline and i.t. administered in 100 μL volumes, and equivalent volumes of saline were used for controls. Mice were weighed weekly to assess health throughout the experiments. Mice were sacrificed at indicated time points, with the left lung being inflation fixed in 10% buffered formalin and the right lung being processed for Sircol collagen analysis or isolation of Cd326^+^ cells for RNA analysis.

### IHC analysis.

Lungs were inflation fixed with 10% buffered formalin at 25 cm H_2_0 pressure and fixed at 4°C overnight. Lungs were then processed and paraffin embedded, and 5 mm sections were used for all histological stains. Immunofluorescence was performed using antibodies (described in Supplemental Table 1) as follows. Antigen retrieval was done using a rice cooker and 1***×*** Citrate Buffer (pH 6, Sigma Aldrich, C9999); slides were then washed in deionized water. Slides were blocked in 5% BSA for 1 hour at room temperature and then incubated in primary antibodies overnight. Samples were then washed 3***×*** in PBST and then incubated in secondary antibody with DAPI diluted in PBST for 2 hours. Slides were then washed in PBST 3***×***, and cover slips were added with mounting media. For immunofluorescence analysis, samples were imaged in a Keyence BZ-X710 inverted fluorescence microscope, and images were taken on a 20***×*** objective with 5–6 nonoverlapping fields of view. Image analysis was performed with HALO image analysis software for automated quantification of respective cell states. Histological analysis of lung injury was quantified using ImageJ (NIH) on samples stained with Masson’s trichrome stain, using a grid placed over the image assessing points at intersecting lines for lung injury (18).

### Single-cell RNA-Seq.

Single-cell suspensions were generated as previously described. Right lung lobes were extracted and incubated in dispase II (Roche), collagenase (Sigma-Aldrich), and DNase in phenol-free DMEM (Thermo Fisher Scientific). Lungs were disaggregated using GentleMacs cell dissociator with C-tubes (Miltenyi Biotec), and cell suspensions were passed through 100 μm and 70 μm filters to isolate single-cell suspensions. Once single-cell suspensions were achieved, RBCs were depleted using ACK buffer and cells were fixed following PARSE Biosciences Fixation User Manual V1.3.0 protocol. Libraries were sequenced on a NovaSEQ6000 targeting 50,000 reads per cell, and demultiplexing was performed using the PARSE pipeline (v0.9.6) with default parameters. The read mapping and alignment were based on Mouse GRCm39 genome and GENCODE M28 annotation.

### Single-cell RNA-Seq analysis.

Single-cell RNA-Seq data were analyzed through a standard Scanpy (56) (v1.9.1) workflow similar to our prior work (57). Following quality-control filtering (excluding cells with < 500 or > 5,000 genes and cells with > 10% mitochondrial reads), data were normalized and scaled. Mitochondrial and ribosomal percentages were evaluated as regression variables, followed by principal component analysis, Harmony integration (58), neighborhood graph calculation, and UMAP embedding using the first 45 principal components (PCs) as well as recursive leiden-clustering/subclustering. Heterotypic doublet clusters were identified using scrublet (59) by coexpression of lineage-specific marker genes, and cell annotation was performed manually, informed by published reference data sets (60, 61). Cell type–specific differential expression was performed using the Wilcoxon test. LIANA (23) and Tensor-cell2cell (22) were used to assess gene ontology–associated programs and ligand-receptor crosstalk to define disrupted cell-to-cell interactions.

### Flow cytometry.

To obtain single-cell suspensions from lung tissue, lungs were perfused with sterile PBS and removed en bloc, and perfused lungs were digested in RPMI medium containing collagenase XI (0.7 mg/mL; Sigma-Aldrich) and type IV bovine pancreatic DNase (30 μg/mL; Sigma-Aldrich). RBCs were lysed with RBC Lysis Buffer (BioLegend) as described elsewhere (62). Single-cell suspensions were incubated with a Fc receptor block (553141, BD Bioscience) to reduce nonspecific antibody binding. The flow cytometry panel used to identify immune cell subtypes is shown in Supplemental Figure 3; in short, antibodies used in these experiments included CD45-Brilliant Violet 650 (catalog 103151), Ly6G-APC/Cyanine7 (catalog 127623), F4/80-PE/Cy5 (catalog 123111), MerTK-Brilliant Violet 605 (catalog 151517), CD11c-AF-700 (catalog 117320), CD11b- PE/Cyanine7 (Cat.101216), MHCII-FITC (catalog 107605), and CD64-APC (catalog 139306) from BioLegend as well as SiglecF-PE (catalog 552126) from BD Biosciences. Dead cells were excluded using DAPI (catalog MBD0015, MilliporeSigma). Flow cytometry was performed using BD LSR II and BD FACS Aria III flow cytometers (BD Biosciences), and data were analyzed with FlowJo software.

### Bronchoalveolar lavage fluid isolation.

I.t. cannulation was used to wash lungs 5 times with 1 mL sterile PBS. Immune cell populations were assessed by cytospin and subsequent DiffQuick staining or through the above flow cytometry panel.

### Statistics.

Statistics were performed in GraphPad Prism. Data are shown as mean ± SEM. Whiskers in box-and-whisker plots are minimum and maximum values. Statistical significance was determined by 1-way ANOVA with Tukey’s post hoc comparisons. Any deviation in this approach is noted in the respective figure legends. Mantel-Cox analysis was used to determine significance of survival curves. Wilcoxon test was used to determined differentially expressed genes in RNA-Seq analysis. All *P* values considered significant (*P* < 0.05) are written in the graphs to show comparisons.

### Study approval.

These studies were approved by Vanderbilt University Medical Center’s IACUC.

### Data availability.

Raw genomic data are available through the Gene Expression Omnibus (GEO)/Short Read Archive (SRA; GSE243135). Code use for single-cell RNA-Seq analysis is available at https://github.com/KropskiLab/digiovanni_2023 Output files generated from LIANA are available in the supplemental GSEA file. Values used to generate graphs are available in the Supporting Data Values file.

## Author contributions

GTD, WH, TPS, TSB, JAK, and JJG designed research studies. GTD, WH, TPS, CJT, NMG, UKS, CLC, ASM, MMD, JHJ, SSG, and JJG conducted experiments. GTD, WH, CJT, DSN, YL, JAK, and JJG acquired data. GTD, WH, YL, VVP, TSB, JAK, and JJG analyzed data. GTD, TSB, JAK, and JJG wrote the manuscript. All authors edited to the manuscript.

## Supplementary Material

Supplemental data

Supplemental GSEA

Supporting data values

## Version 1 09/07/2023

In-Press Preview

## Version 2 10/09/2023

Electronic publication
