# Supplementary material for: Epithelial Yap/Taz are required for functional alveolar regeneration following acute lung injury
Source: JCI Insight. 2023 Sep 7;8(19):e173374. doi: 10.1172/jci.insight.173374 (PMC10629815; doi:10.1172/jci.insight.173374)
Supplement: Supplemental data [file jciinsight-8-173374-s055.pdf]

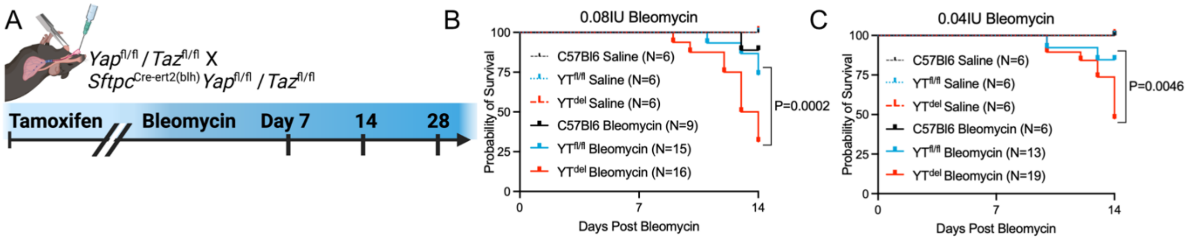

Supplemental Figure S1: A) Schematic of bleomycin lung injury, in which mice are treated with tamoxifen 2-weeks prior to injury with a planned 28-day recovery. **B,C)** Survival curves of mice treated with 0.08IU bleomycin (B) or 0.04IU bleomycin (C). Mantel-Cox test was used to determine statistical significance.

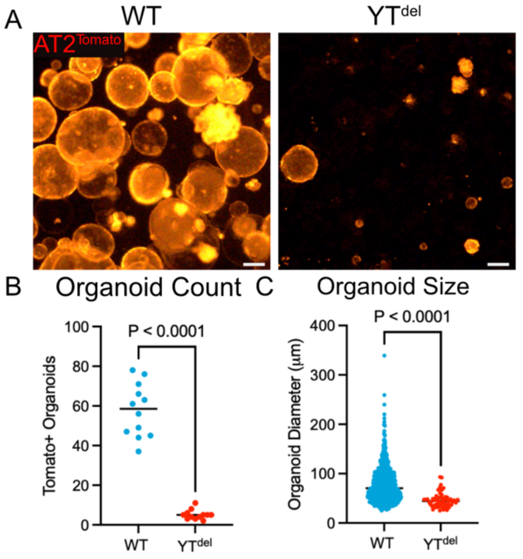

Supplemental Figure S2: YT^{del} generate fewer and smaller organoids in feeder-free culture. **A.)** Fluorescent image of Tomato lineage labeled AT2 cells cultured in SFFFM media for 14-days. **B.)** Quantification of total number of organoids per well and **C.)** organoid size analysis of organoids larger than 25µm. Statistical analysis performed with an unpaired t-test. Scale bar represents 25µm. Organoids were grown from 3 mice per group, 4 wells per mouse.

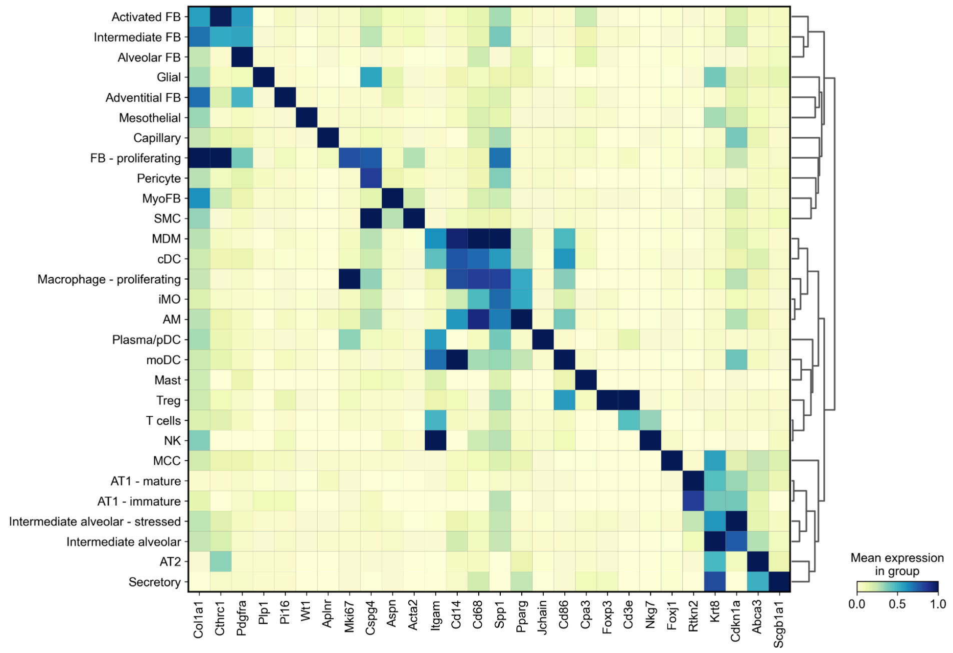

Supplemental Figure S3: Cell types identified in single-cell RNA sequencing analysis and expression of cell-type specific markers.

Flow gating strategy

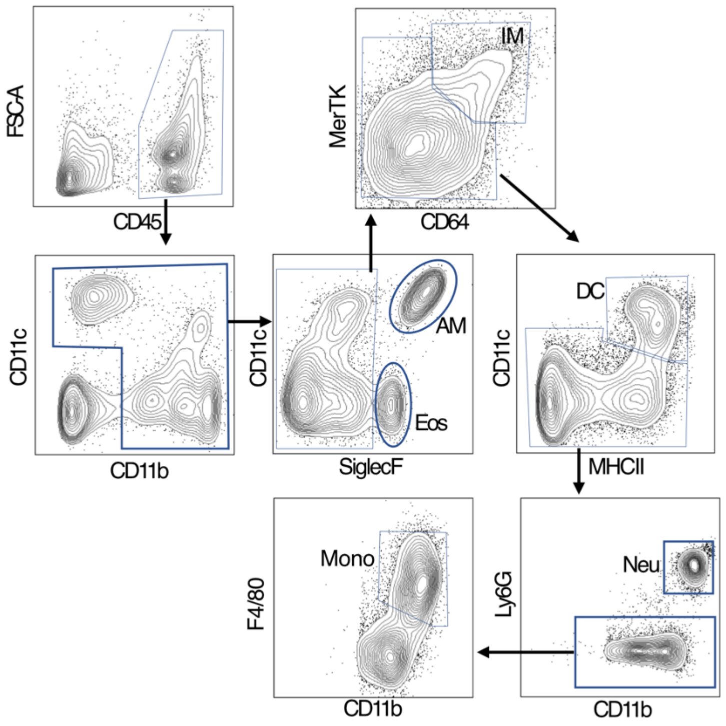

Supplemental Figure S4: Flow Cytometry gating strategy of immune cells isolated from mouse lung single-cell suspensions.

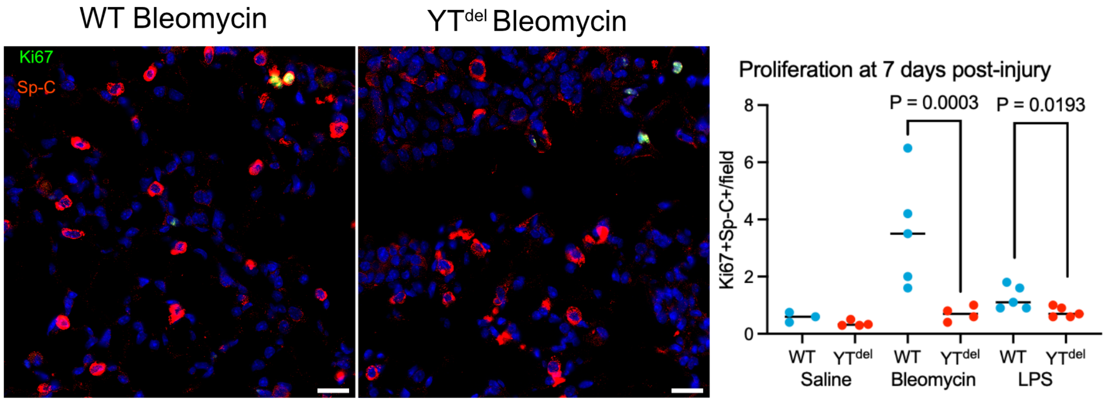

Supplemental Figure S5: Analysis of proliferating AT2 cells following injury. Immunofluorescence analysis of proliferating Ki67+ (green) Sp-C+ AT2 (red) cells at 7 days post bleomycin or LPS injury. N=3 WT saline, N=4 YT^{del} saline and bleomycin, N=5 WT bleomycin, WT LPS and YT^{del} LPS treated mice. Statistical significance determined by one-way ANOVA and Tukey's post-hoc comparison.

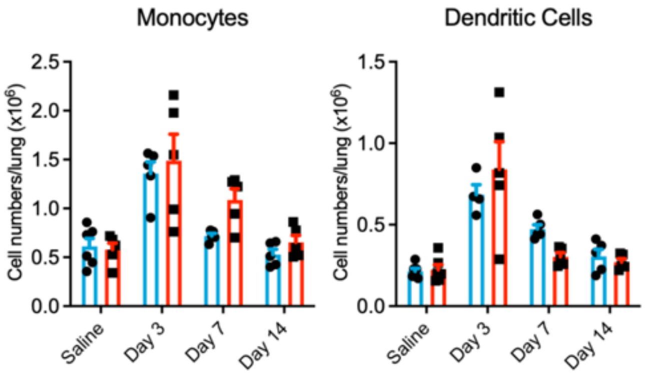

Supplemental Figure S6: Flow cytometry of total numbers of monocytes and dendritic cells isolated from LPS injured lungs at day 3, 7, and 14 after injury. Cell numbers were not significantly different based on genotype determined by one-way ANOVA and Tukey's post-hoc comparison. N=6 saline and N=5 LPS mice per group.

Antibody	Company	Host Species	Catalog #	Concentration
SPC	Seven Hills Bioreagents<br><br>Abcam	Rabbit<br><br><br>Rabbit	WRAB-9337<br><br><br>AB90716	1:400<br><br><br>1:100
YAP	Seven Hills Bioreagents<br><br>Cell Signaling Technology	Rabbit<br><br><br>Rabbit	WRAB-1549<br><br><br>4912S<br>14074S	1:100<br><br><br>1:100<br>1:100
TAZ	Cell Signaling Technology	Rabbit	83669S	1:100
HOPX	Santa Cruz Biotechnology	AF647 Conj.	sc-398703	1:100
Ki67	Cell Signaling Technology	AF647 Conj.	12075S	1:100
TdTomato	SICGEN	Goat	AB8181-200	1:500

Supplemental Table S1: Antibodies used for immunofluorescence analysis with respective catalog information and concentration used.
