# Supplementary figures and images for: Epithelial Yap/Taz are required for functional alveolar regeneration following acute lung injury
Source: JCI Insight. 2023 Sep 7;8(19):e173374. doi: 10.1172/jci.insight.173374 (PMC10629815; doi:10.1172/jci.insight.173374)

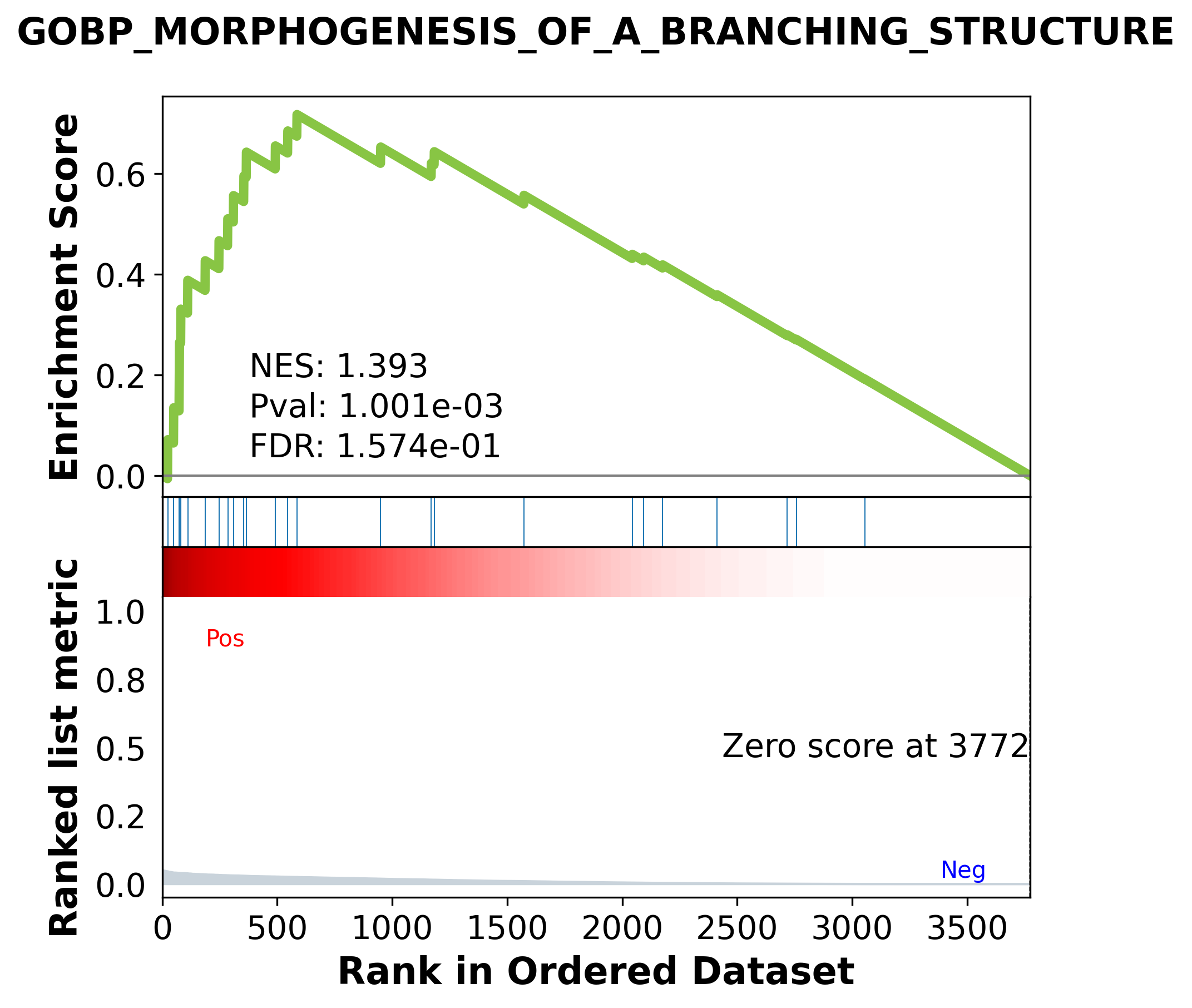

Supplement: Supplemental GSEA [file jciinsight-8-173374-s056.zip › GSEA/Factor 3/prerank/GOBP_MORPHOGENESIS_OF_A_BRANCHING_STRUCTURE.png]

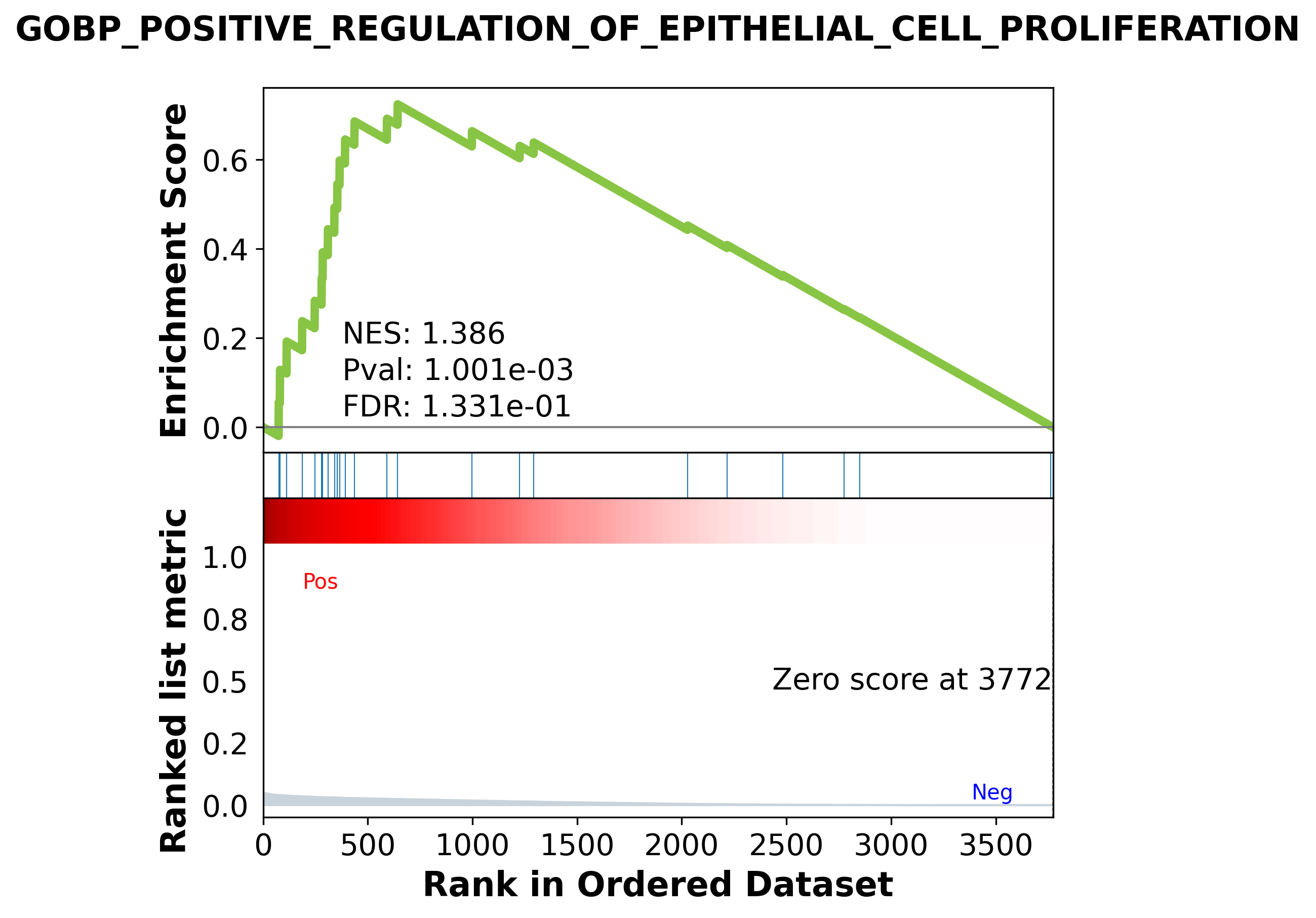

Supplement: Supplemental GSEA [file jciinsight-8-173374-s056.zip › GSEA/Factor 3/prerank/GOBP_POSITIVE_REGULATION_OF_EPITHELIAL_CELL_PROLIFERATION.png]

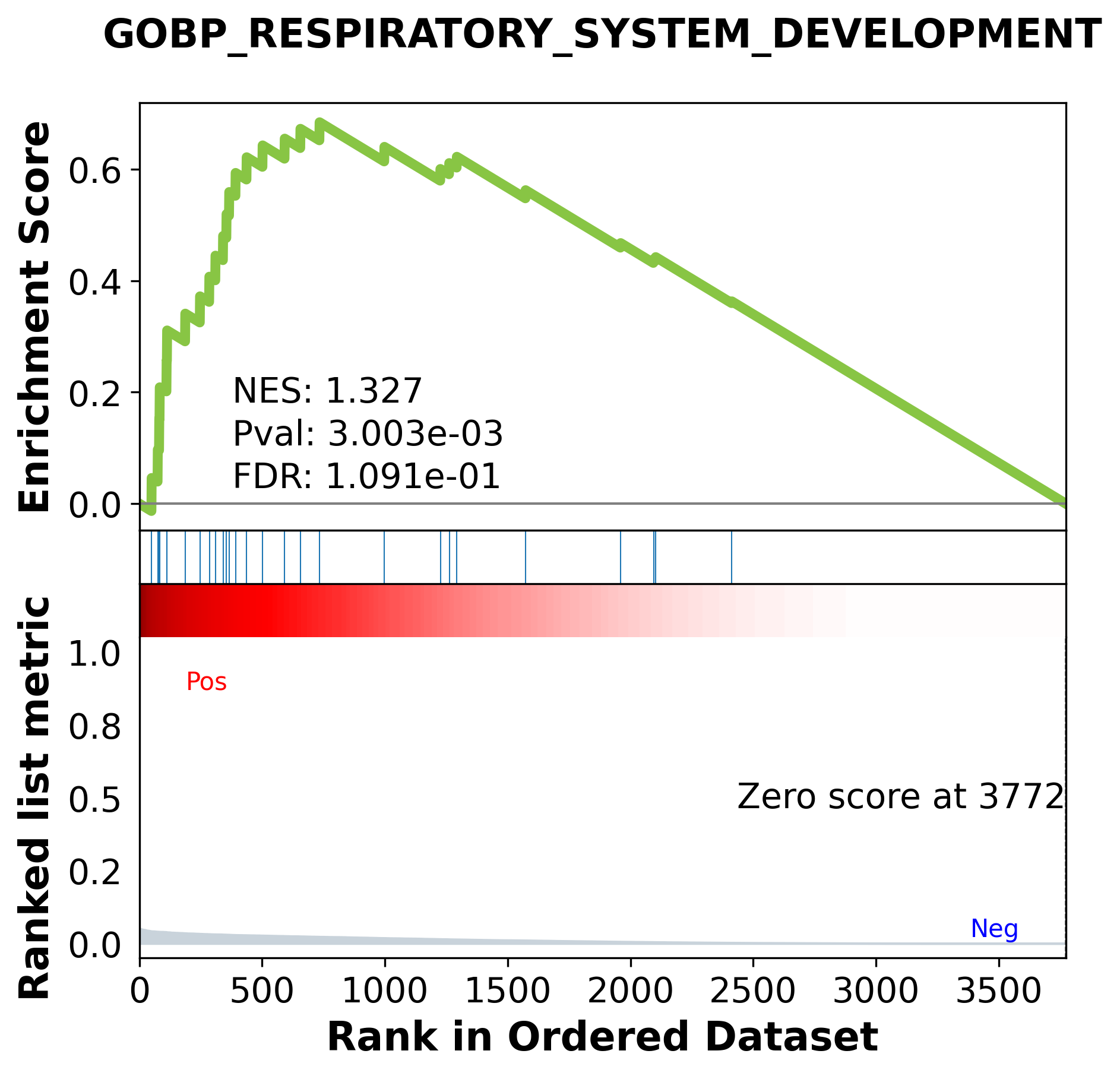

Supplement: Supplemental GSEA [file jciinsight-8-173374-s056.zip › GSEA/Factor 3/prerank/GOBP_RESPIRATORY_SYSTEM_DEVELOPMENT.png]

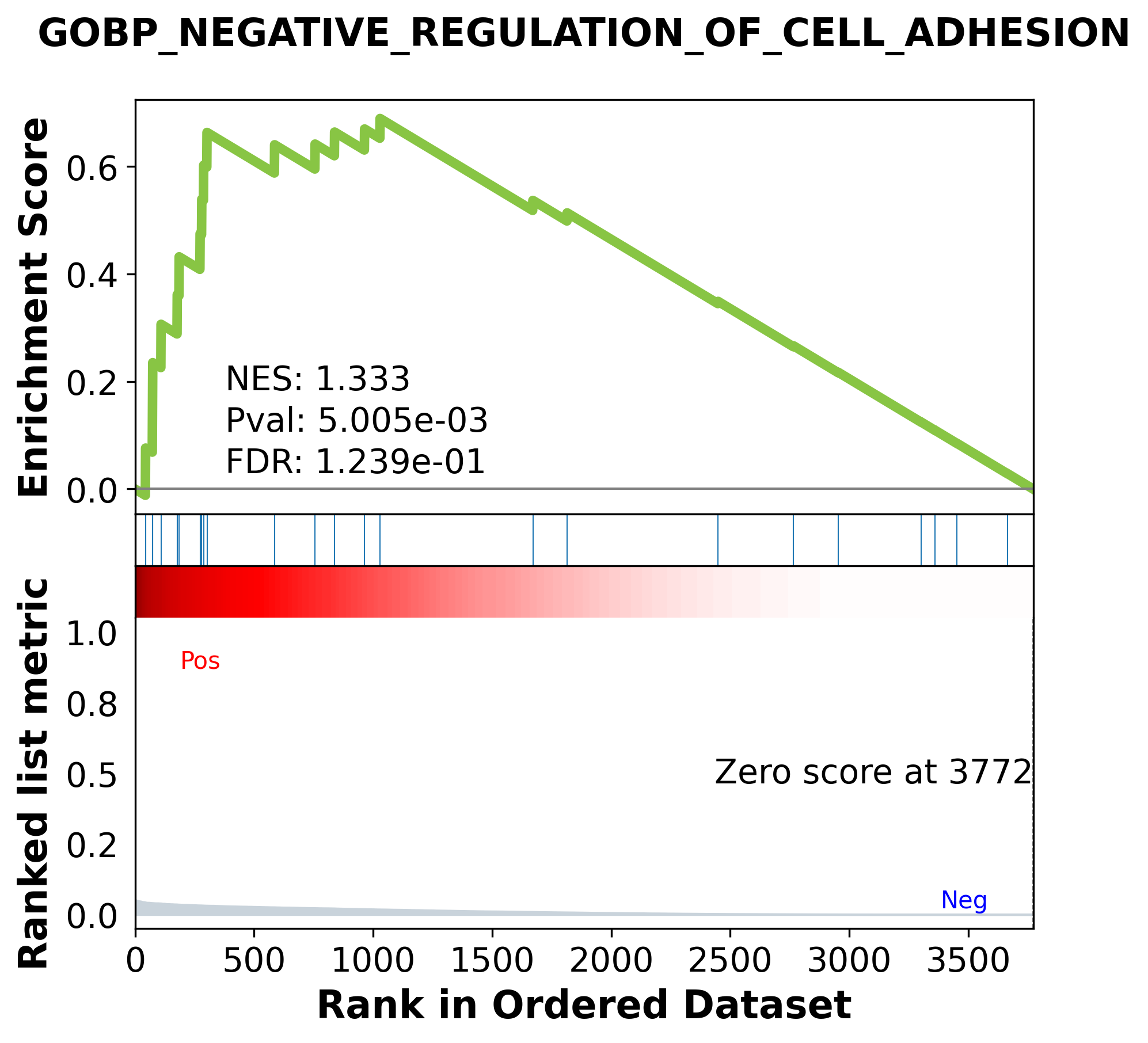

Supplement: Supplemental GSEA [file jciinsight-8-173374-s056.zip › GSEA/Factor 3/prerank/GOBP_NEGATIVE_REGULATION_OF_CELL_ADHESION.png]

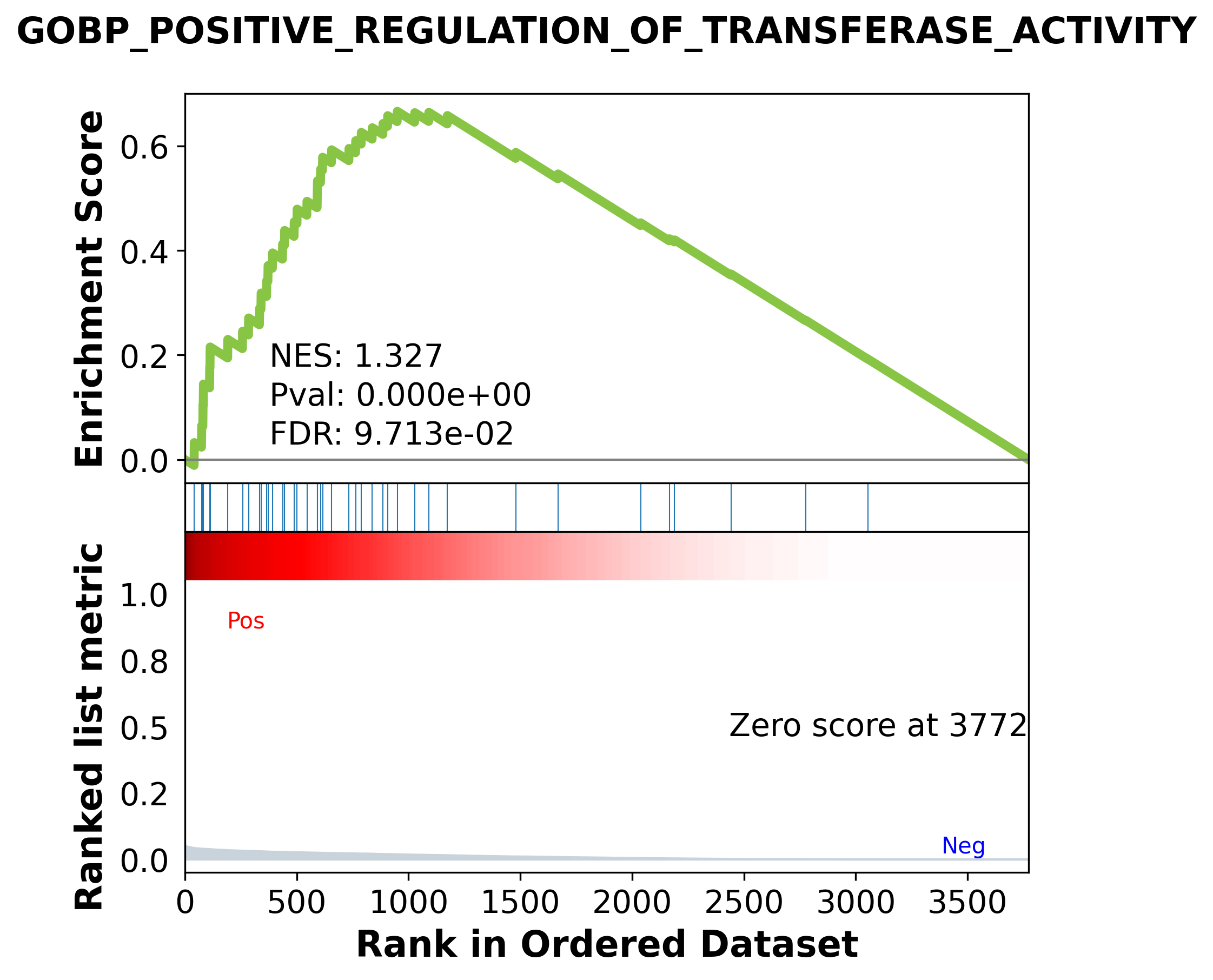

Supplement: Supplemental GSEA [file jciinsight-8-173374-s056.zip › GSEA/Factor 3/prerank/GOBP_POSITIVE_REGULATION_OF_TRANSFERASE_ACTIVITY.png]

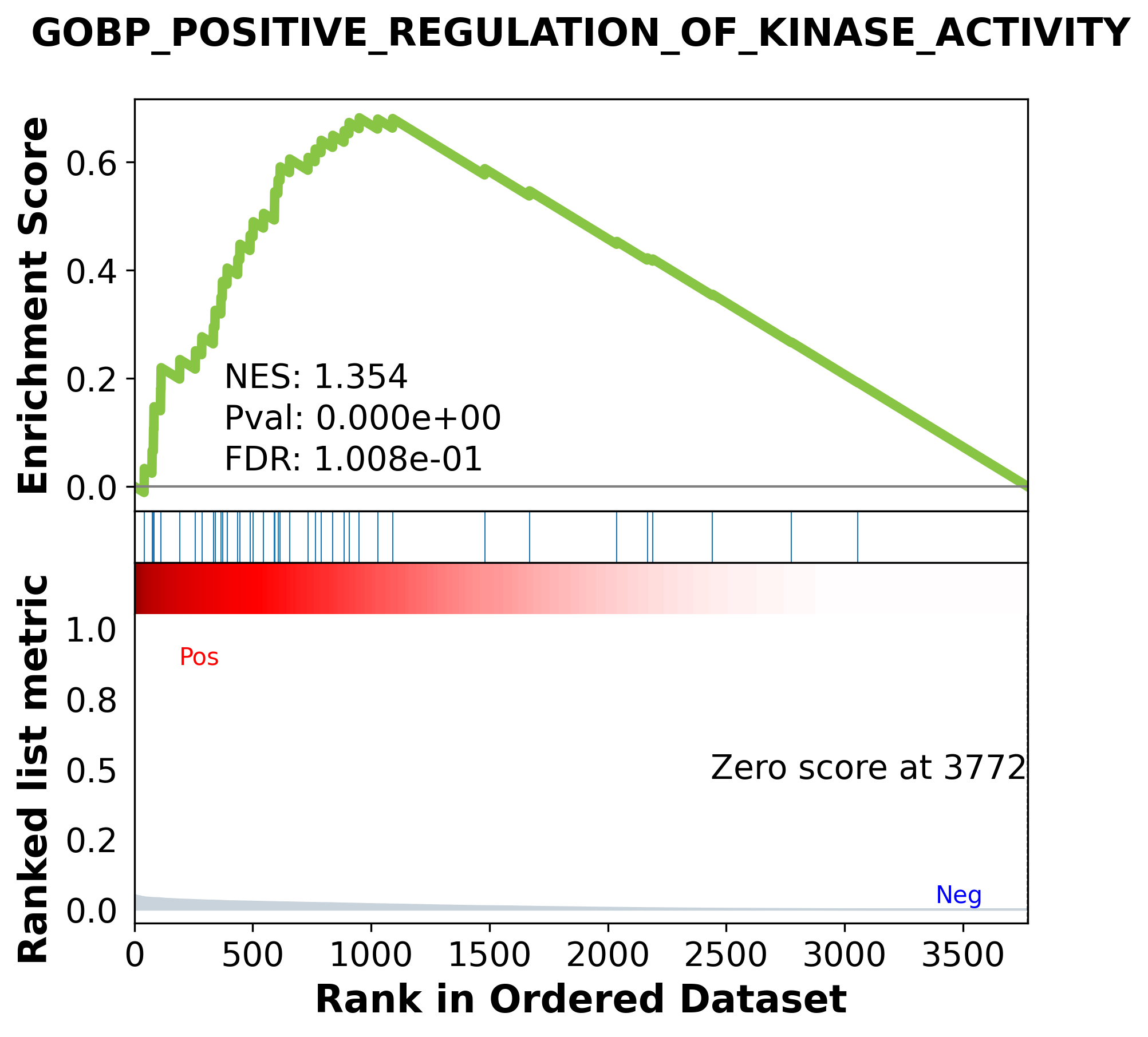

Supplement: Supplemental GSEA [file jciinsight-8-173374-s056.zip › GSEA/Factor 3/prerank/GOBP_POSITIVE_REGULATION_OF_KINASE_ACTIVITY.png]

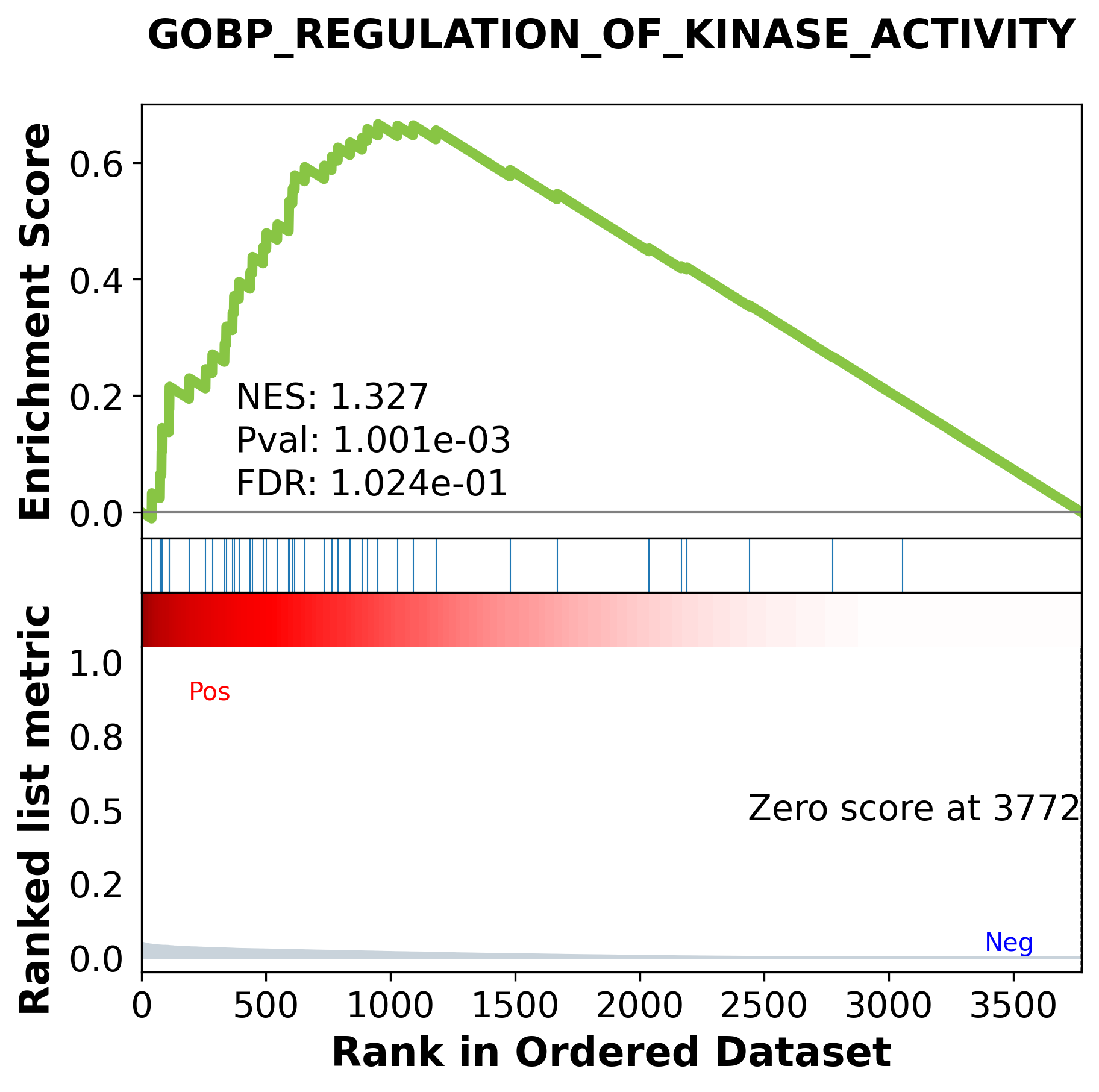

Supplement: Supplemental GSEA [file jciinsight-8-173374-s056.zip › GSEA/Factor 3/prerank/GOBP_REGULATION_OF_KINASE_ACTIVITY.png]

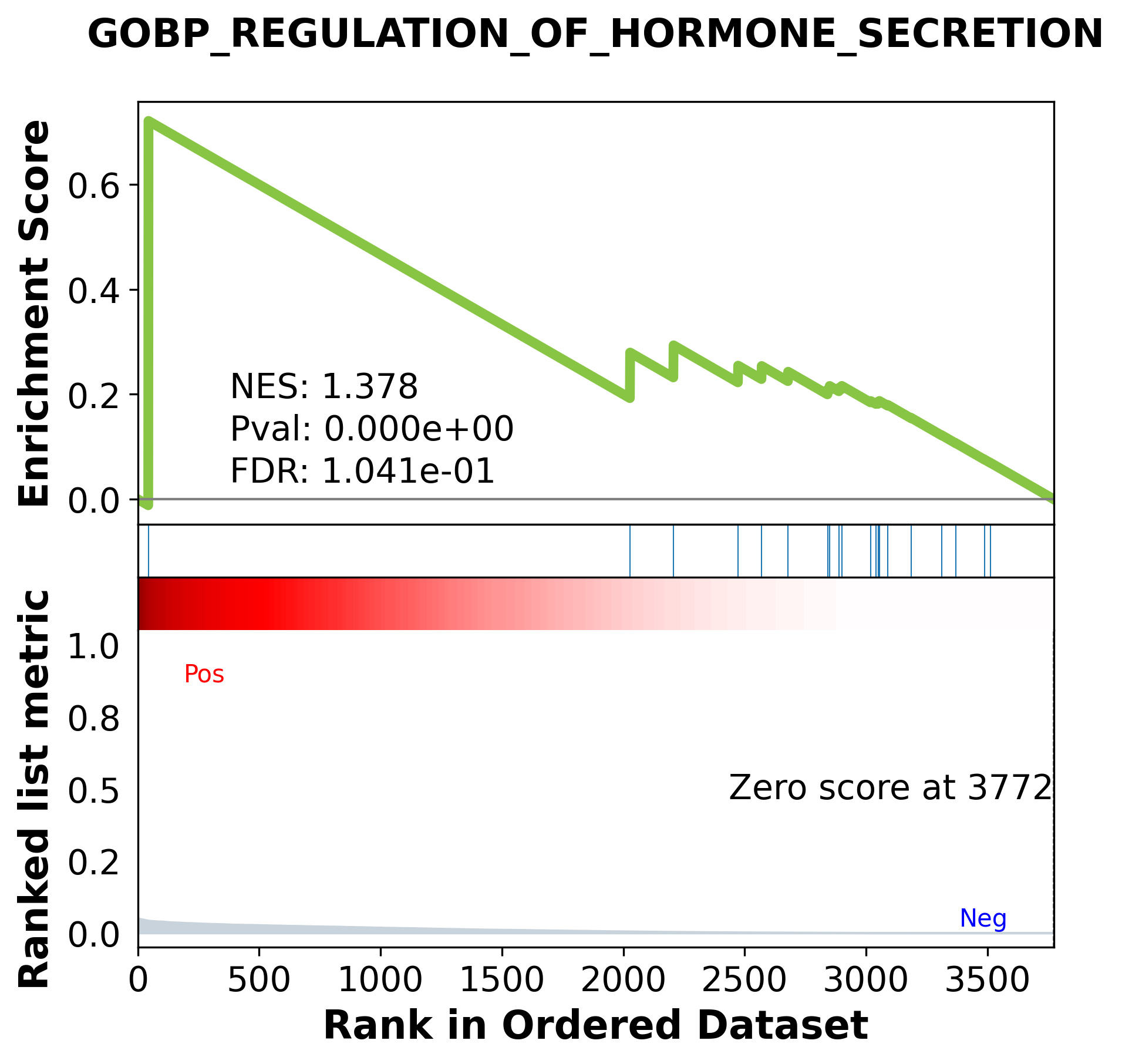

Supplement: Supplemental GSEA [file jciinsight-8-173374-s056.zip › GSEA/Factor 3/prerank/GOBP_REGULATION_OF_HORMONE_SECRETION.png]

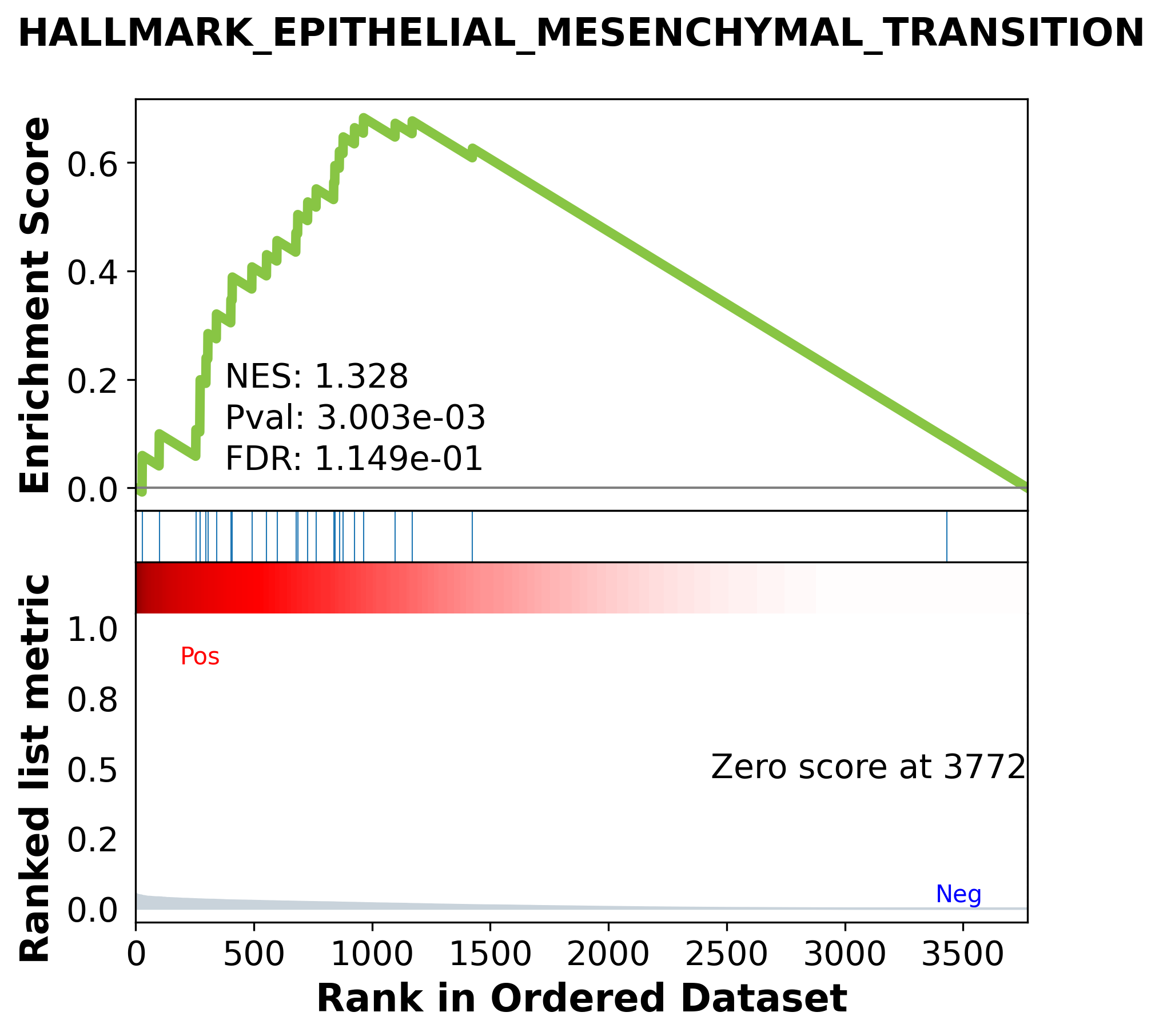

Supplement: Supplemental GSEA [file jciinsight-8-173374-s056.zip › GSEA/Factor 3/prerank/HALLMARK_EPITHELIAL_MESENCHYMAL_TRANSITION.png]

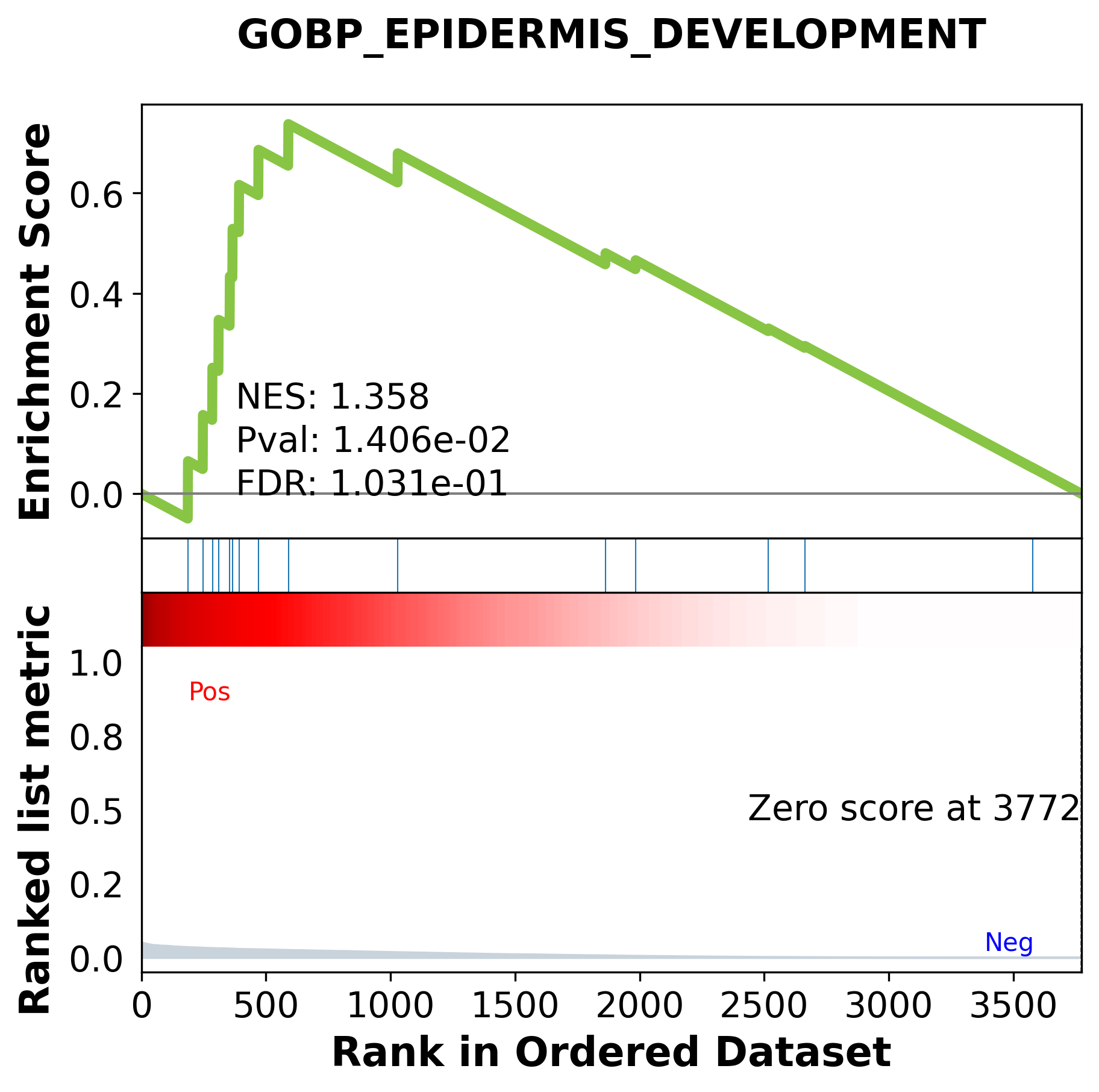

Supplement: Supplemental GSEA [file jciinsight-8-173374-s056.zip › GSEA/Factor 3/prerank/GOBP_EPIDERMIS_DEVELOPMENT.png]

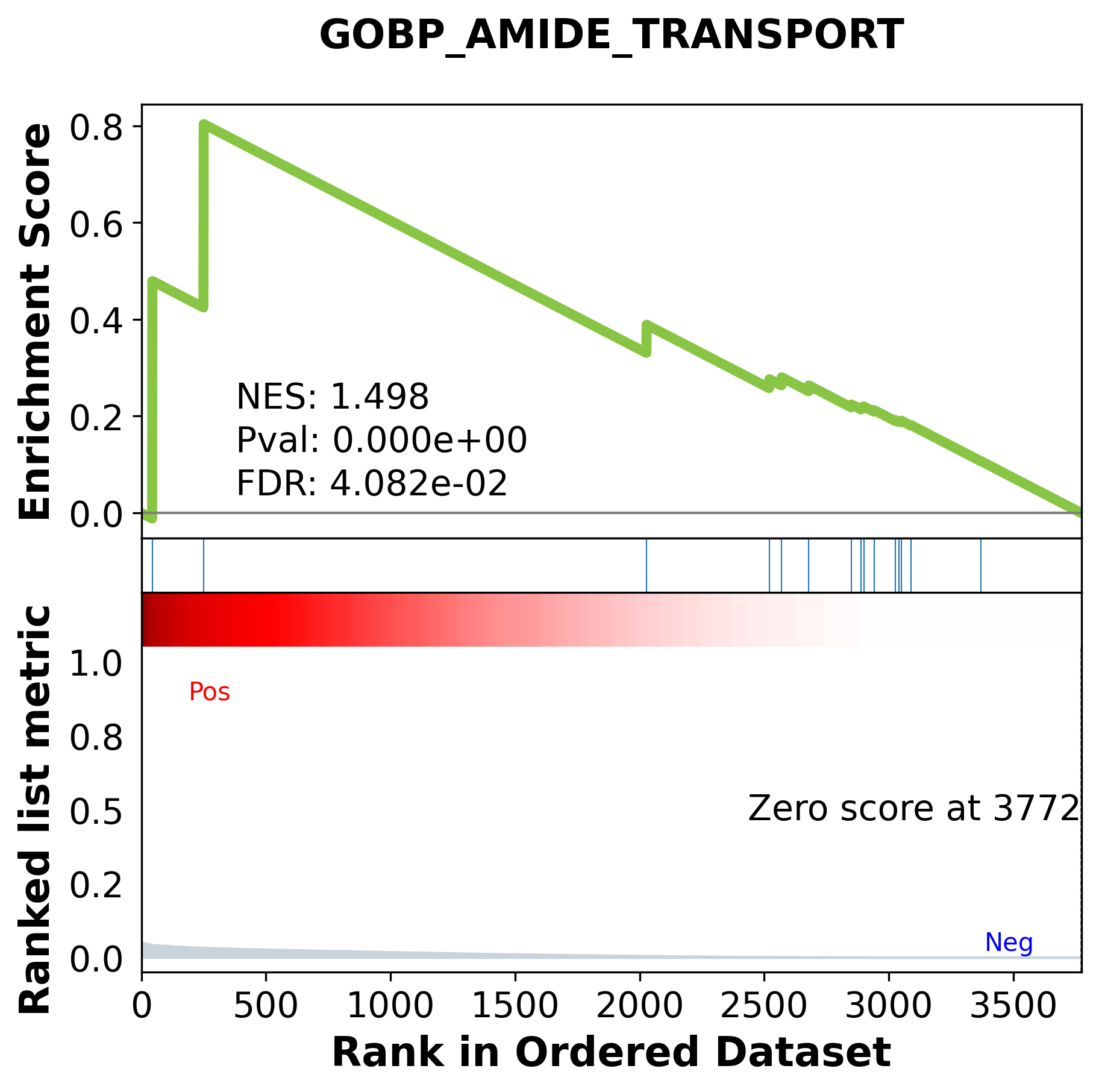

Supplement: Supplemental GSEA [file jciinsight-8-173374-s056.zip › GSEA/Factor 3/prerank/GOBP_AMIDE_TRANSPORT.png]

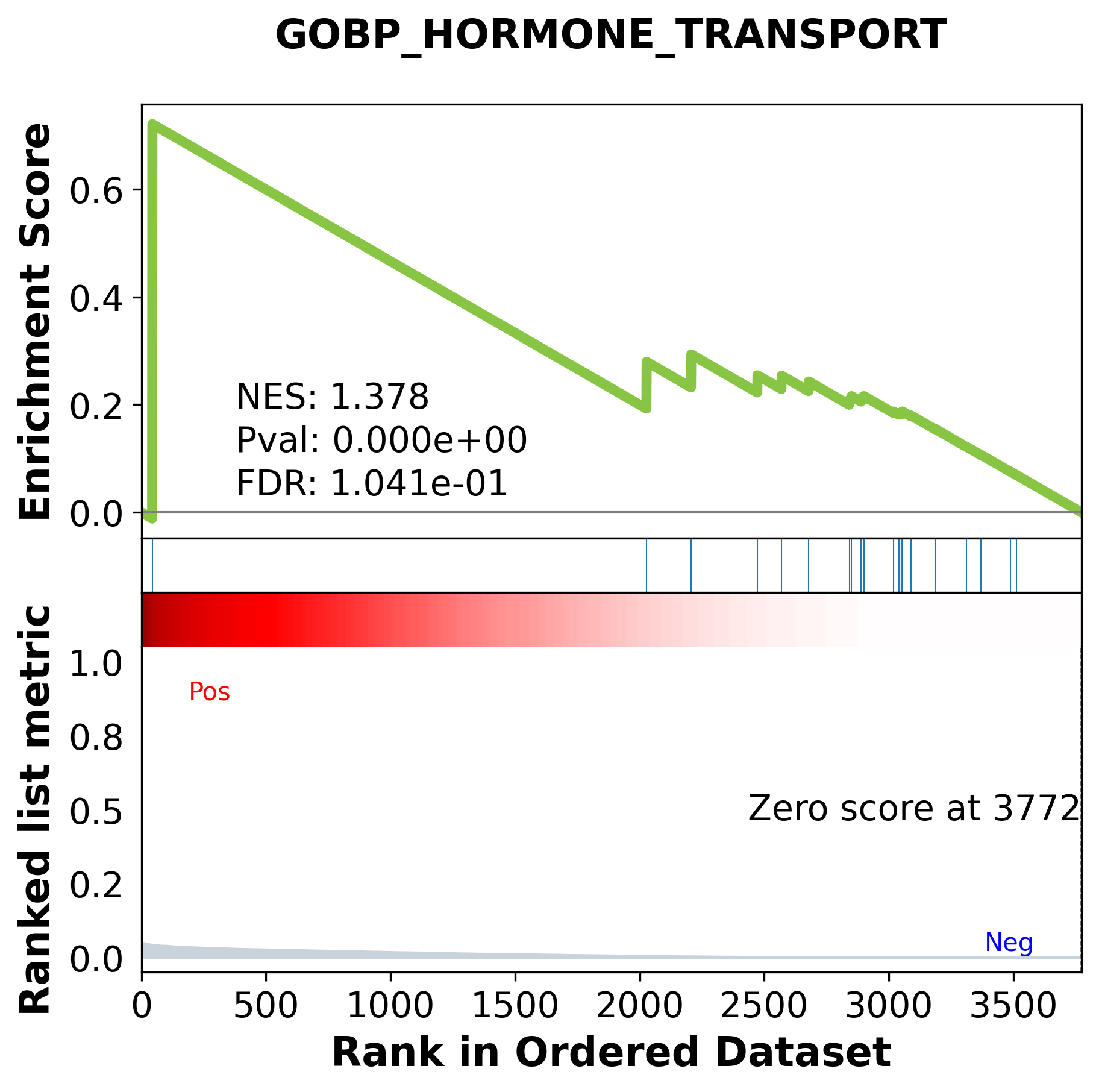

Supplement: Supplemental GSEA [file jciinsight-8-173374-s056.zip › GSEA/Factor 3/prerank/GOBP_HORMONE_TRANSPORT.png]

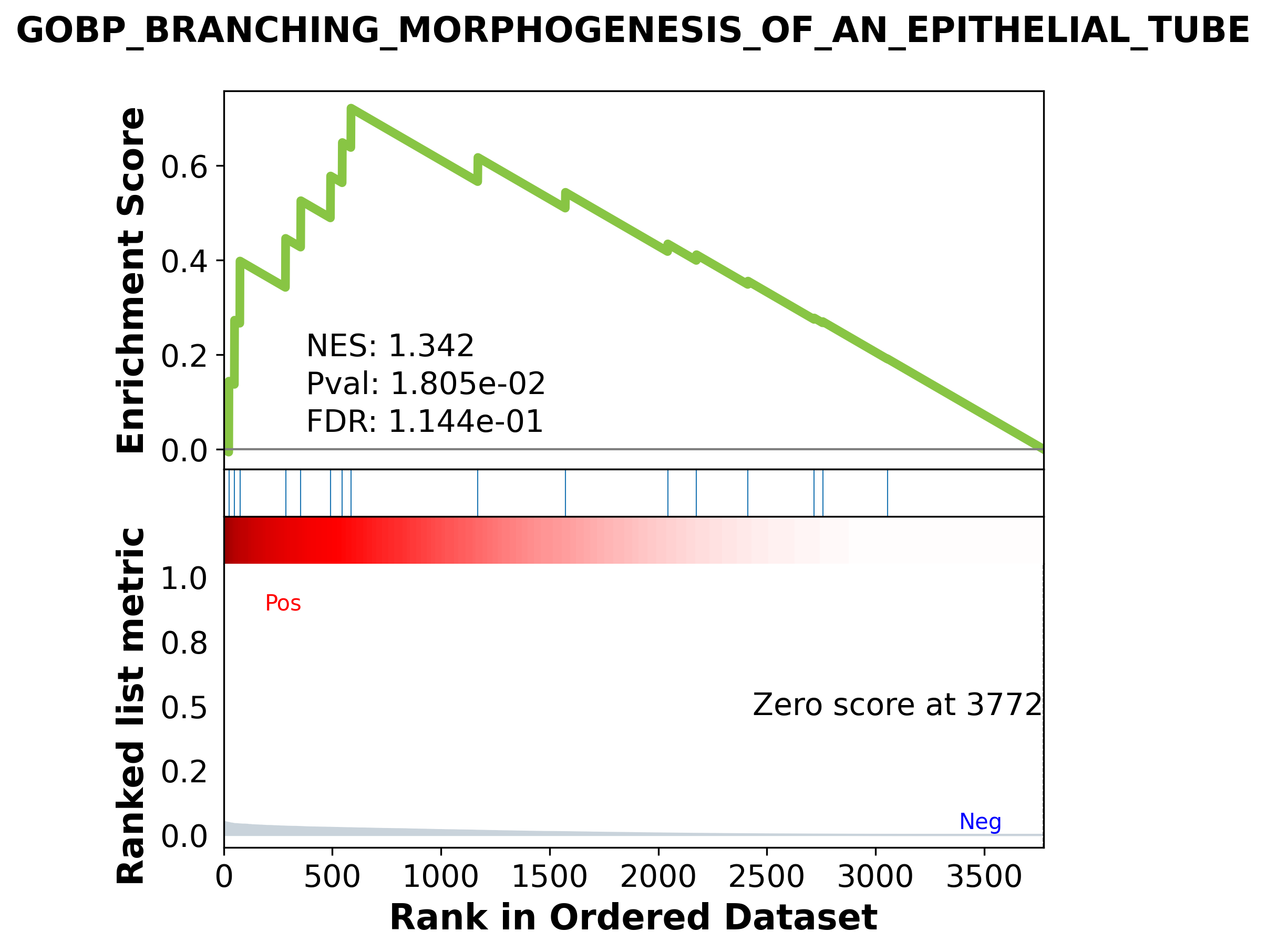

Supplement: Supplemental GSEA [file jciinsight-8-173374-s056.zip › GSEA/Factor 3/prerank/GOBP_BRANCHING_MORPHOGENESIS_OF_AN_EPITHELIAL_TUBE.png]

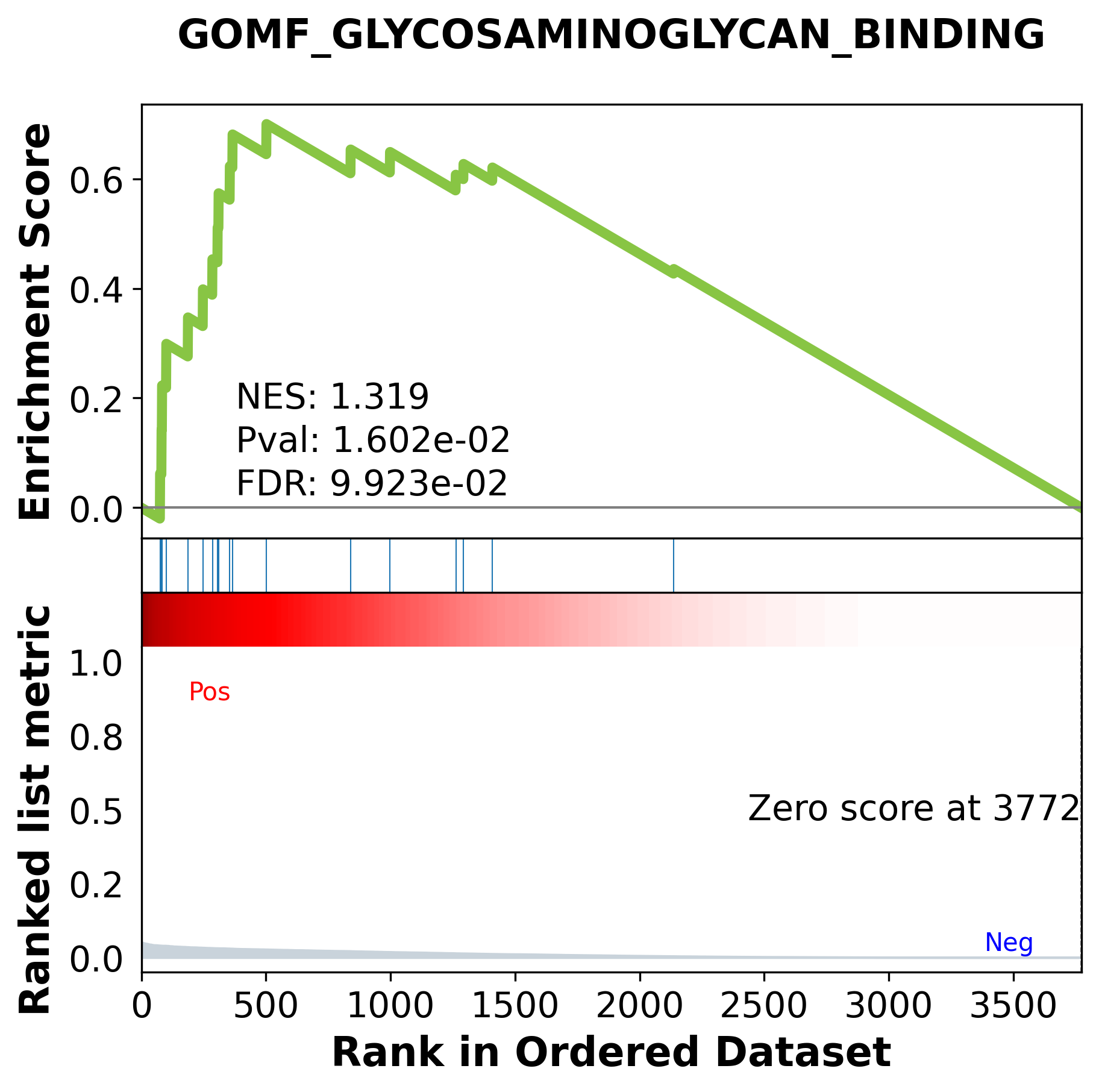

Supplement: Supplemental GSEA [file jciinsight-8-173374-s056.zip › GSEA/Factor 3/prerank/GOMF_GLYCOSAMINOGLYCAN_BINDING.png]

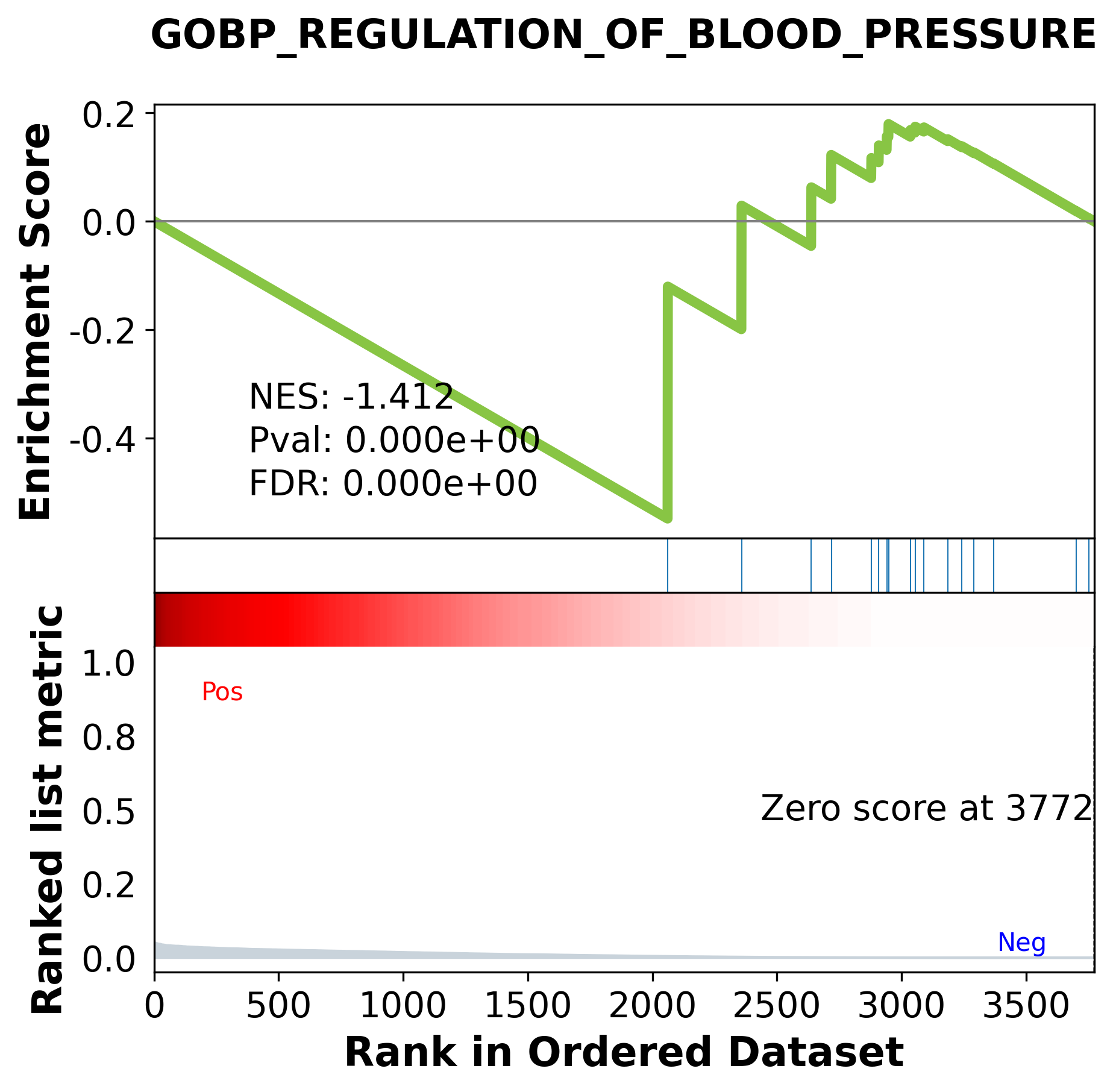

Supplement: Supplemental GSEA [file jciinsight-8-173374-s056.zip › GSEA/Factor 3/prerank/GOBP_REGULATION_OF_BLOOD_PRESSURE.png]

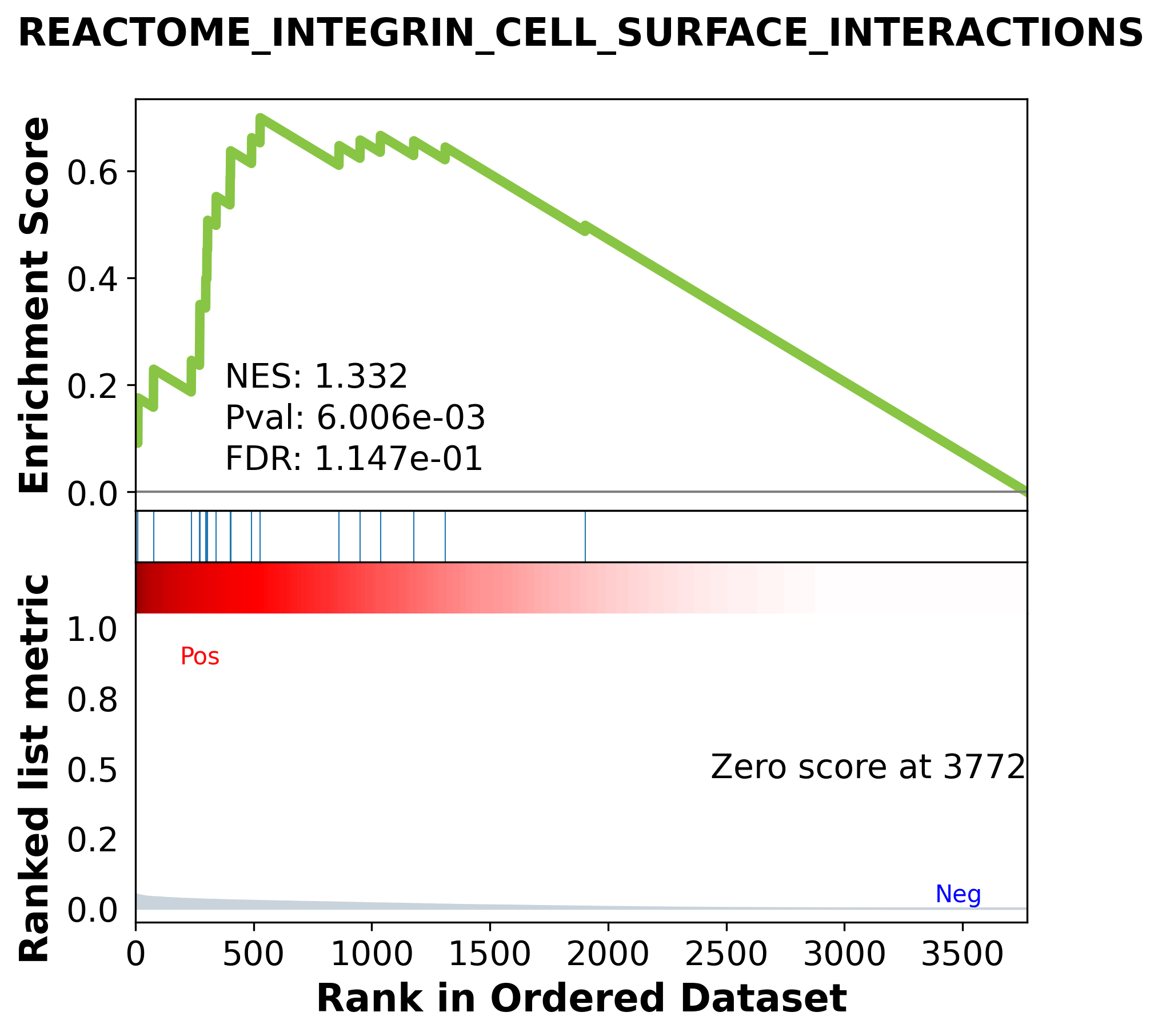

Supplement: Supplemental GSEA [file jciinsight-8-173374-s056.zip › GSEA/Factor 3/prerank/REACTOME_INTEGRIN_CELL_SURFACE_INTERACTIONS.png]

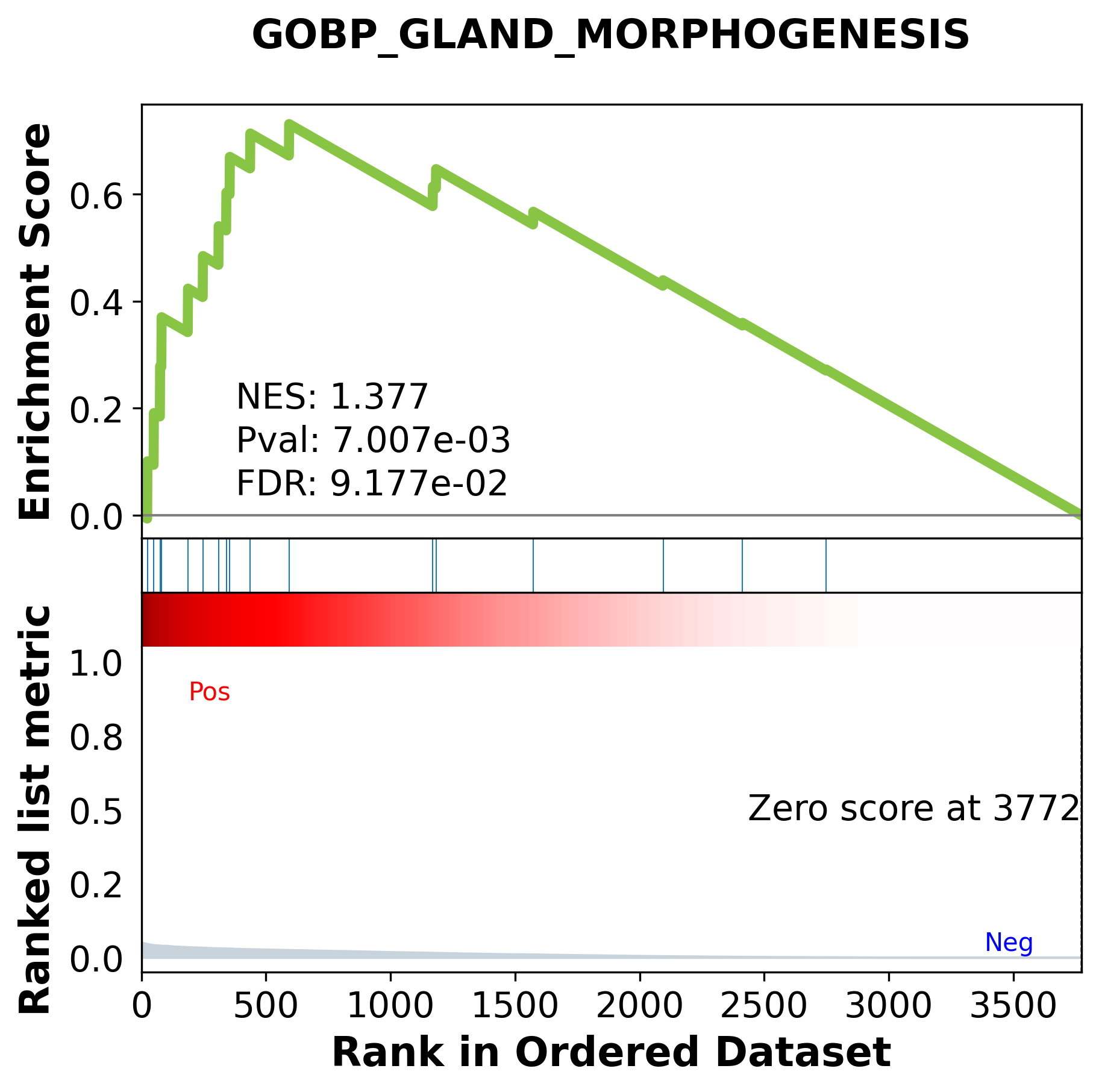

Supplement: Supplemental GSEA [file jciinsight-8-173374-s056.zip › GSEA/Factor 3/prerank/GOBP_GLAND_MORPHOGENESIS.png]

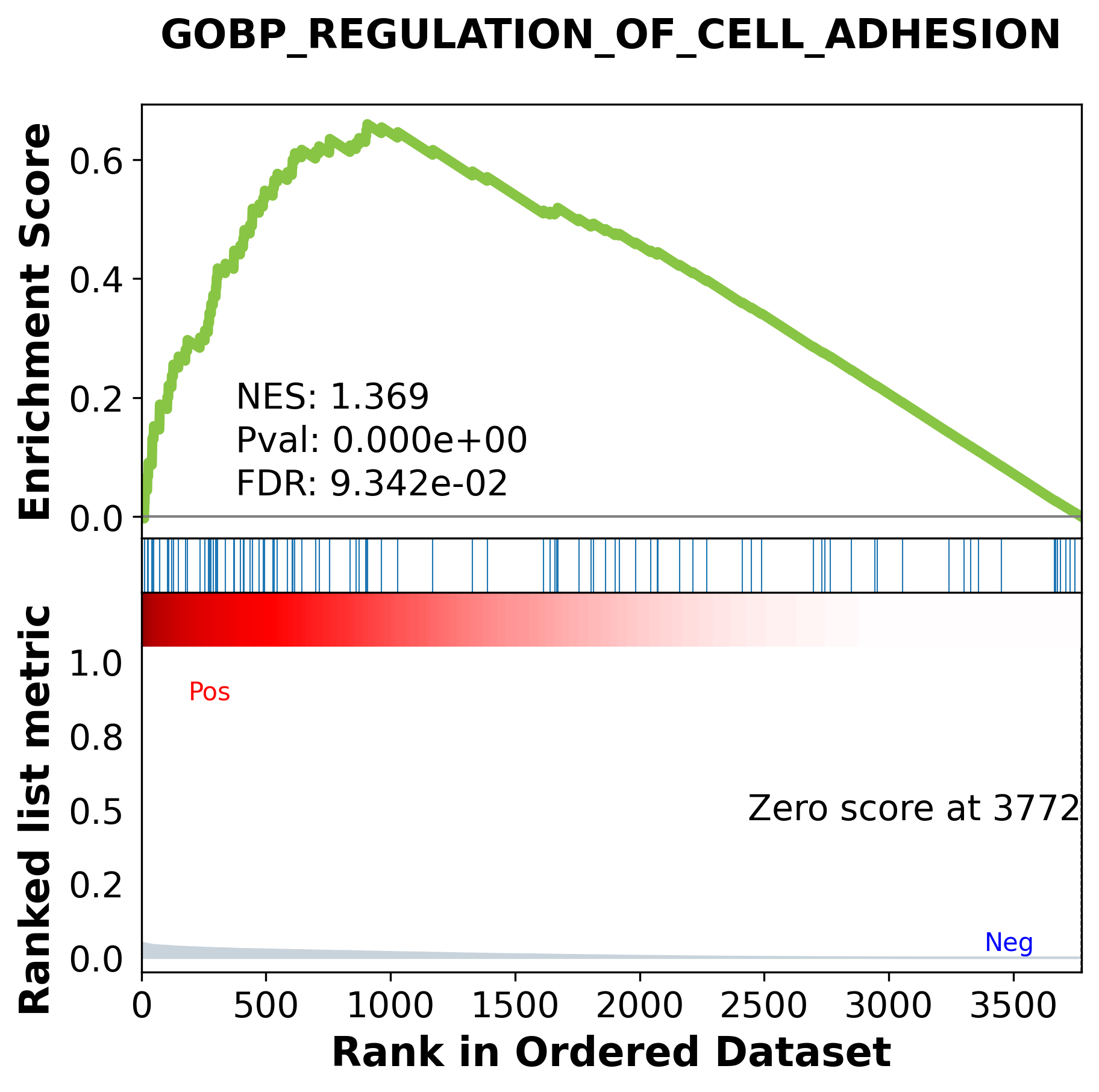

Supplement: Supplemental GSEA [file jciinsight-8-173374-s056.zip › GSEA/Factor 3/prerank/GOBP_REGULATION_OF_CELL_ADHESION.png]

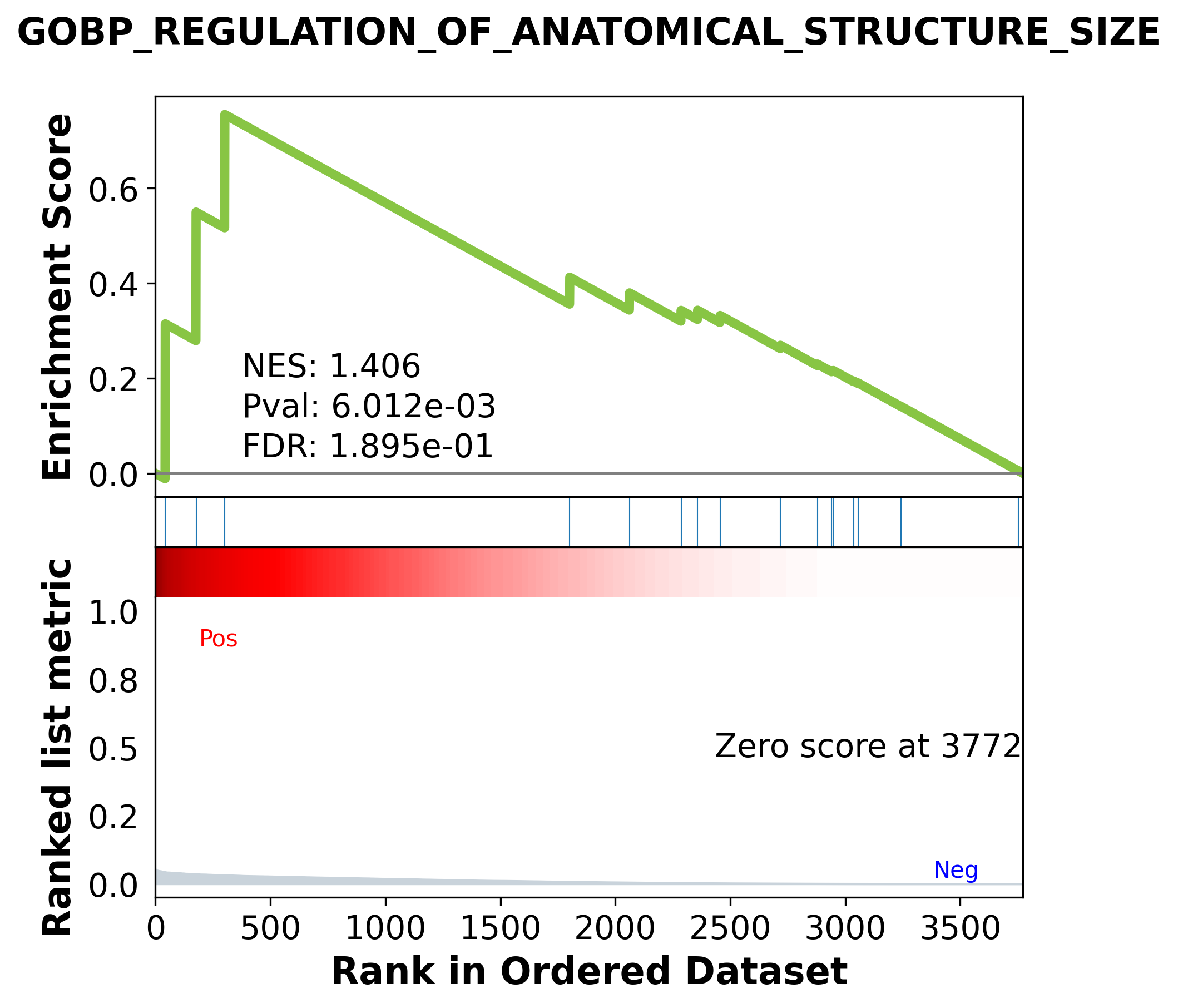

Supplement: Supplemental GSEA [file jciinsight-8-173374-s056.zip › GSEA/Factor 3/prerank/GOBP_REGULATION_OF_ANATOMICAL_STRUCTURE_SIZE.png]

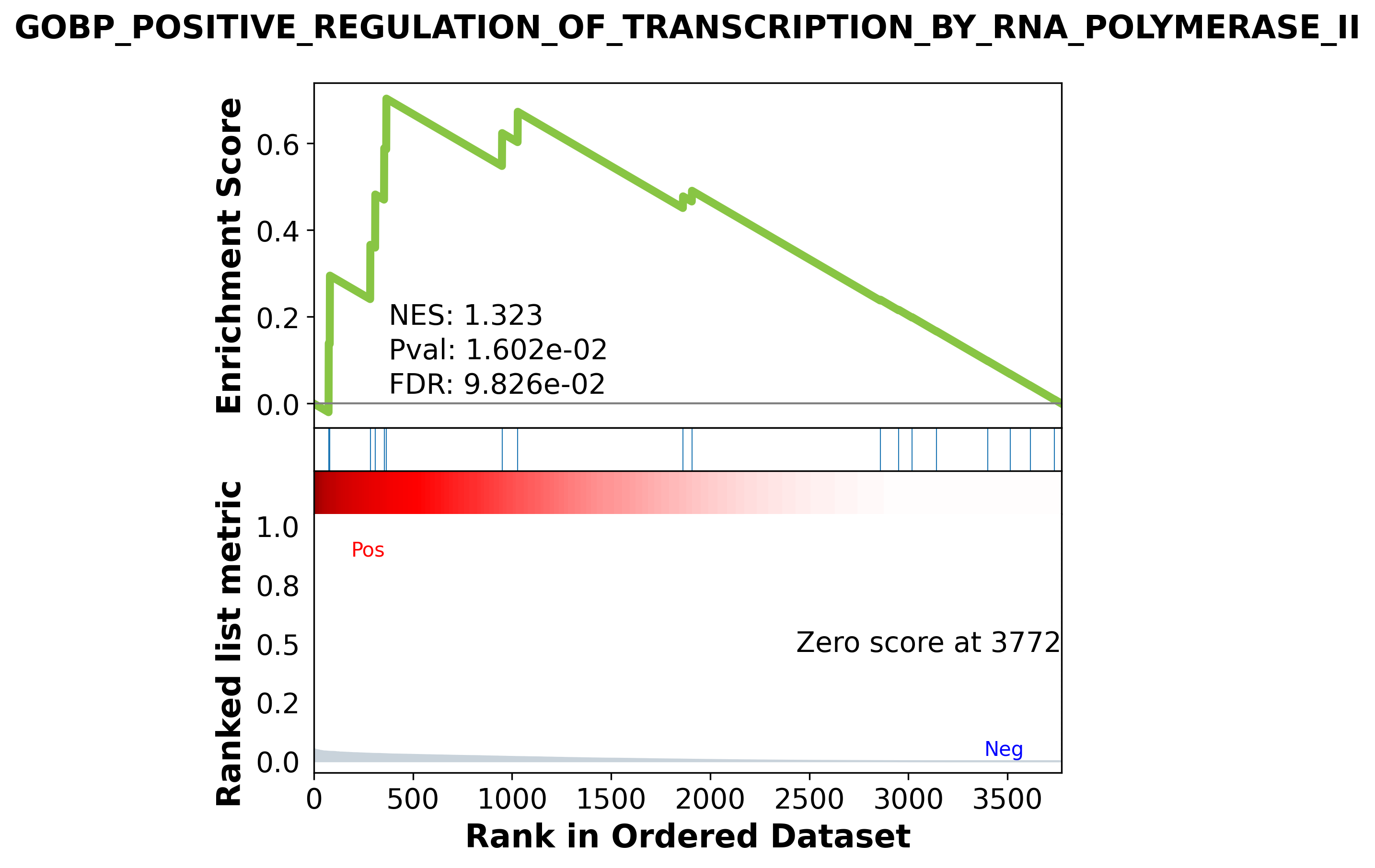

Supplement: Supplemental GSEA [file jciinsight-8-173374-s056.zip › GSEA/Factor 3/prerank/GOBP_POSITIVE_REGULATION_OF_TRANSCRIPTION_BY_RNA_POLYMERASE_II.png]

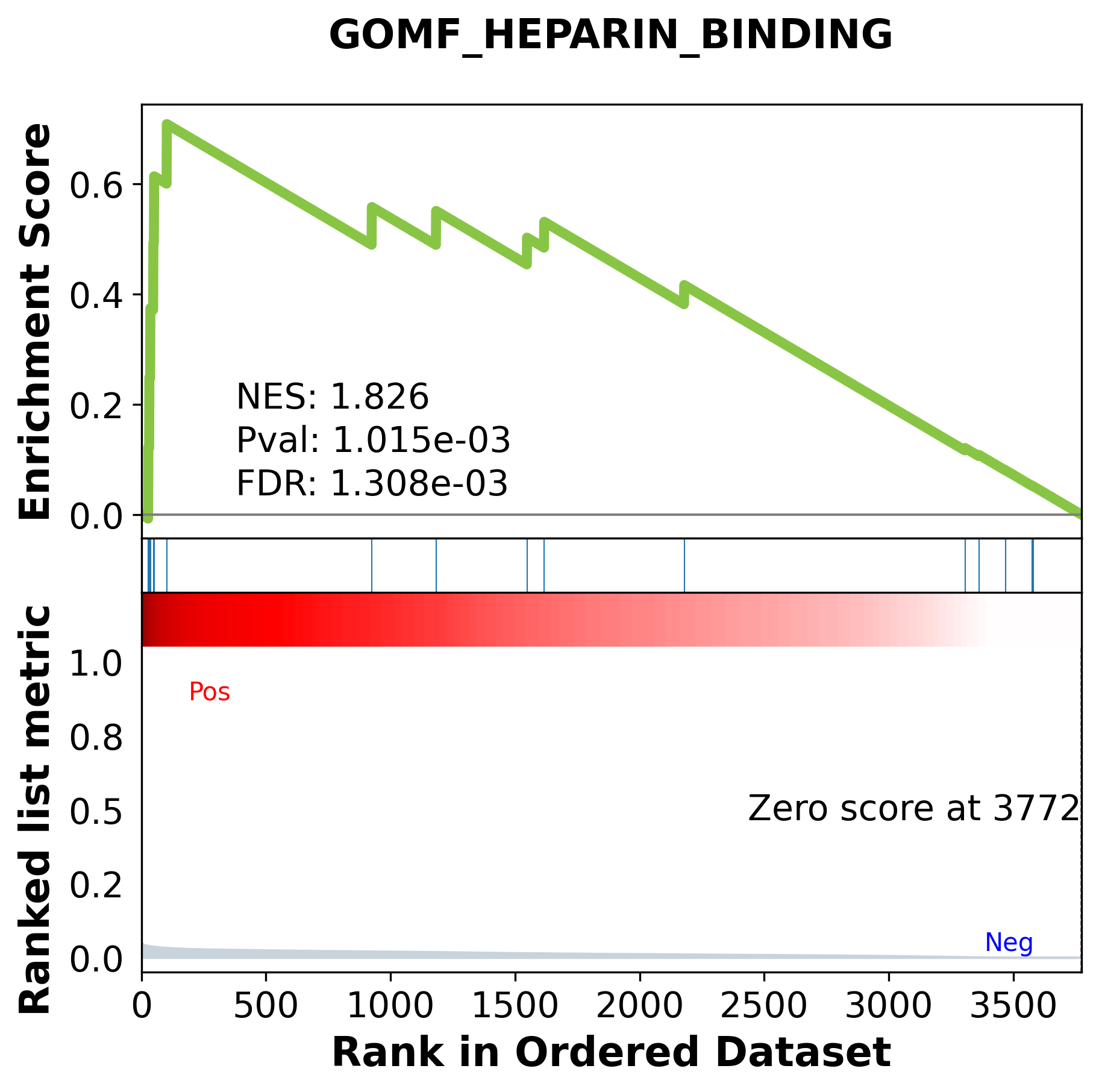

Supplement: Supplemental GSEA [file jciinsight-8-173374-s056.zip › GSEA/Factor 4/prerank/GOMF_HEPARIN_BINDING.png]

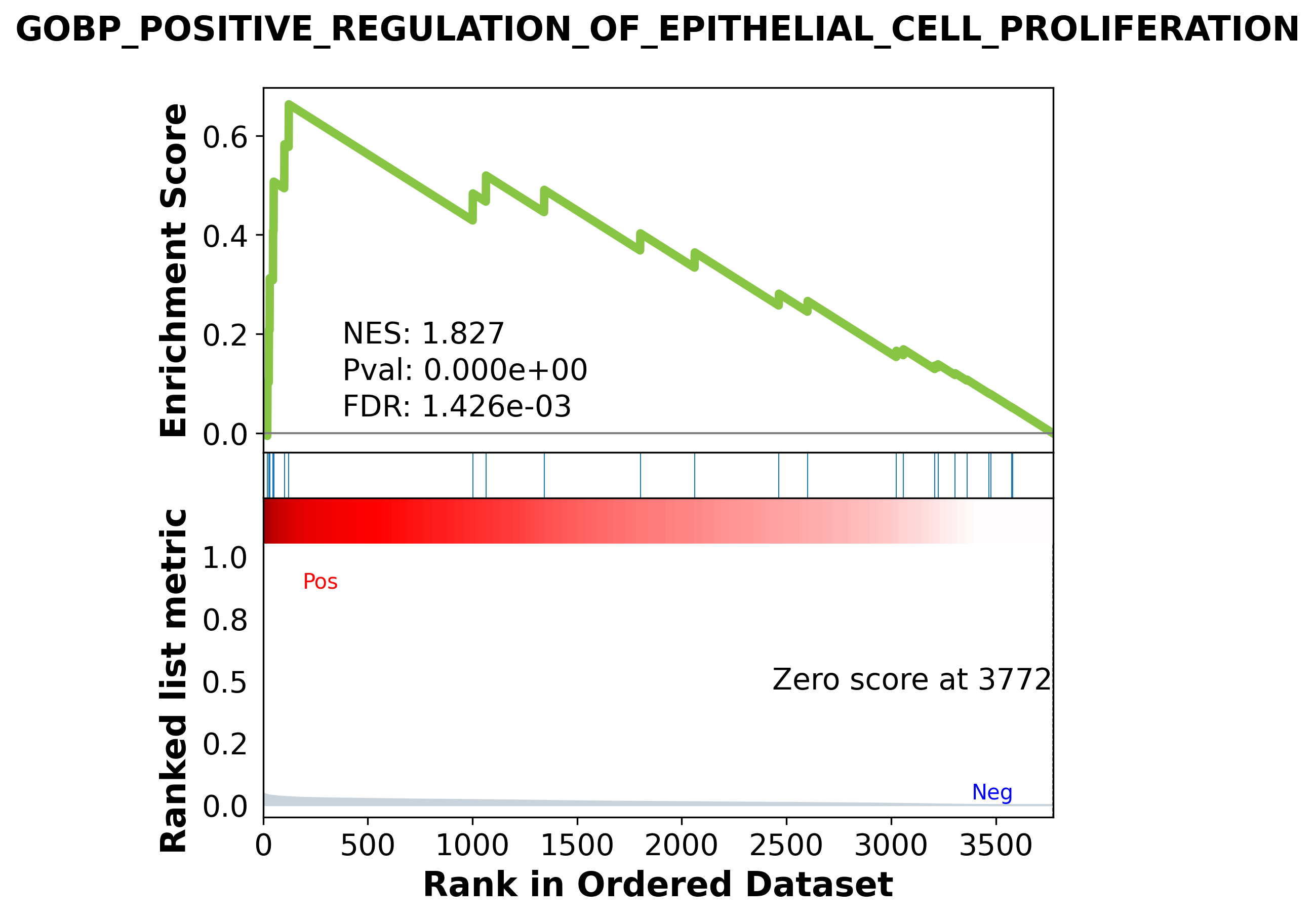

Supplement: Supplemental GSEA [file jciinsight-8-173374-s056.zip › GSEA/Factor 4/prerank/GOBP_POSITIVE_REGULATION_OF_EPITHELIAL_CELL_PROLIFERATION.png]

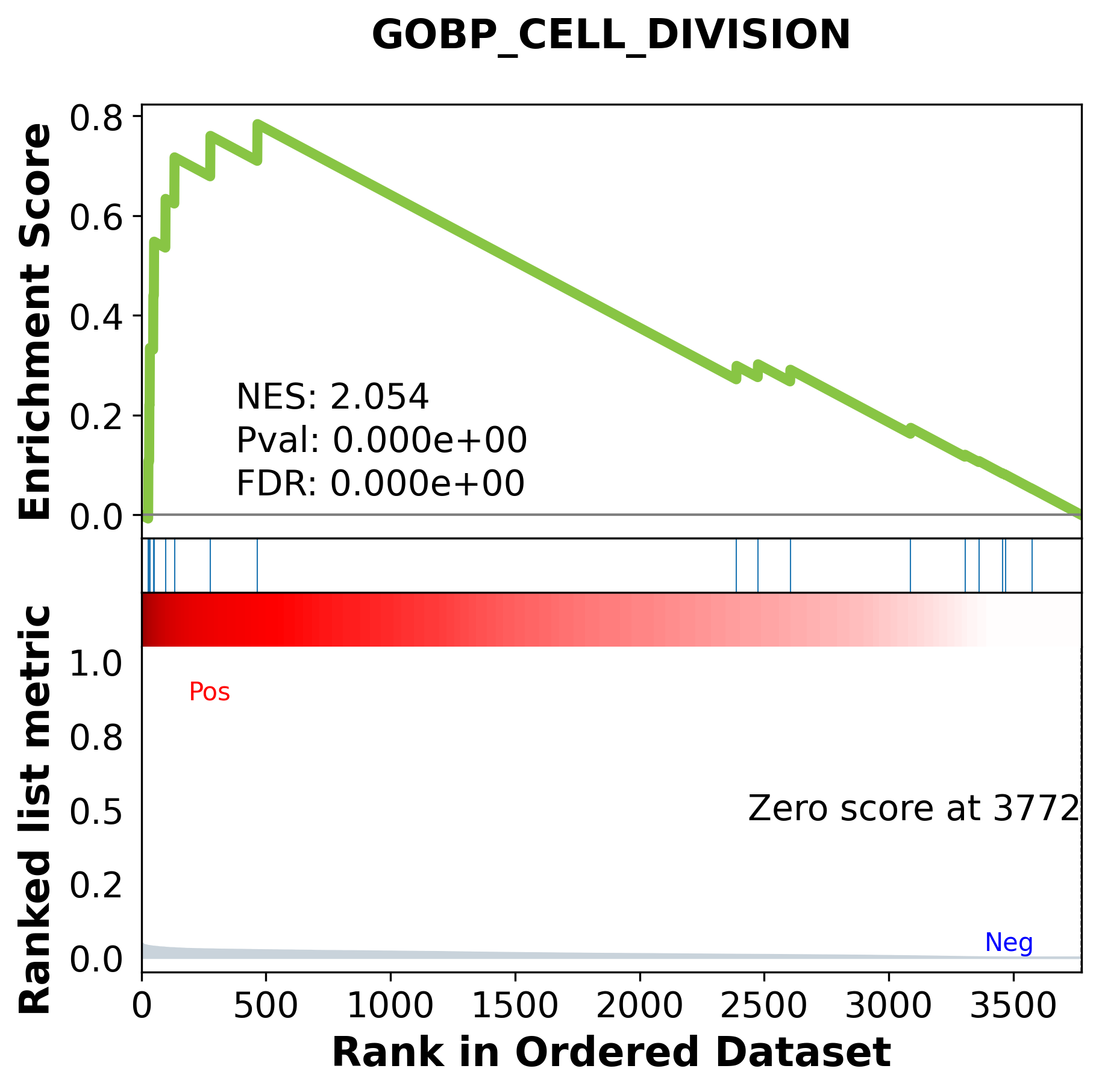

Supplement: Supplemental GSEA [file jciinsight-8-173374-s056.zip › GSEA/Factor 4/prerank/GOBP_CELL_DIVISION.png]

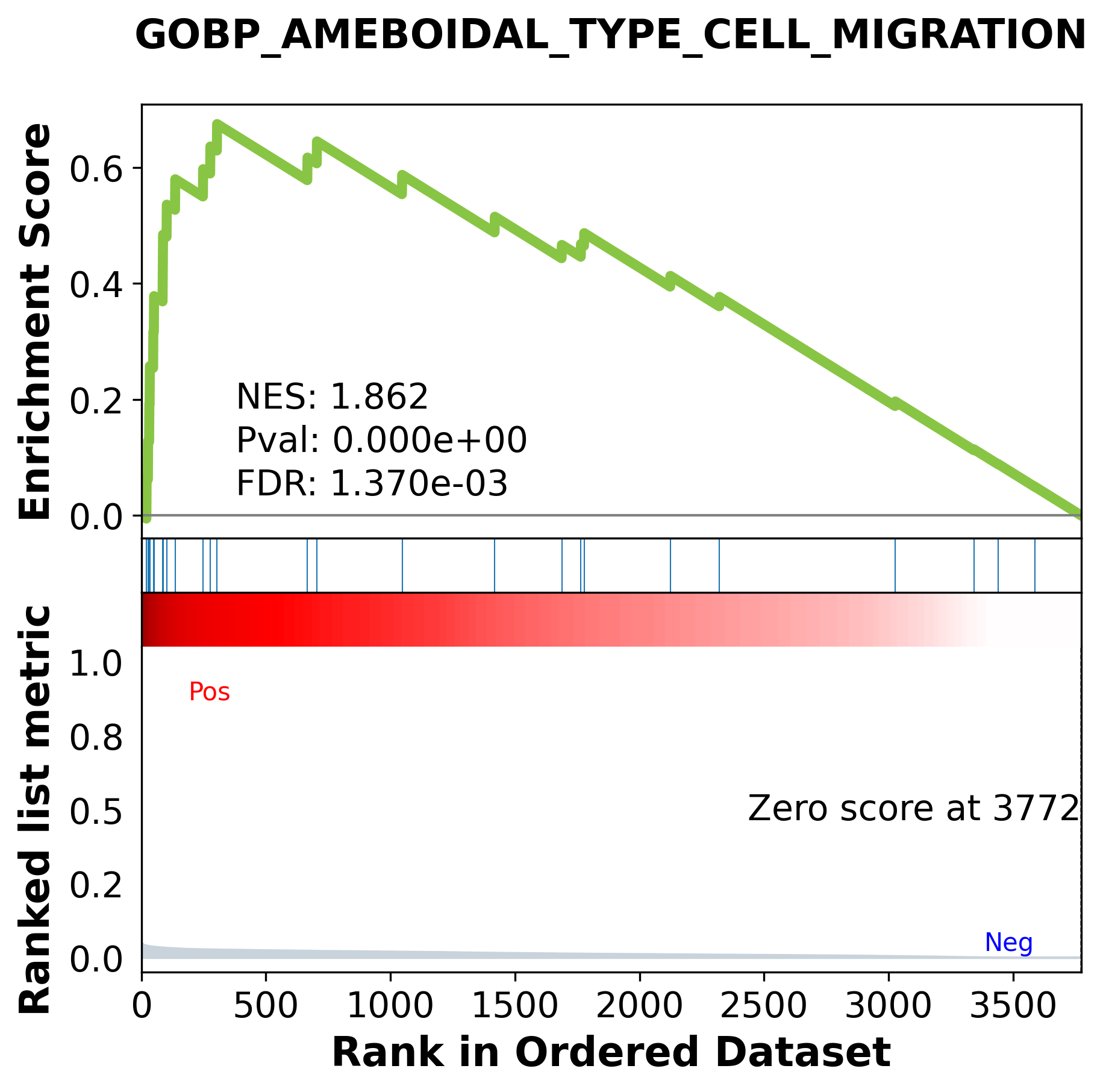

Supplement: Supplemental GSEA [file jciinsight-8-173374-s056.zip › GSEA/Factor 4/prerank/GOBP_AMEBOIDAL_TYPE_CELL_MIGRATION.png]

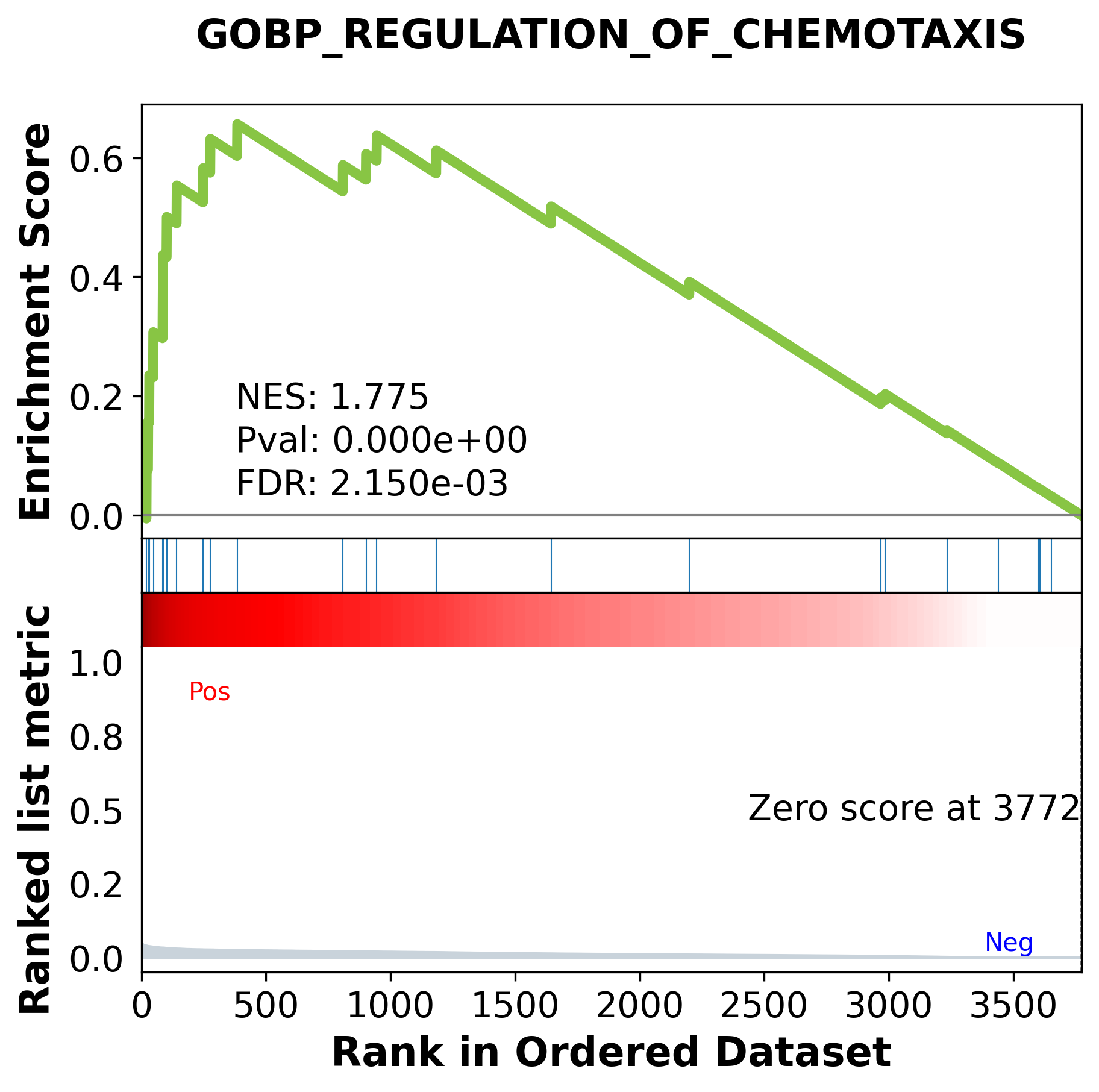

Supplement: Supplemental GSEA [file jciinsight-8-173374-s056.zip › GSEA/Factor 4/prerank/GOBP_REGULATION_OF_CHEMOTAXIS.png]

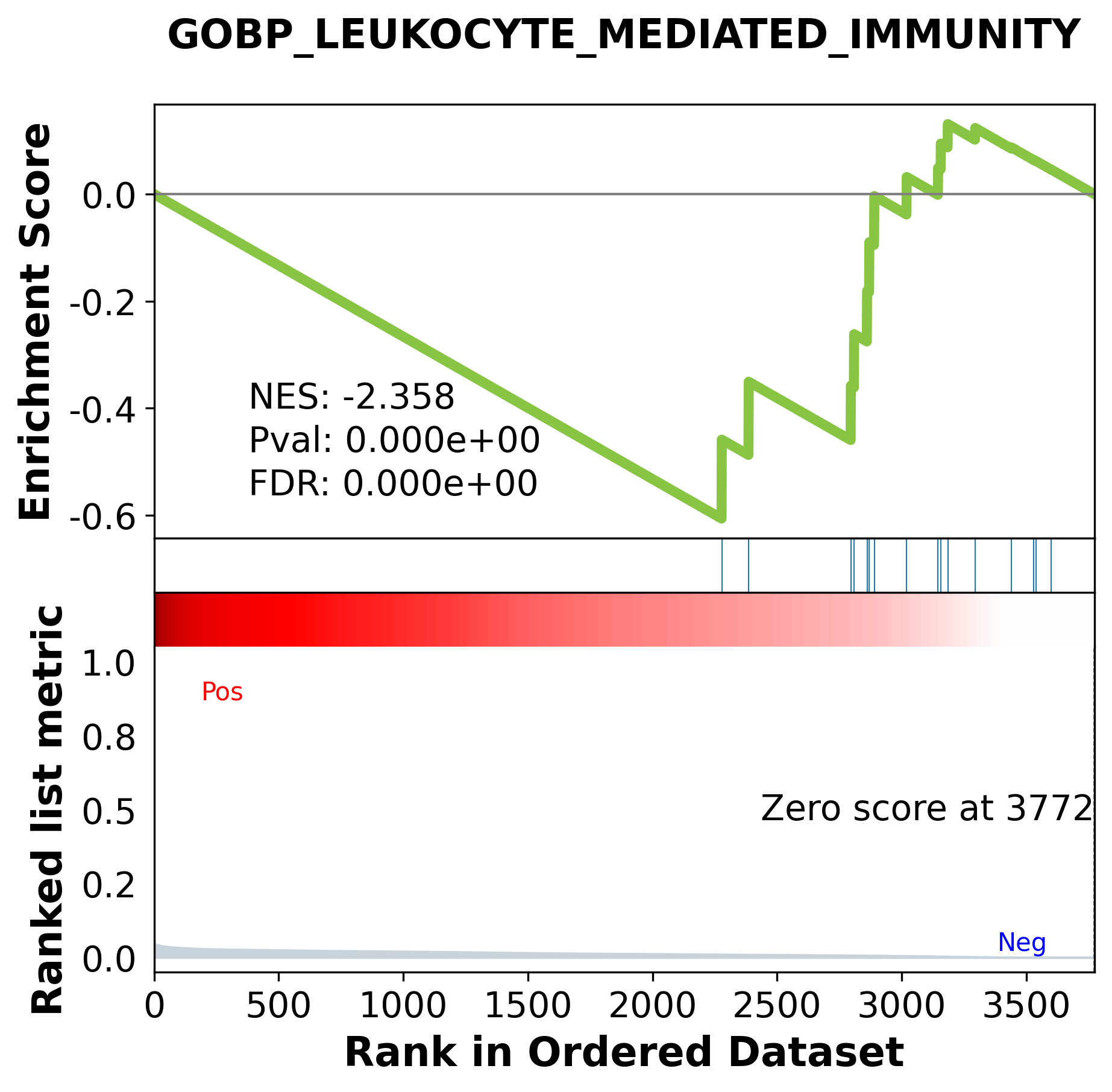

Supplement: Supplemental GSEA [file jciinsight-8-173374-s056.zip › GSEA/Factor 4/prerank/GOBP_LEUKOCYTE_MEDIATED_IMMUNITY.png]

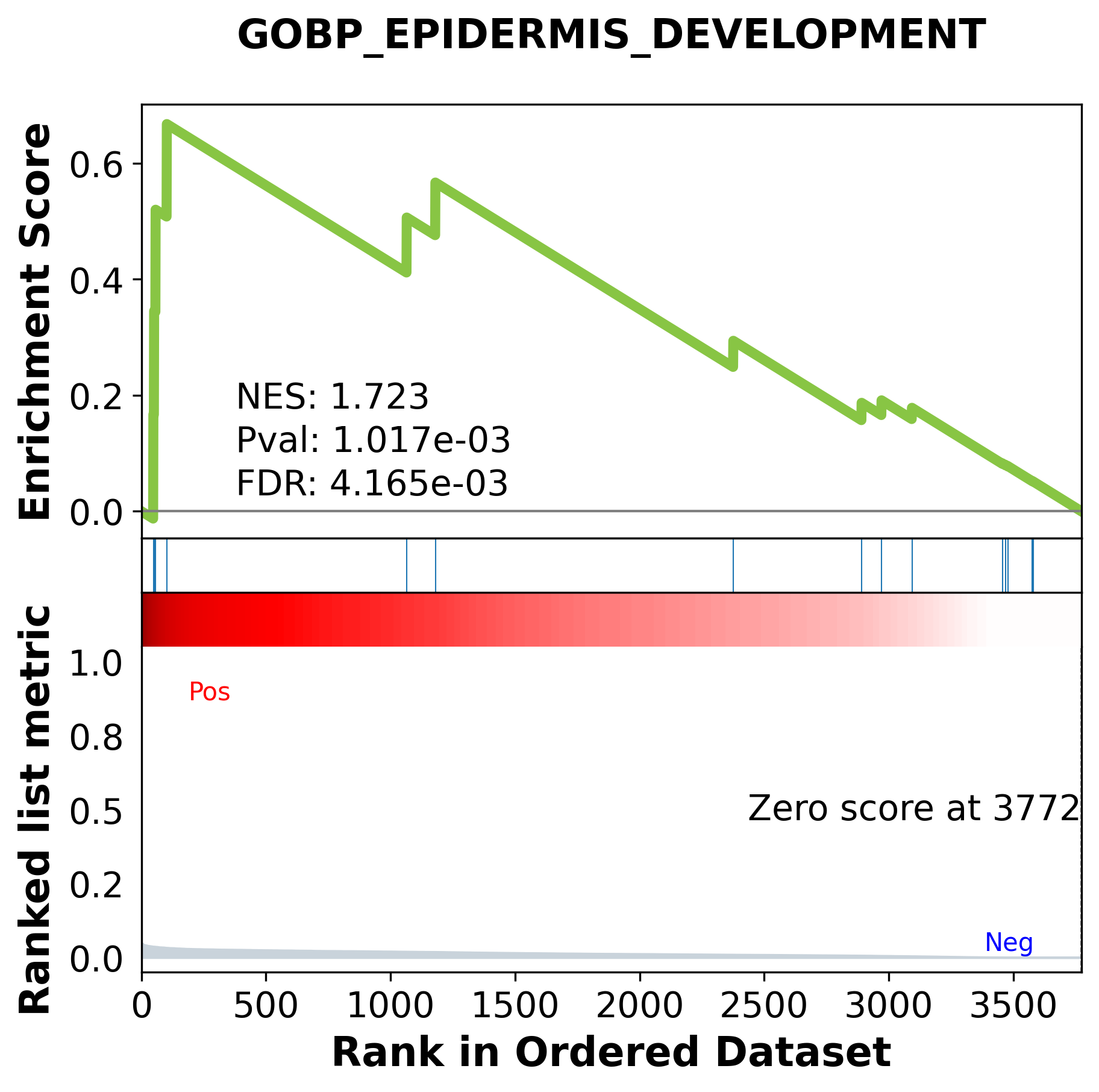

Supplement: Supplemental GSEA [file jciinsight-8-173374-s056.zip › GSEA/Factor 4/prerank/GOBP_EPIDERMIS_DEVELOPMENT.png]

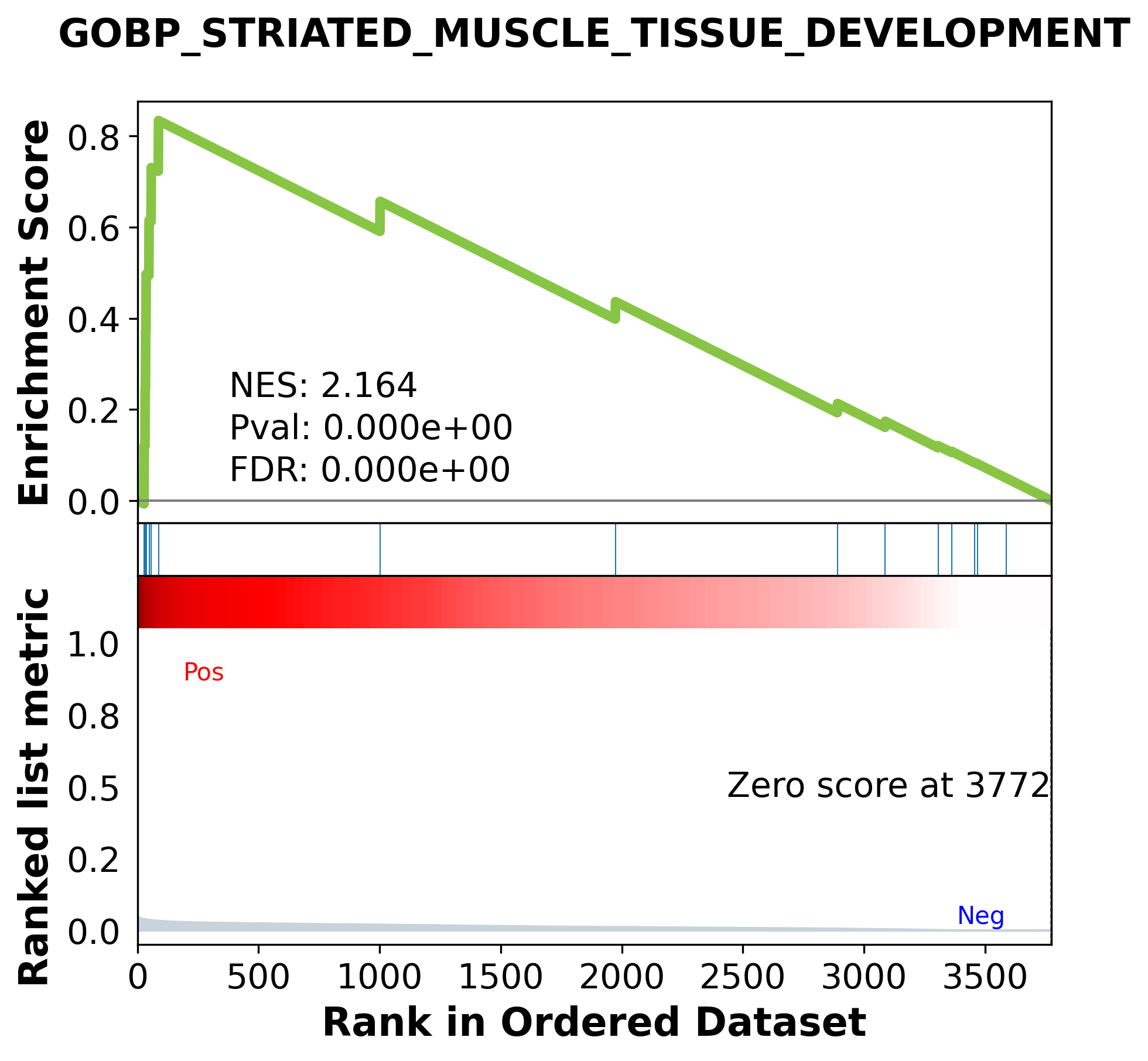

Supplement: Supplemental GSEA [file jciinsight-8-173374-s056.zip › GSEA/Factor 4/prerank/GOBP_STRIATED_MUSCLE_TISSUE_DEVELOPMENT.png]

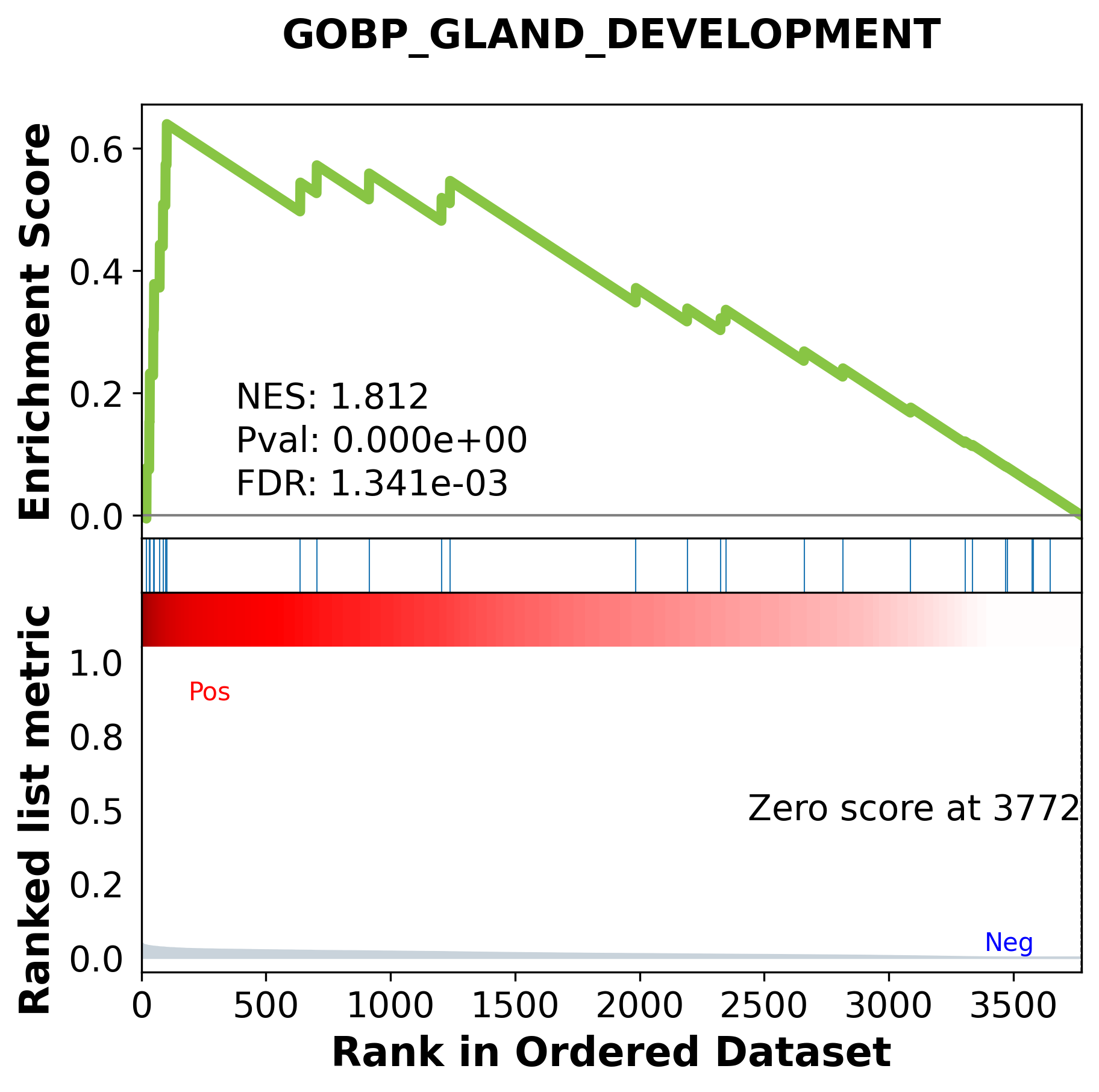

Supplement: Supplemental GSEA [file jciinsight-8-173374-s056.zip › GSEA/Factor 4/prerank/GOBP_GLAND_DEVELOPMENT.png]

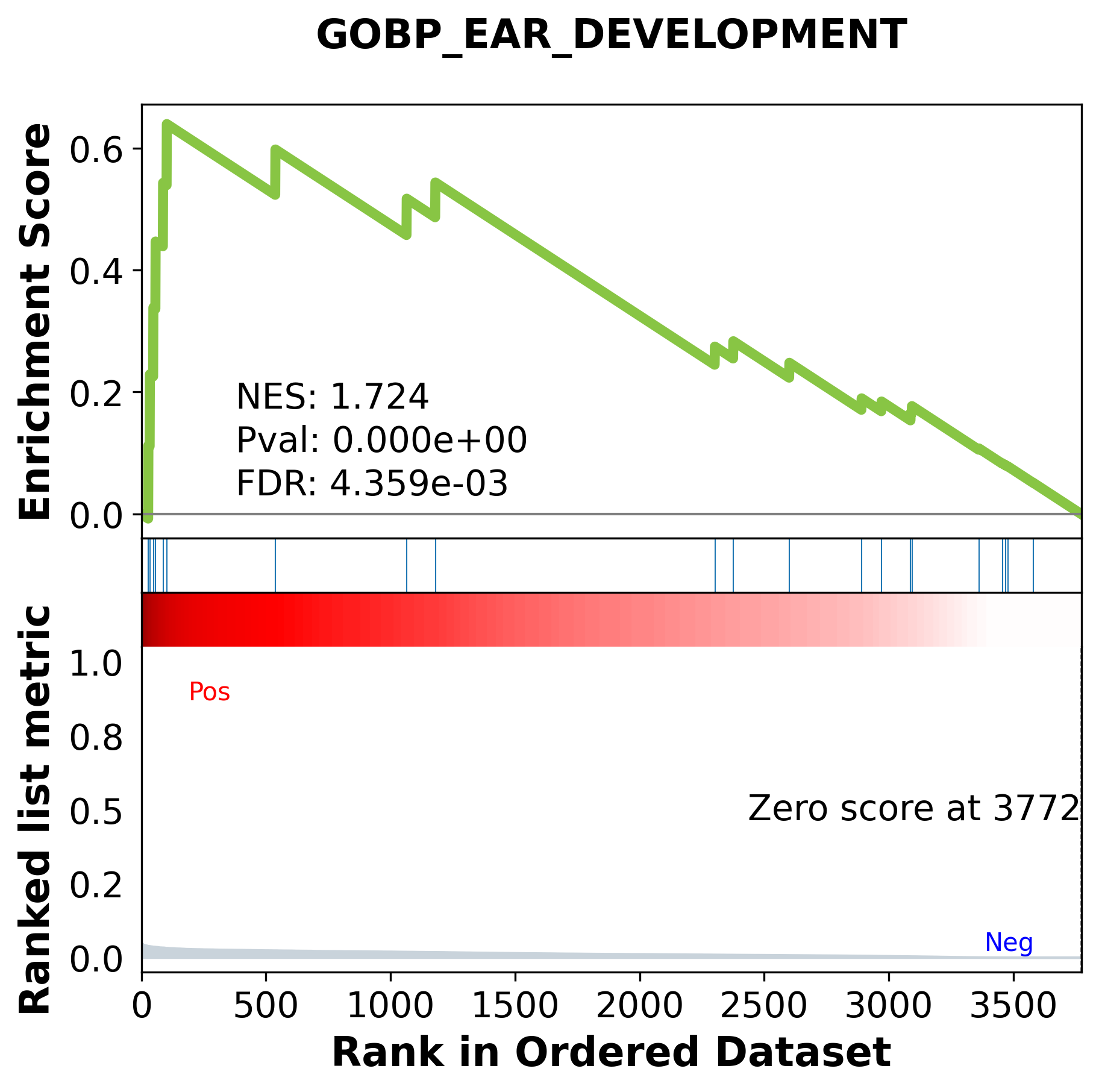

Supplement: Supplemental GSEA [file jciinsight-8-173374-s056.zip › GSEA/Factor 4/prerank/GOBP_EAR_DEVELOPMENT.png]

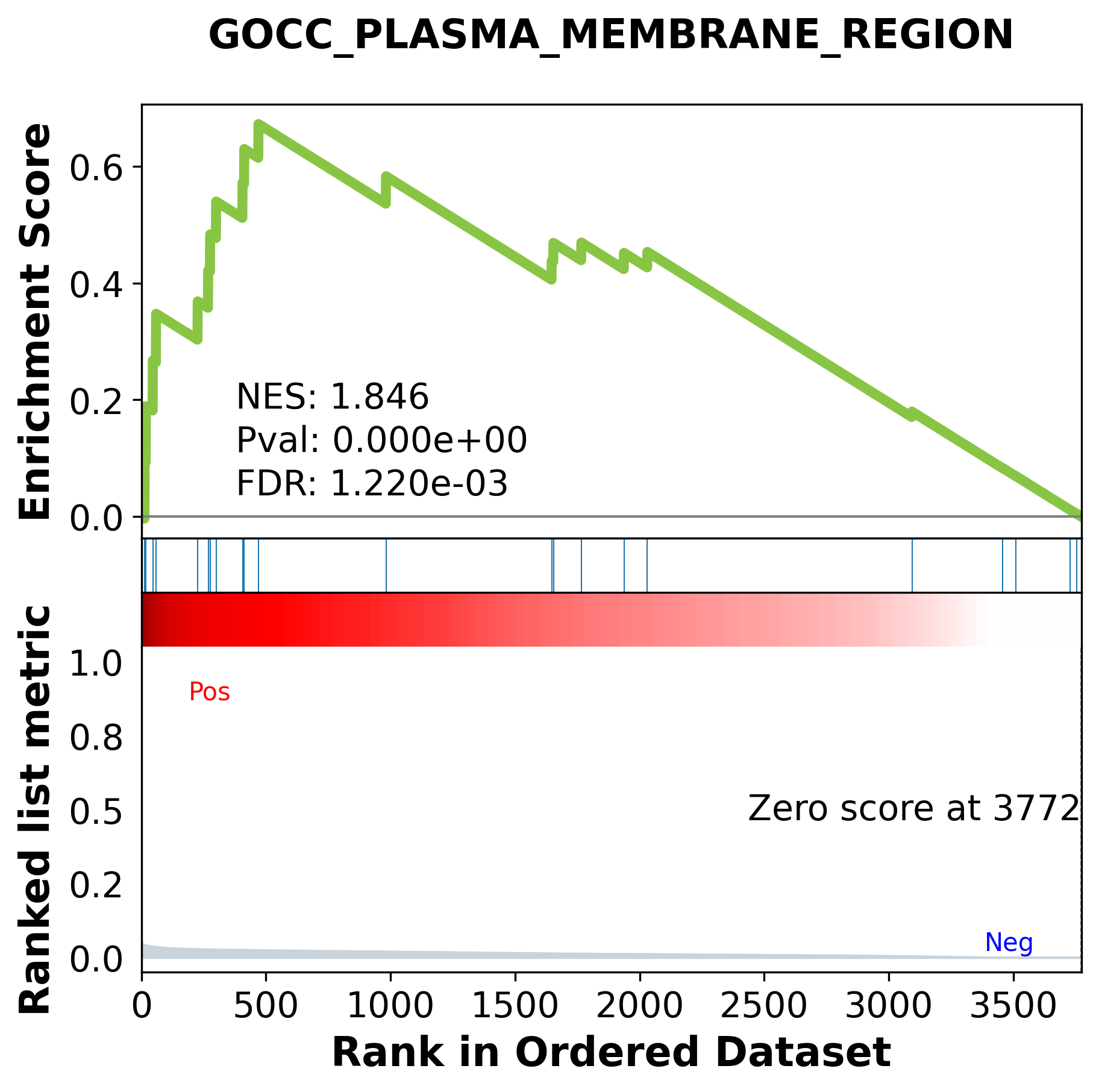

Supplement: Supplemental GSEA [file jciinsight-8-173374-s056.zip › GSEA/Factor 4/prerank/GOCC_PLASMA_MEMBRANE_REGION.png]

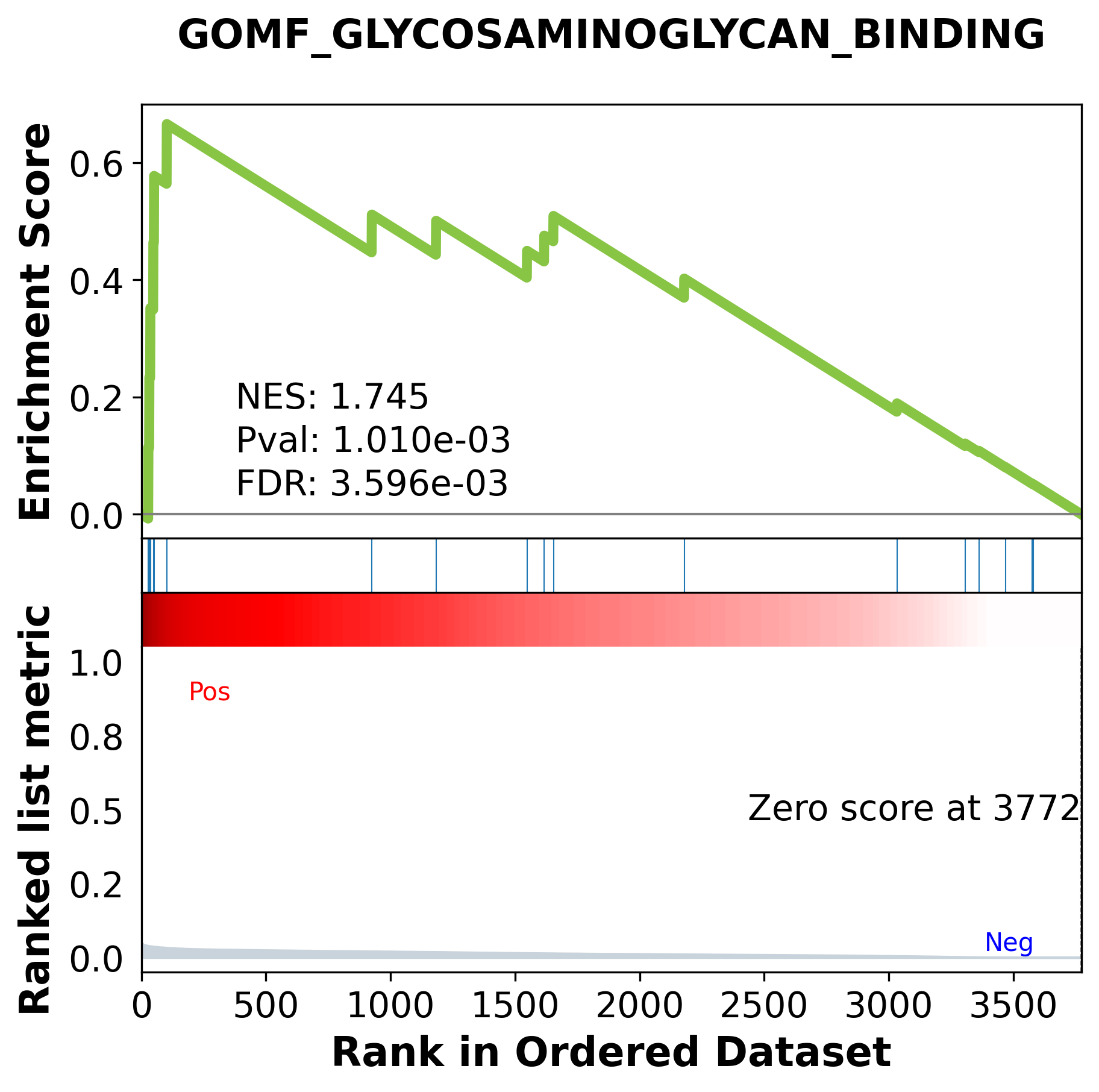

Supplement: Supplemental GSEA [file jciinsight-8-173374-s056.zip › GSEA/Factor 4/prerank/GOMF_GLYCOSAMINOGLYCAN_BINDING.png]

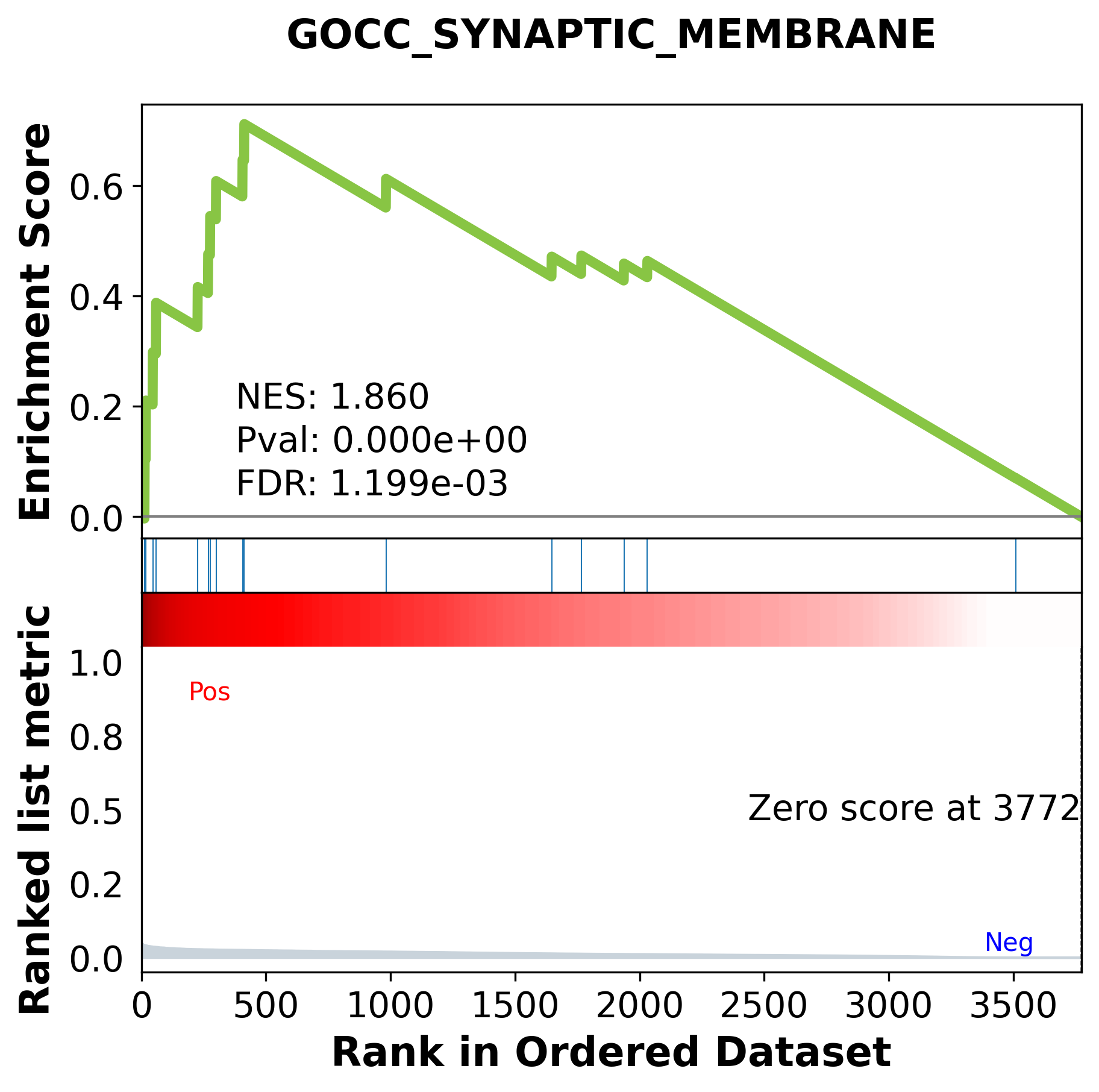

Supplement: Supplemental GSEA [file jciinsight-8-173374-s056.zip › GSEA/Factor 4/prerank/GOCC_SYNAPTIC_MEMBRANE.png]

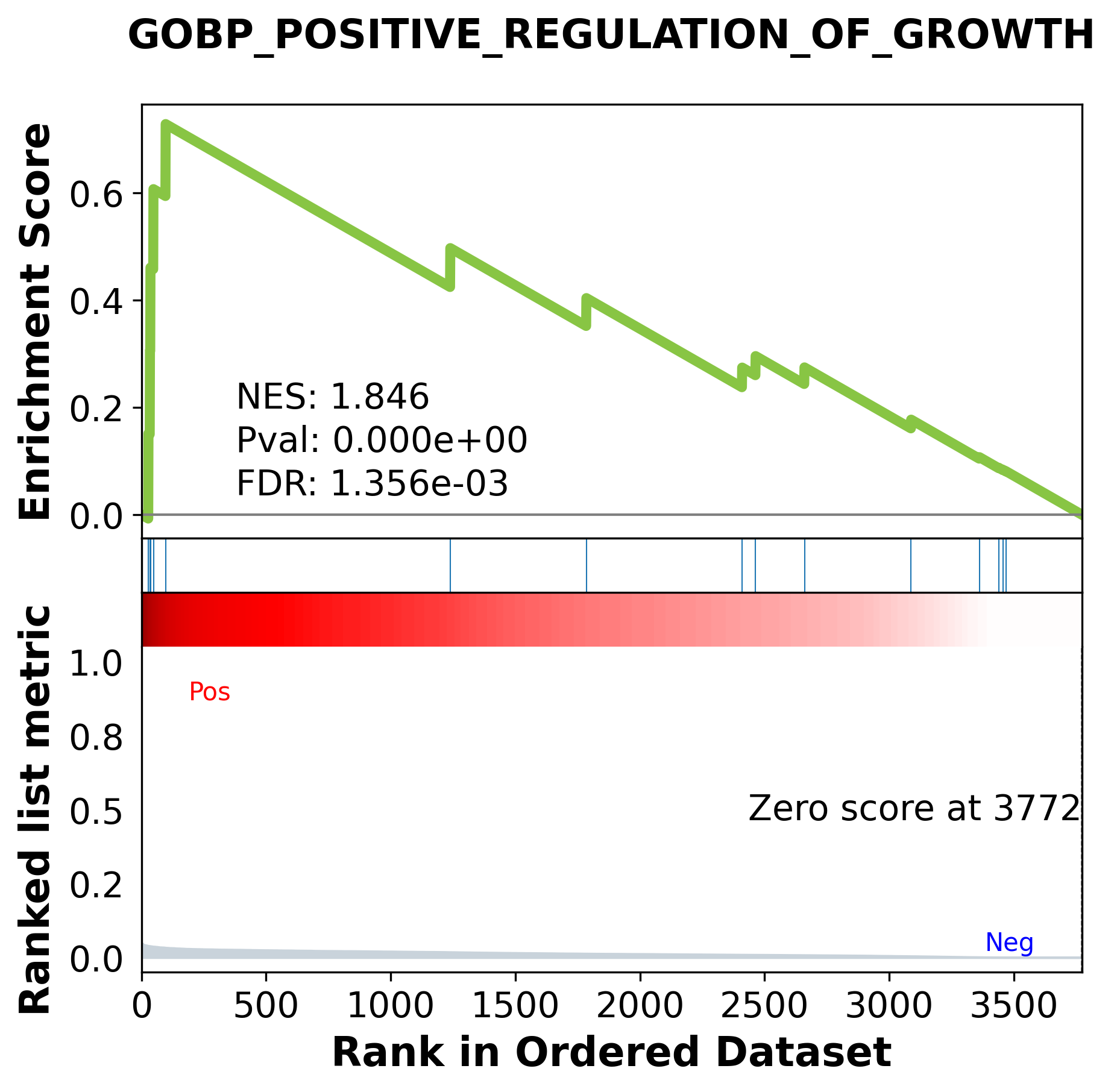

Supplement: Supplemental GSEA [file jciinsight-8-173374-s056.zip › GSEA/Factor 4/prerank/GOBP_POSITIVE_REGULATION_OF_GROWTH.png]

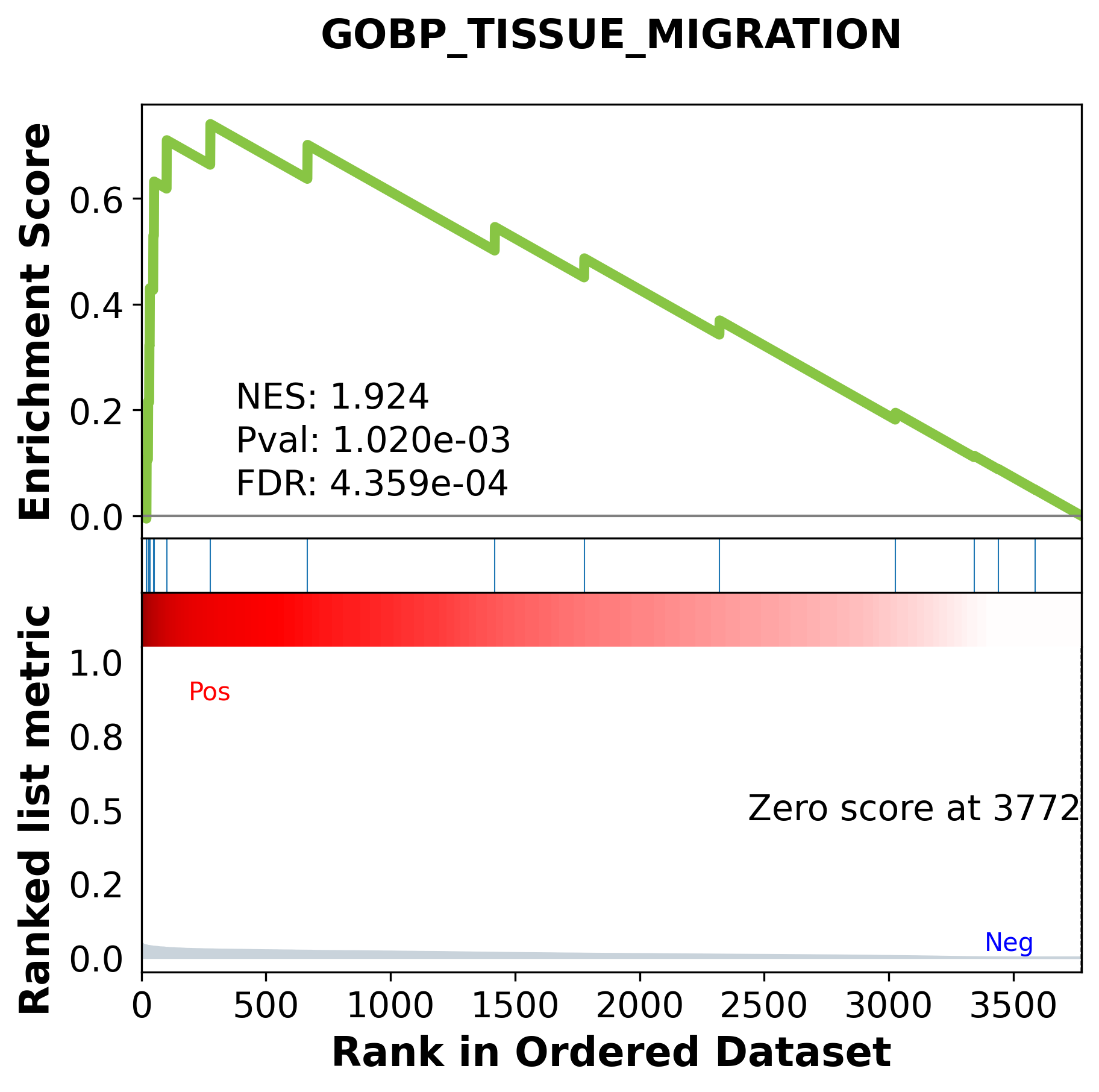

Supplement: Supplemental GSEA [file jciinsight-8-173374-s056.zip › GSEA/Factor 4/prerank/GOBP_TISSUE_MIGRATION.png]

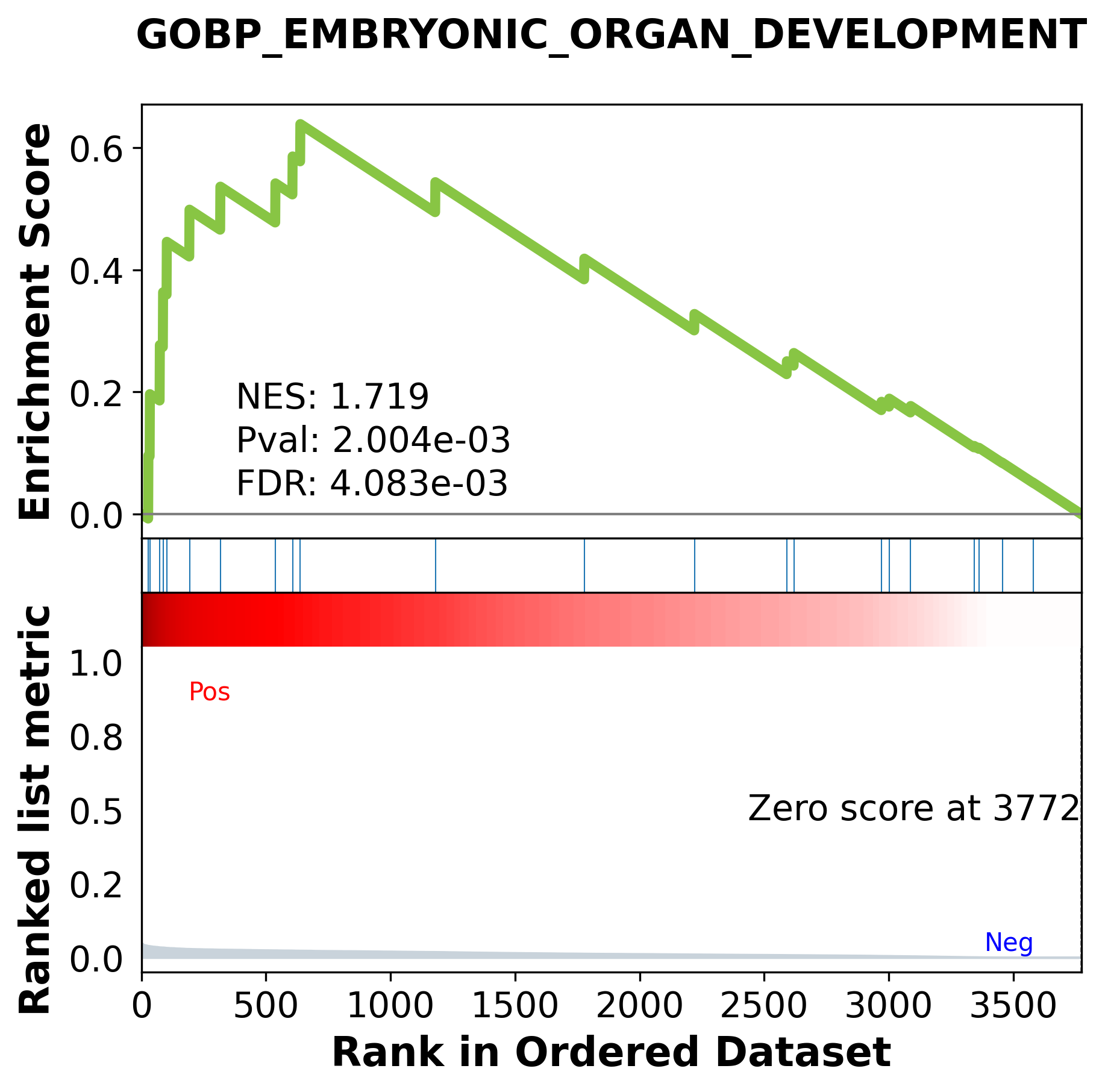

Supplement: Supplemental GSEA [file jciinsight-8-173374-s056.zip › GSEA/Factor 4/prerank/GOBP_EMBRYONIC_ORGAN_DEVELOPMENT.png]

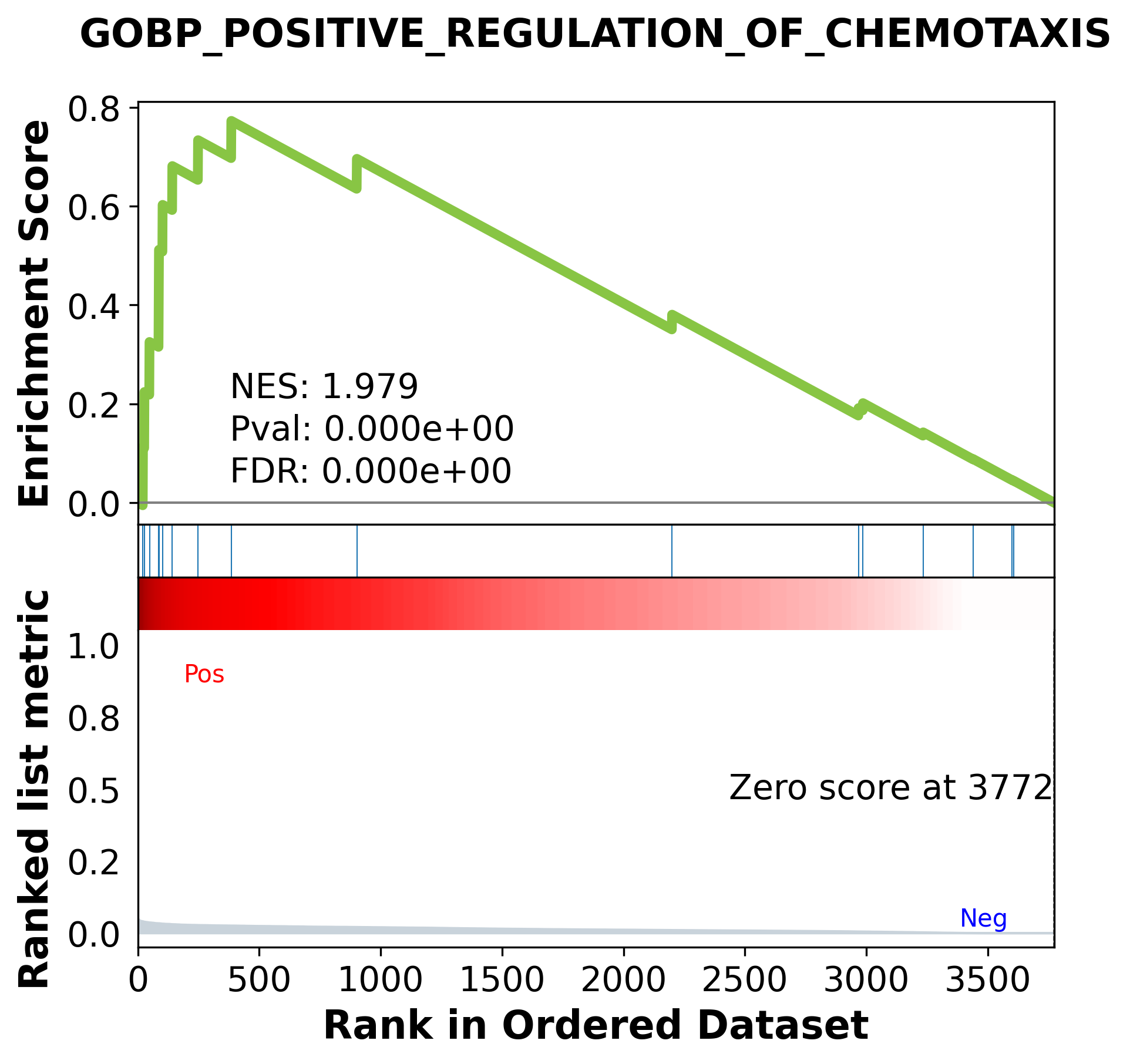

Supplement: Supplemental GSEA [file jciinsight-8-173374-s056.zip › GSEA/Factor 4/prerank/GOBP_POSITIVE_REGULATION_OF_CHEMOTAXIS.png]

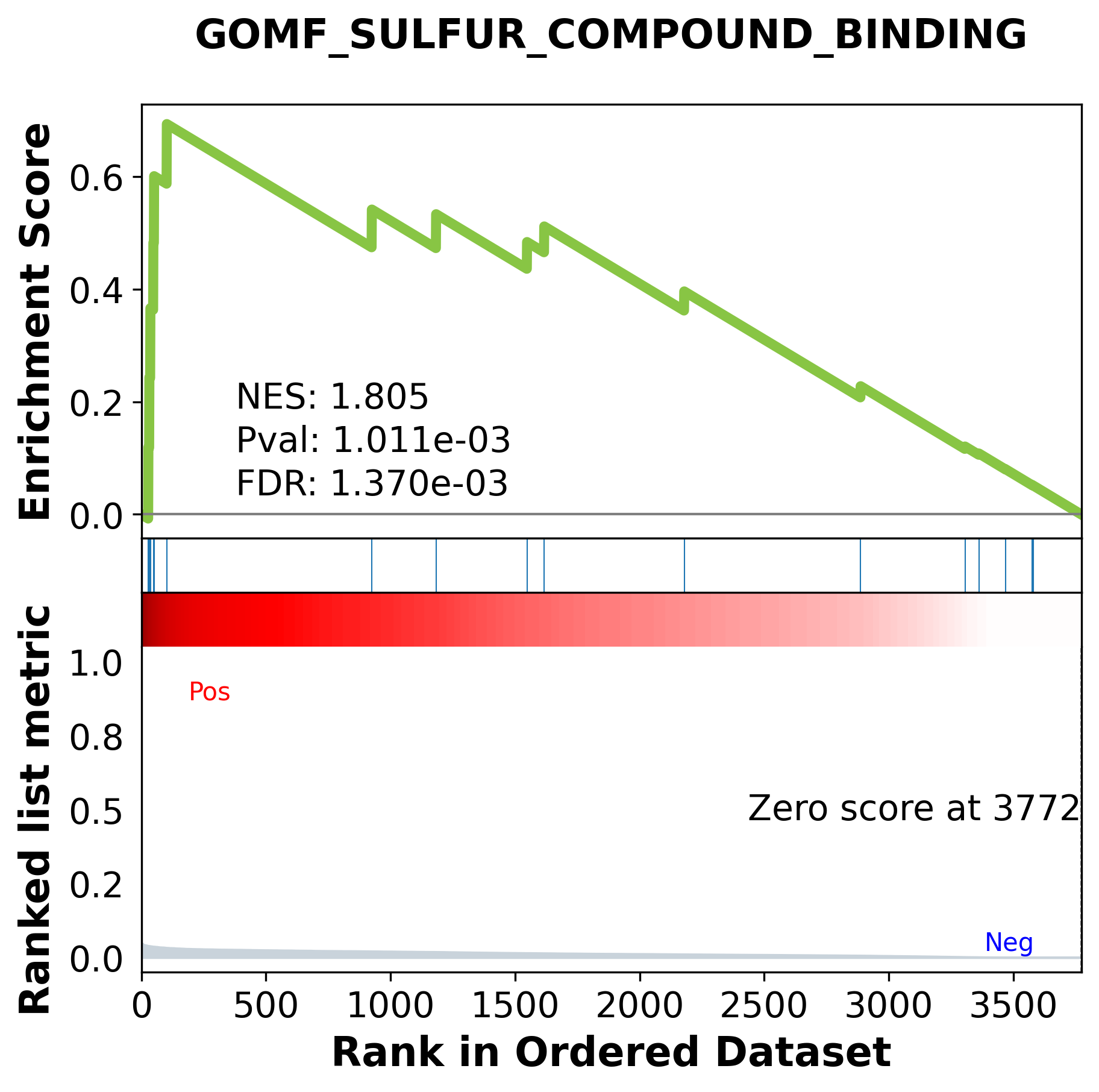

Supplement: Supplemental GSEA [file jciinsight-8-173374-s056.zip › GSEA/Factor 4/prerank/GOMF_SULFUR_COMPOUND_BINDING.png]

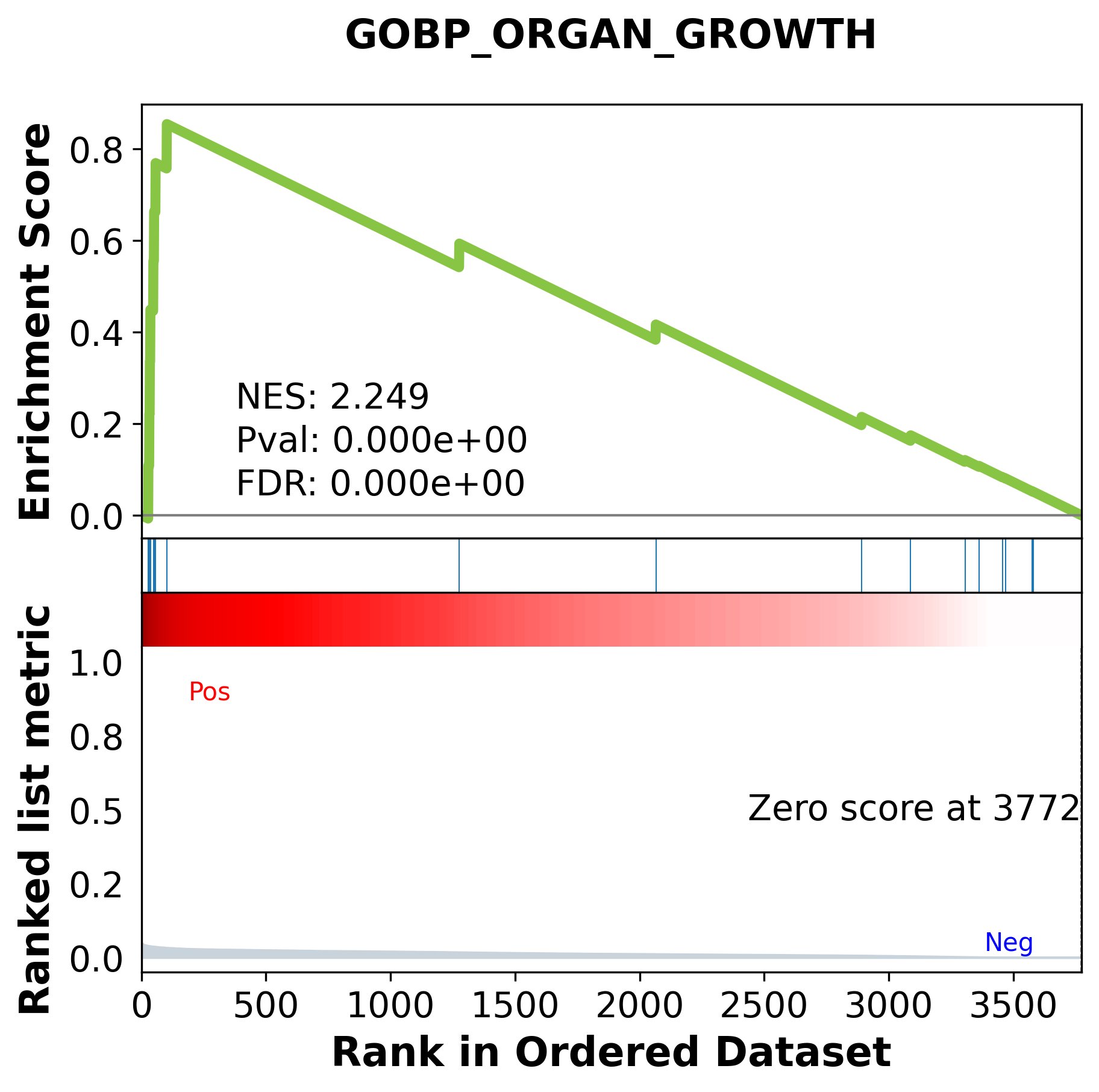

Supplement: Supplemental GSEA [file jciinsight-8-173374-s056.zip › GSEA/Factor 4/prerank/GOBP_ORGAN_GROWTH.png]

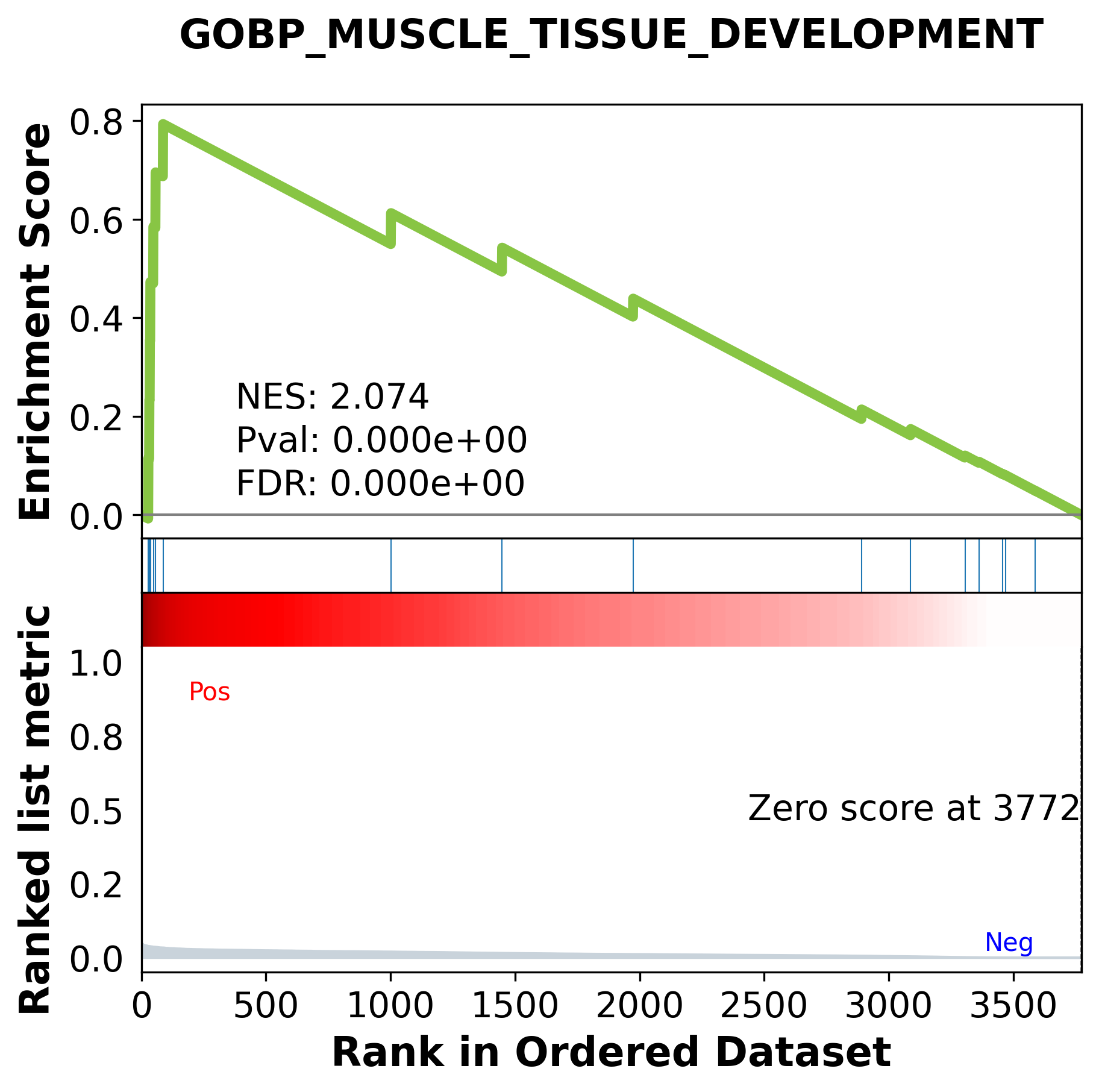

Supplement: Supplemental GSEA [file jciinsight-8-173374-s056.zip › GSEA/Factor 4/prerank/GOBP_MUSCLE_TISSUE_DEVELOPMENT.png]

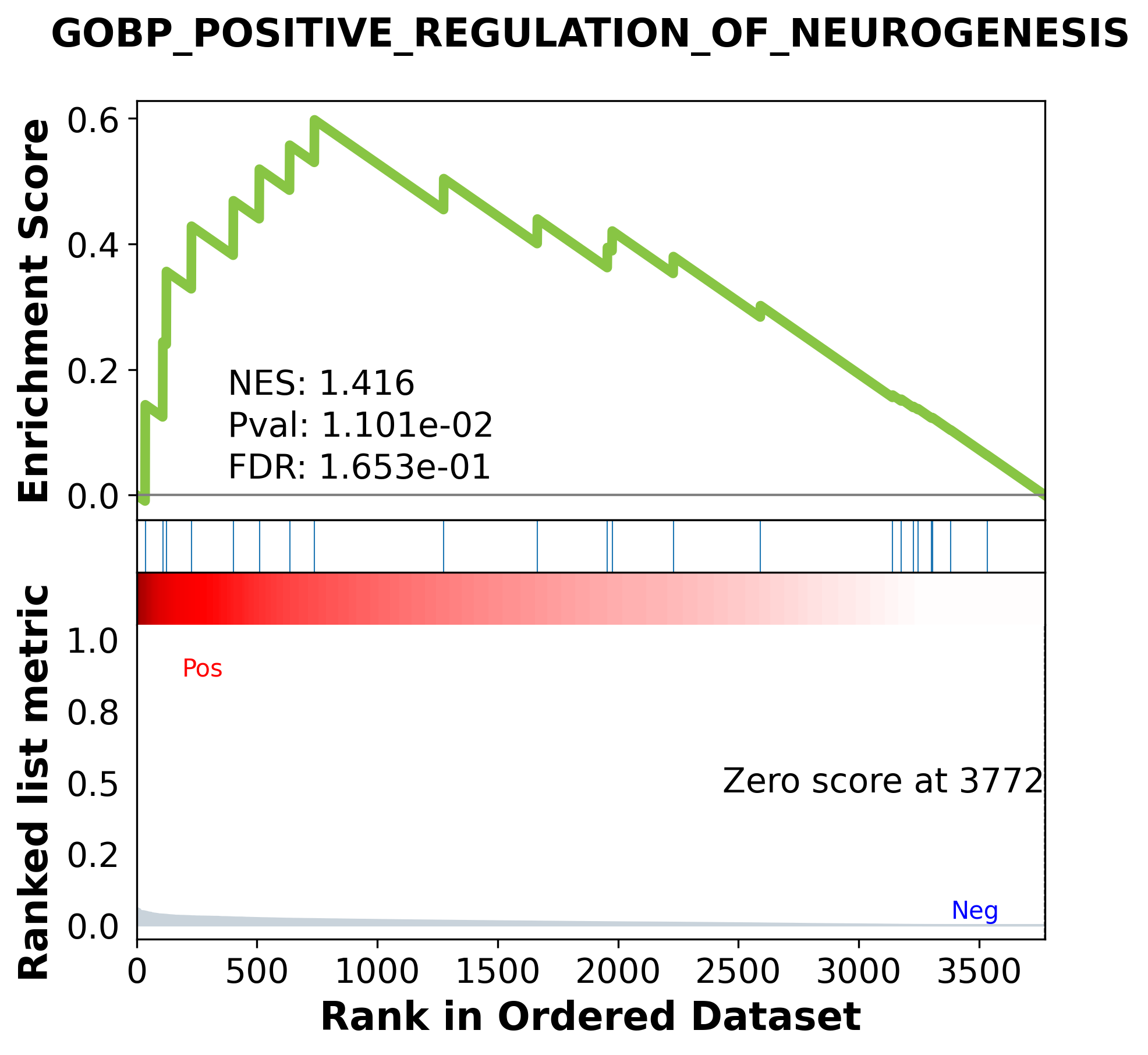

Supplement: Supplemental GSEA [file jciinsight-8-173374-s056.zip › GSEA/Factor 5/prerank/GOBP_POSITIVE_REGULATION_OF_NEUROGENESIS.png]

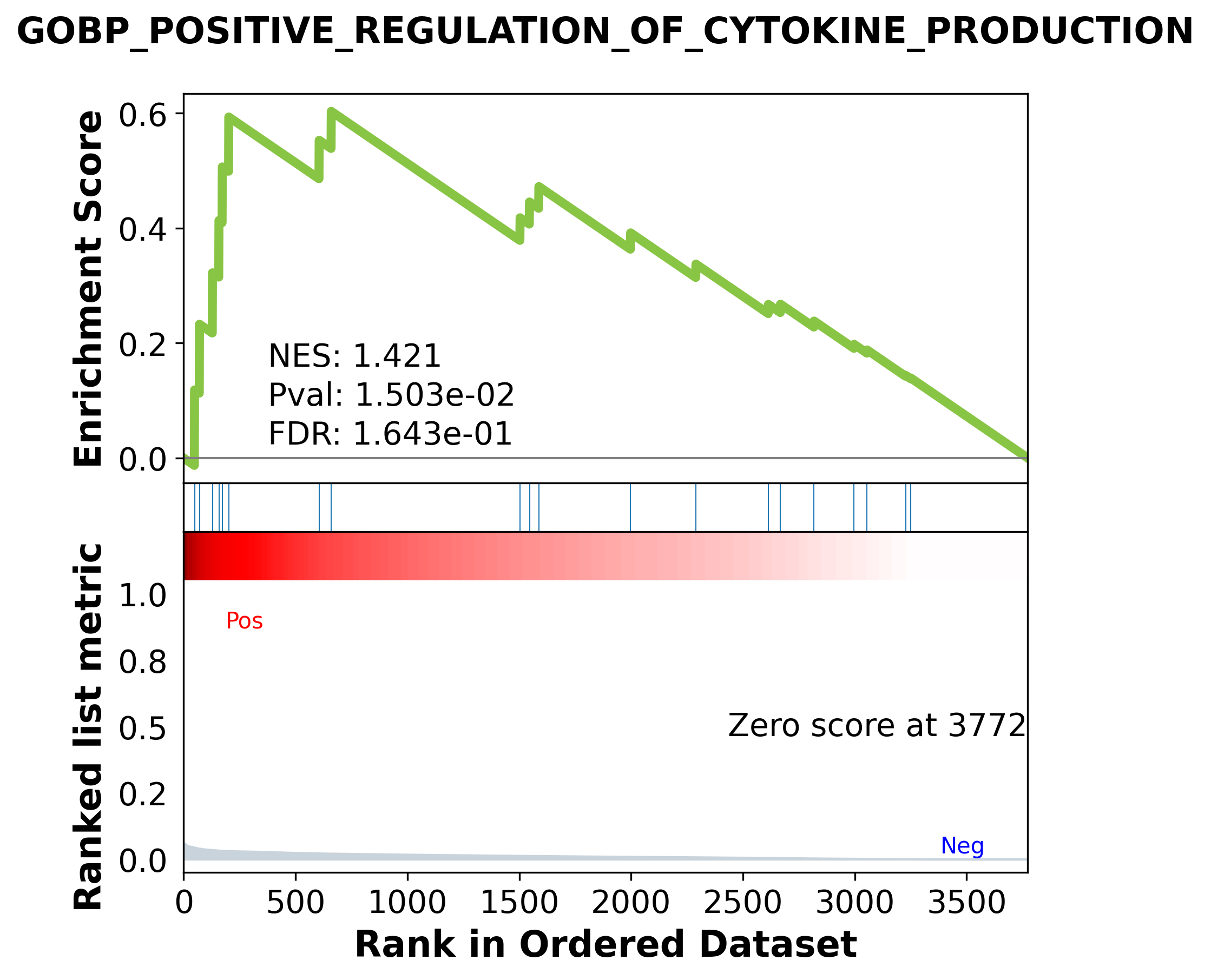

Supplement: Supplemental GSEA [file jciinsight-8-173374-s056.zip › GSEA/Factor 5/prerank/GOBP_POSITIVE_REGULATION_OF_CYTOKINE_PRODUCTION.png]

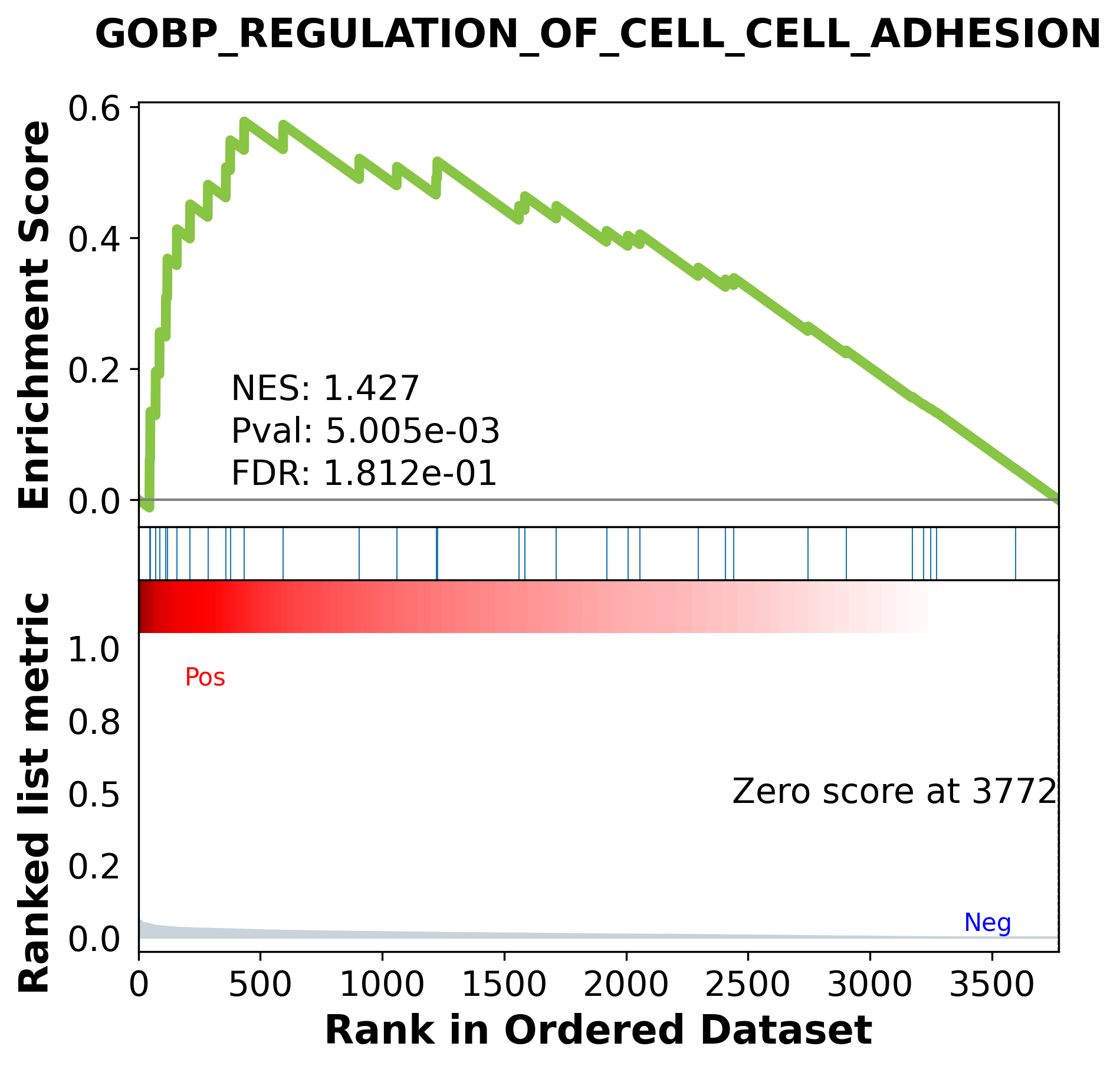

Supplement: Supplemental GSEA [file jciinsight-8-173374-s056.zip › GSEA/Factor 5/prerank/GOBP_REGULATION_OF_CELL_CELL_ADHESION.png]

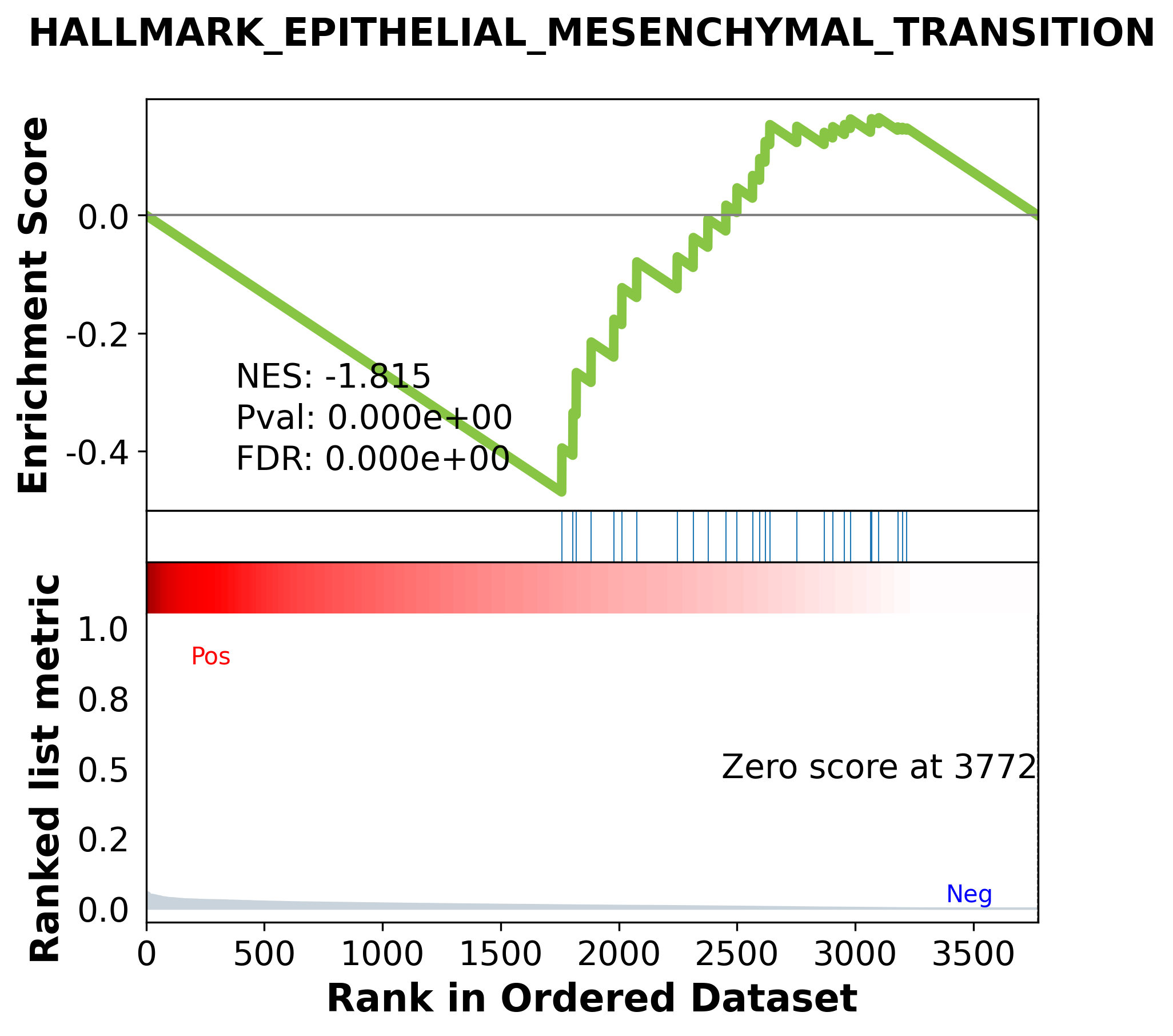

Supplement: Supplemental GSEA [file jciinsight-8-173374-s056.zip › GSEA/Factor 5/prerank/HALLMARK_EPITHELIAL_MESENCHYMAL_TRANSITION.png]

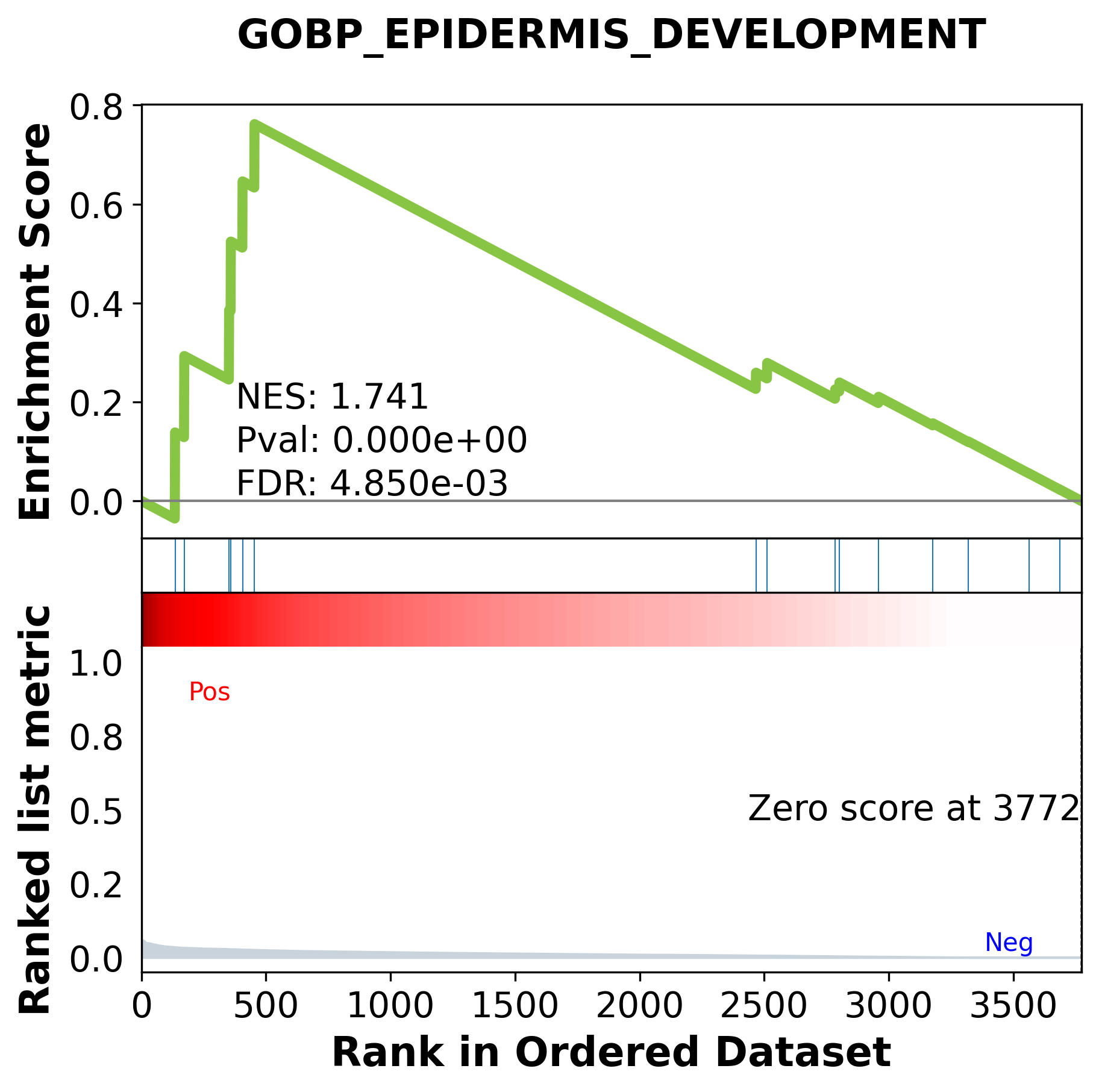

Supplement: Supplemental GSEA [file jciinsight-8-173374-s056.zip › GSEA/Factor 5/prerank/GOBP_EPIDERMIS_DEVELOPMENT.png]

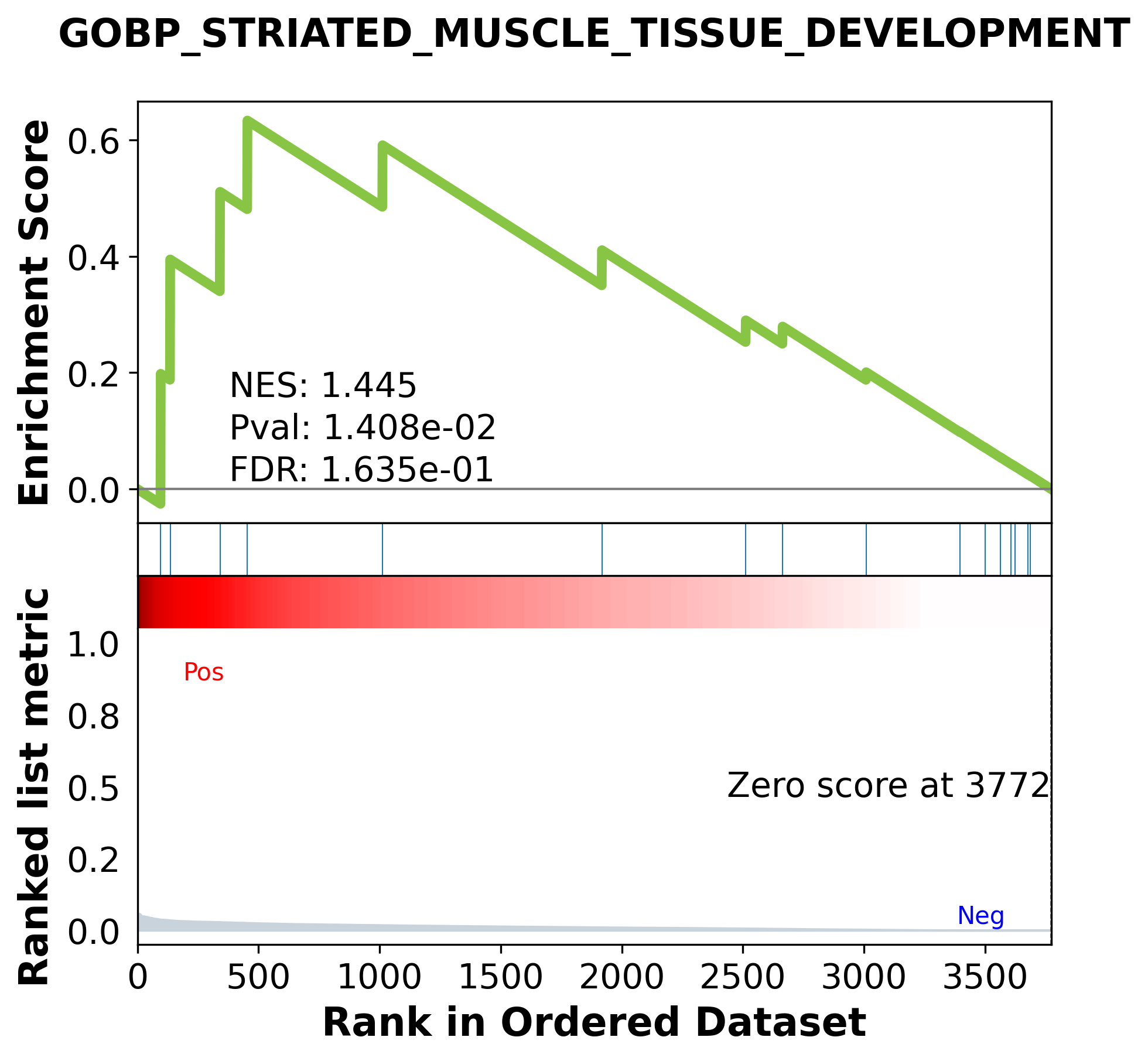

Supplement: Supplemental GSEA [file jciinsight-8-173374-s056.zip › GSEA/Factor 5/prerank/GOBP_STRIATED_MUSCLE_TISSUE_DEVELOPMENT.png]

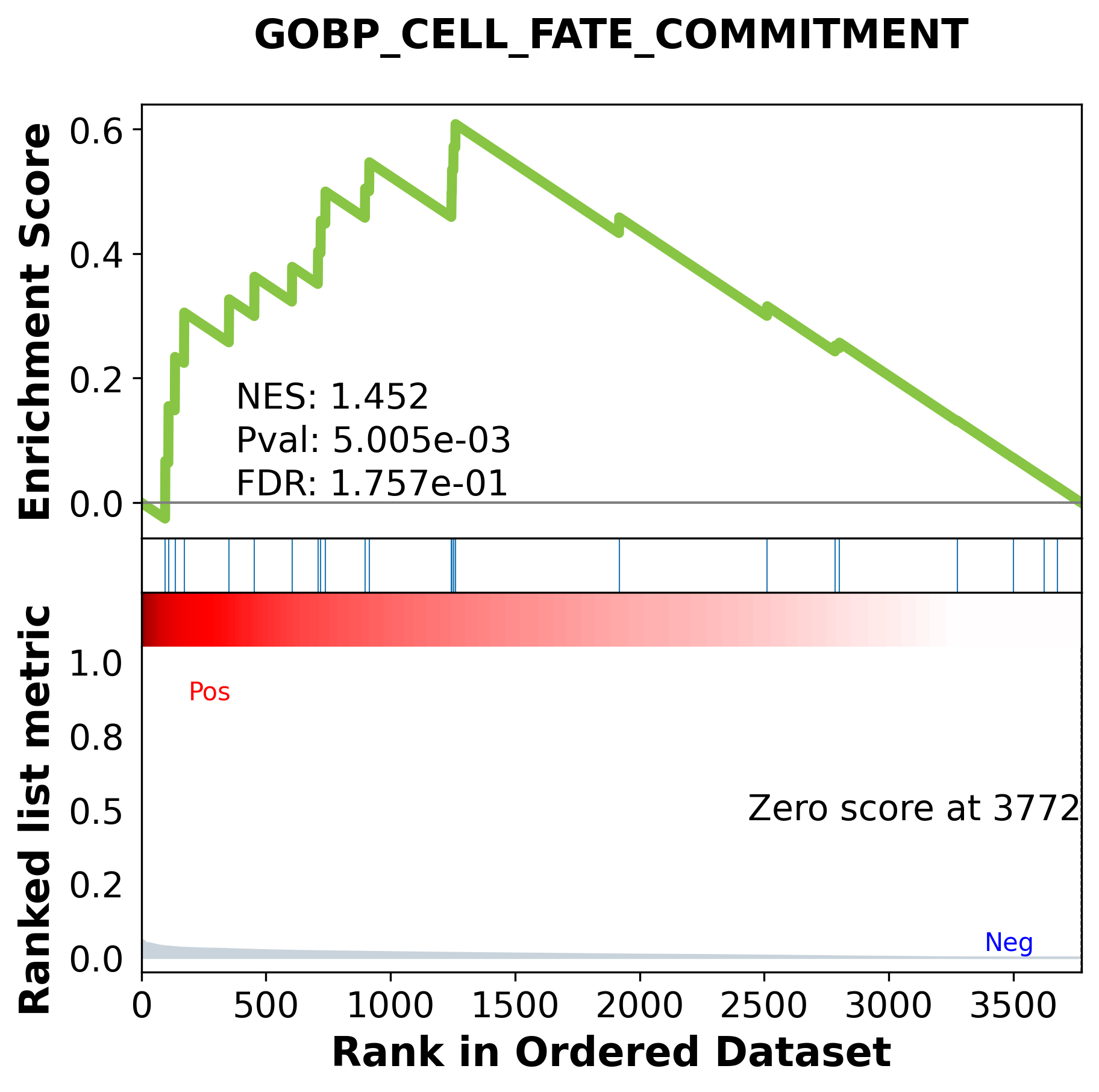

Supplement: Supplemental GSEA [file jciinsight-8-173374-s056.zip › GSEA/Factor 5/prerank/GOBP_CELL_FATE_COMMITMENT.png]

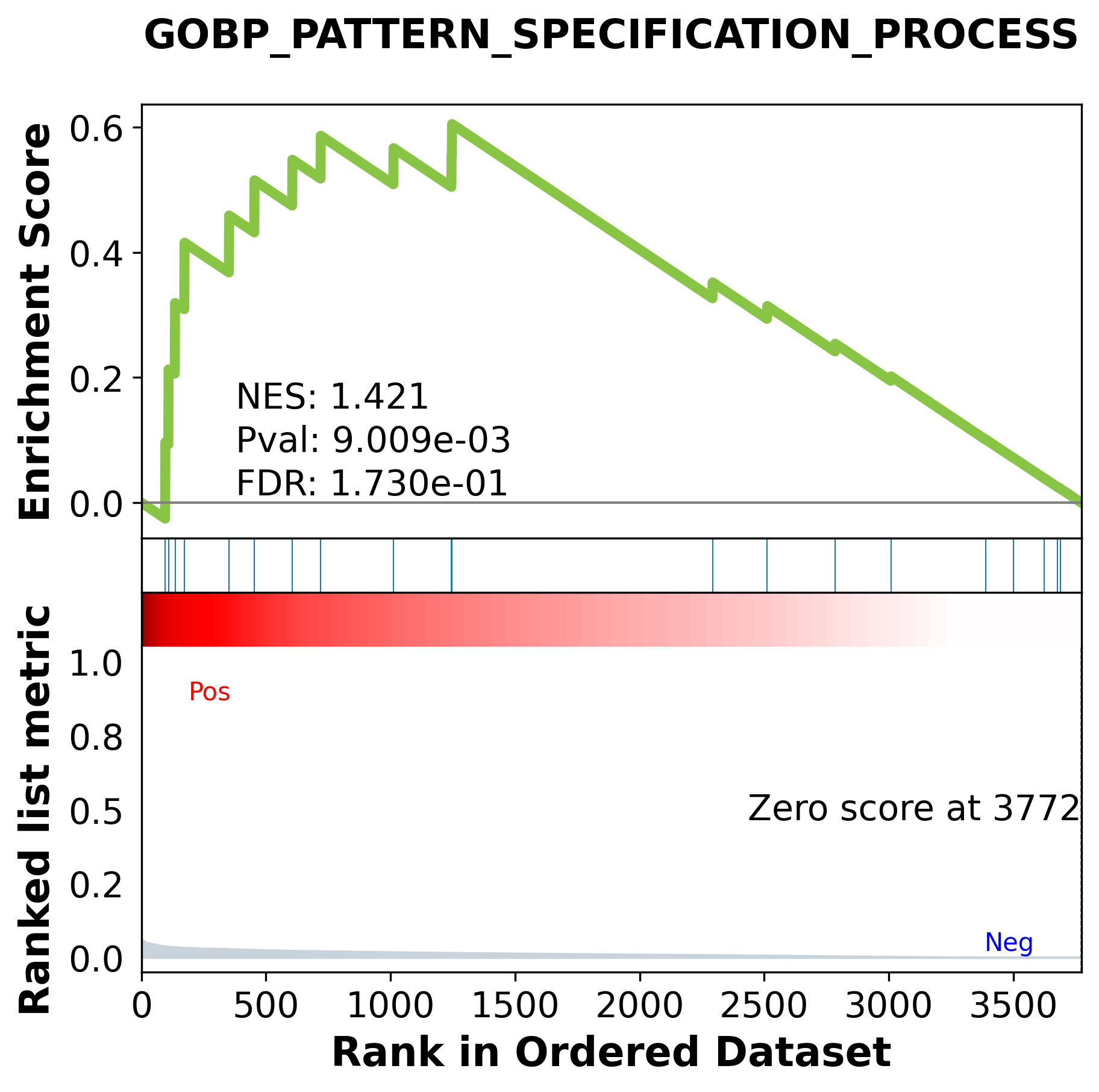

Supplement: Supplemental GSEA [file jciinsight-8-173374-s056.zip › GSEA/Factor 5/prerank/GOBP_PATTERN_SPECIFICATION_PROCESS.png]

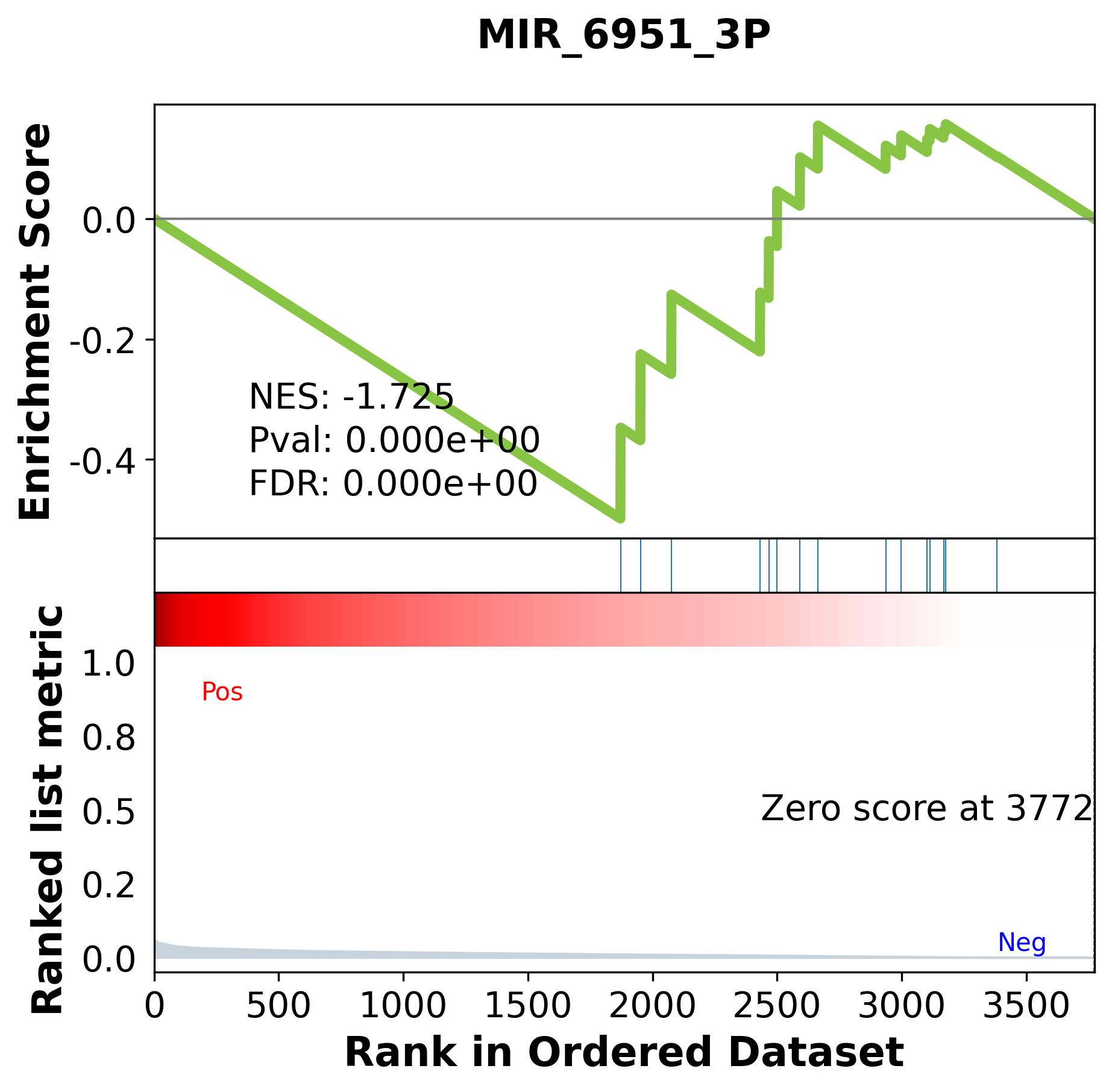

Supplement: Supplemental GSEA [file jciinsight-8-173374-s056.zip › GSEA/Factor 5/prerank/MIR_6951_3P.png]

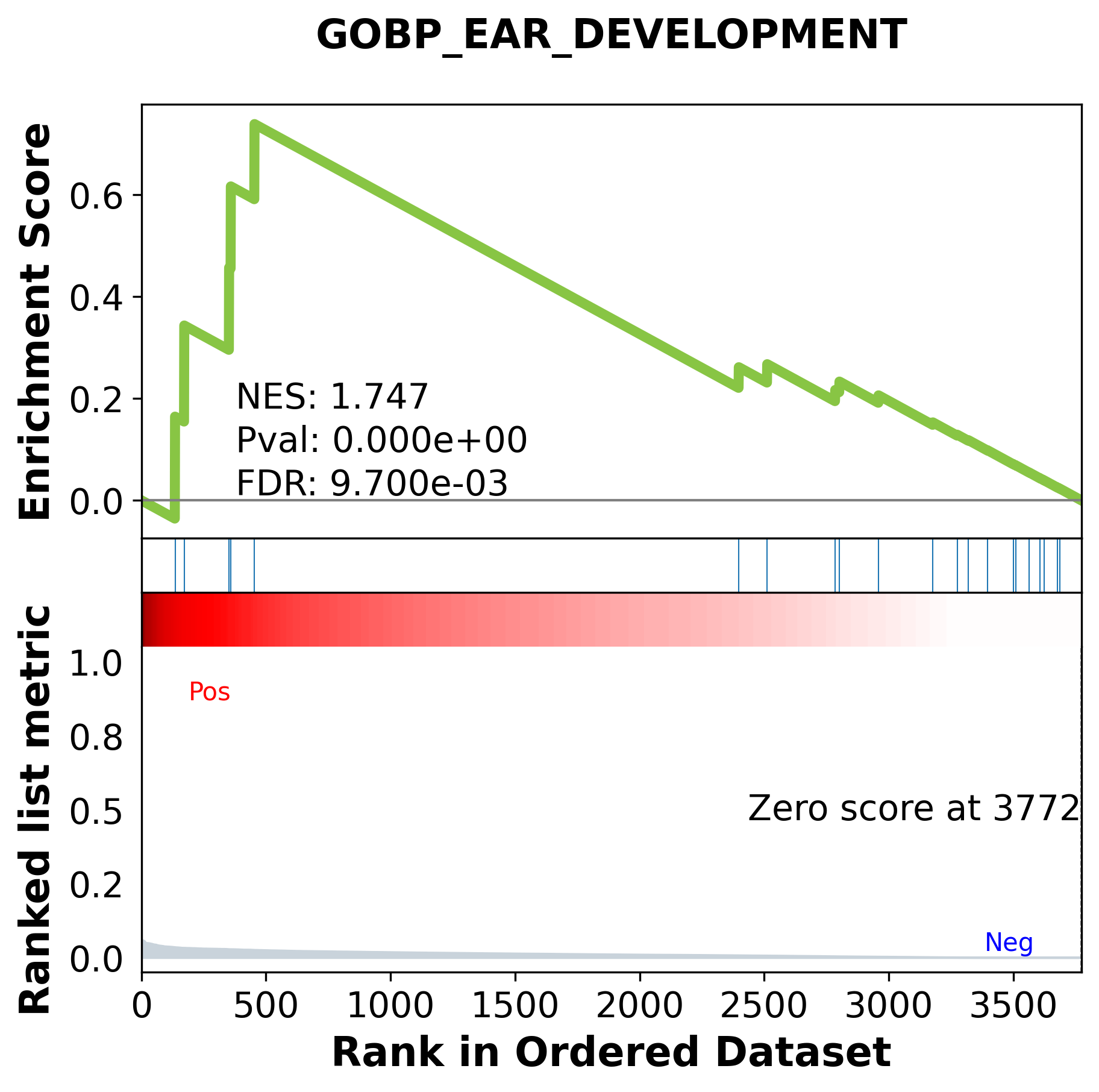

Supplement: Supplemental GSEA [file jciinsight-8-173374-s056.zip › GSEA/Factor 5/prerank/GOBP_EAR_DEVELOPMENT.png]

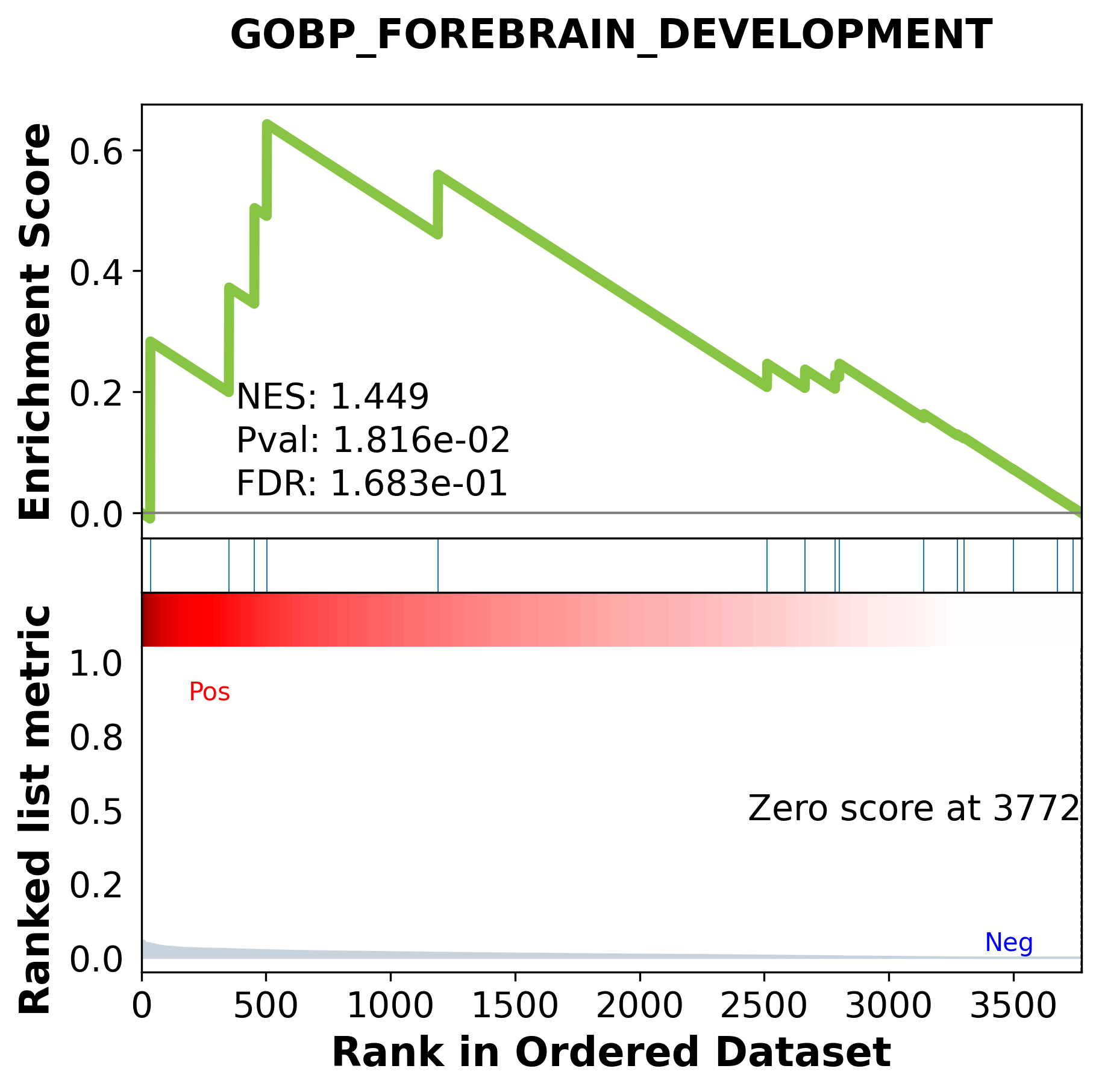

Supplement: Supplemental GSEA [file jciinsight-8-173374-s056.zip › GSEA/Factor 5/prerank/GOBP_FOREBRAIN_DEVELOPMENT.png]

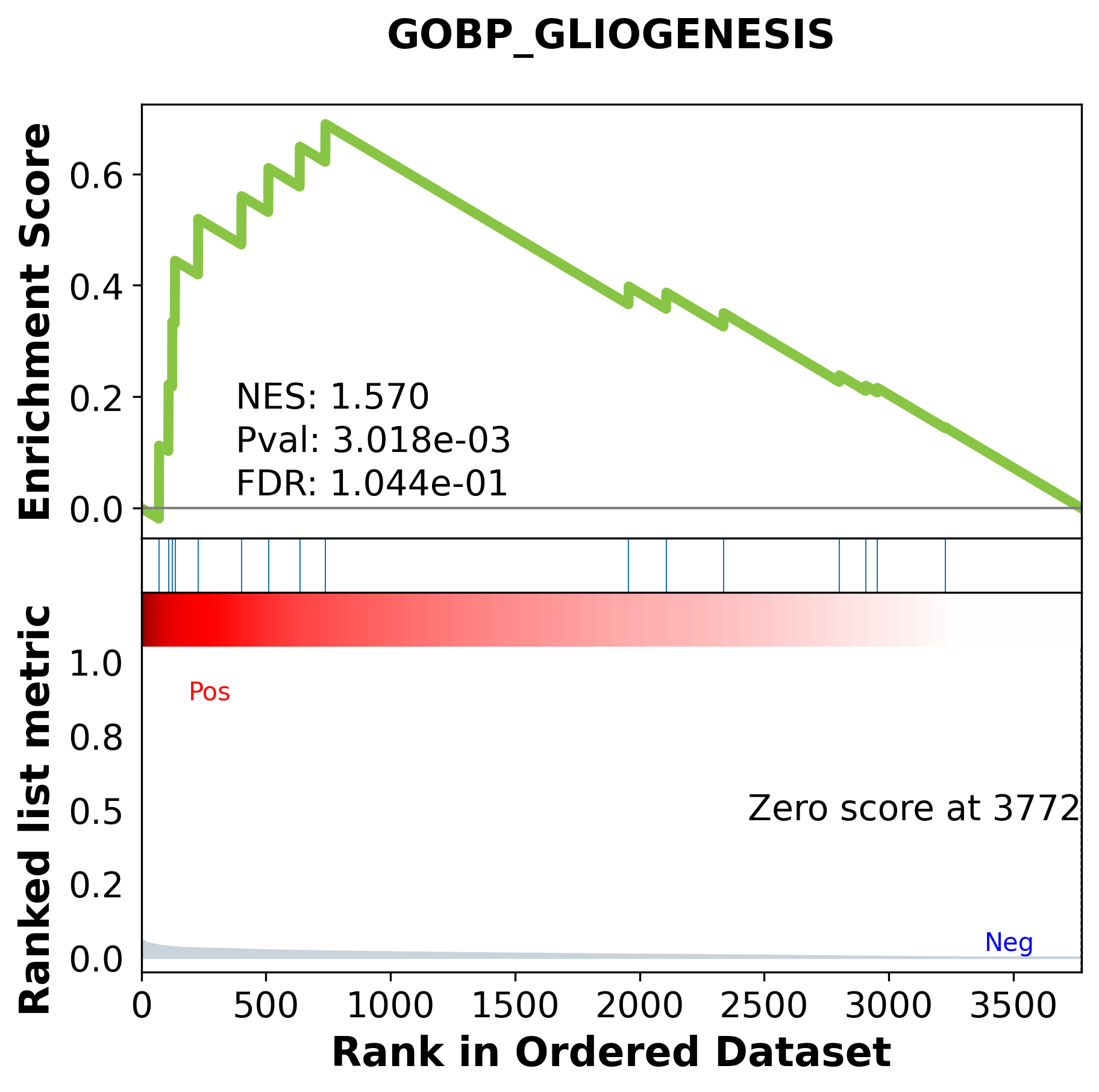

Supplement: Supplemental GSEA [file jciinsight-8-173374-s056.zip › GSEA/Factor 5/prerank/GOBP_GLIOGENESIS.png]

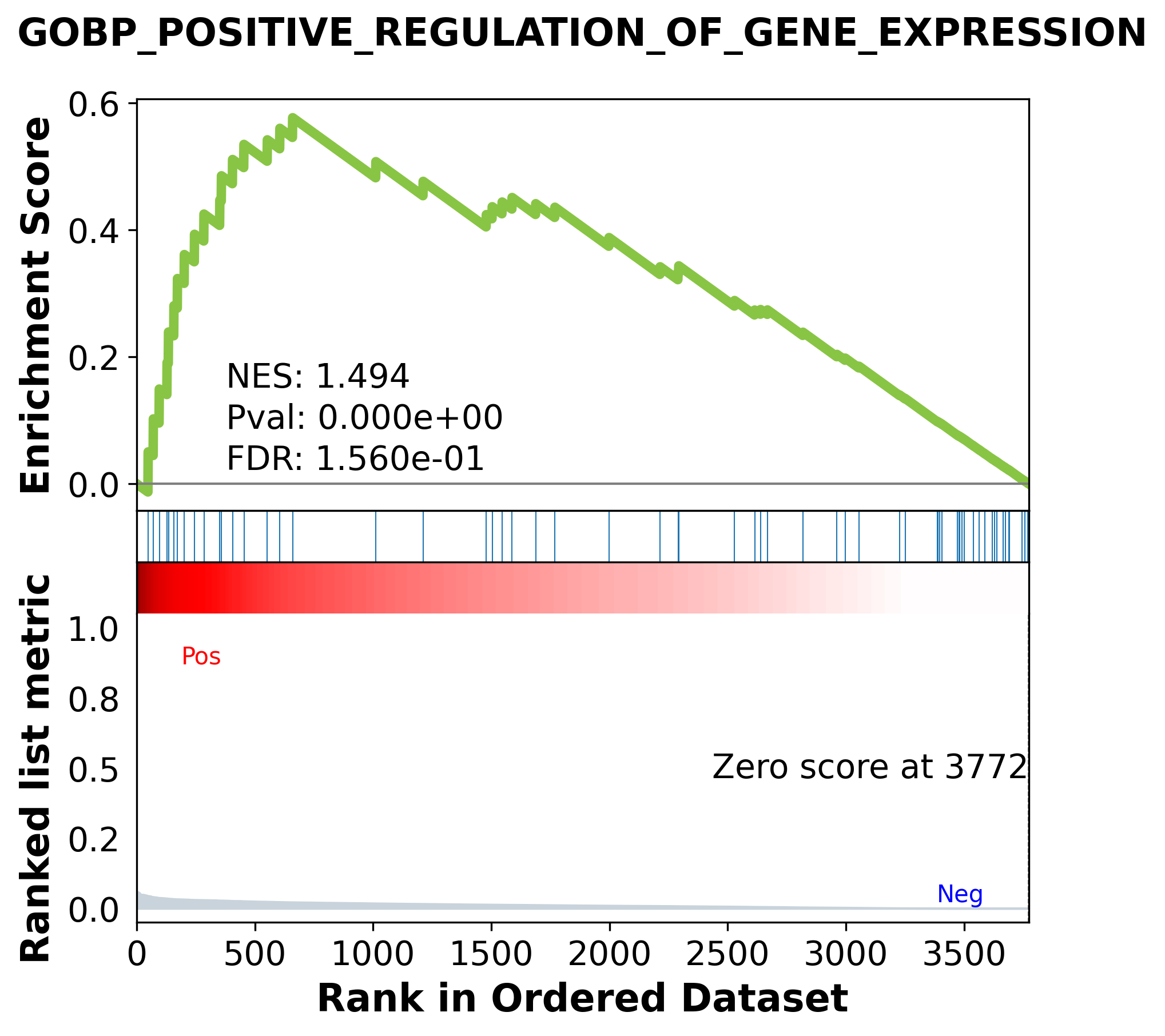

Supplement: Supplemental GSEA [file jciinsight-8-173374-s056.zip › GSEA/Factor 5/prerank/GOBP_POSITIVE_REGULATION_OF_GENE_EXPRESSION.png]

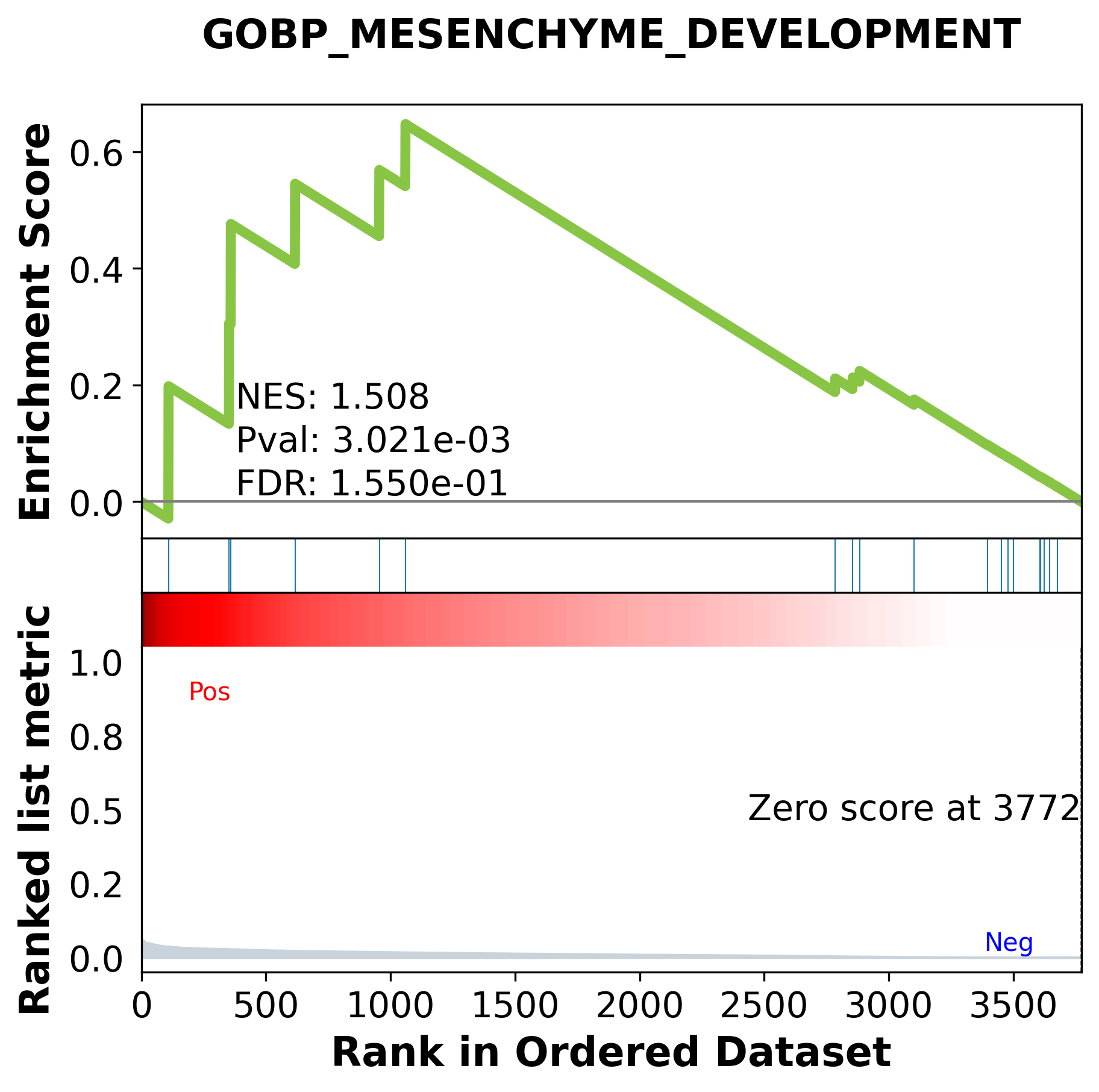

Supplement: Supplemental GSEA [file jciinsight-8-173374-s056.zip › GSEA/Factor 5/prerank/GOBP_MESENCHYME_DEVELOPMENT.png]

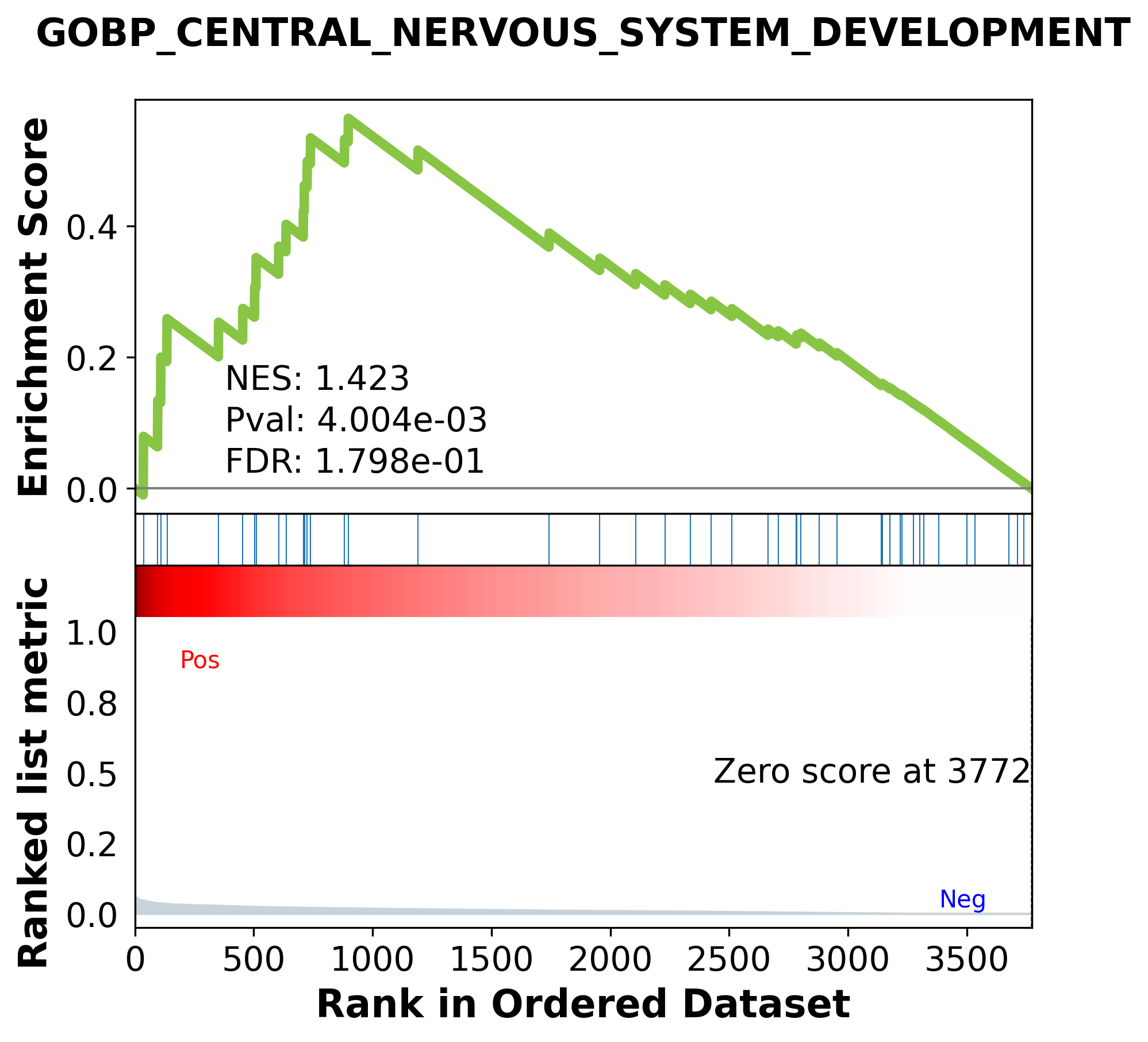

Supplement: Supplemental GSEA [file jciinsight-8-173374-s056.zip › GSEA/Factor 5/prerank/GOBP_CENTRAL_NERVOUS_SYSTEM_DEVELOPMENT.png]

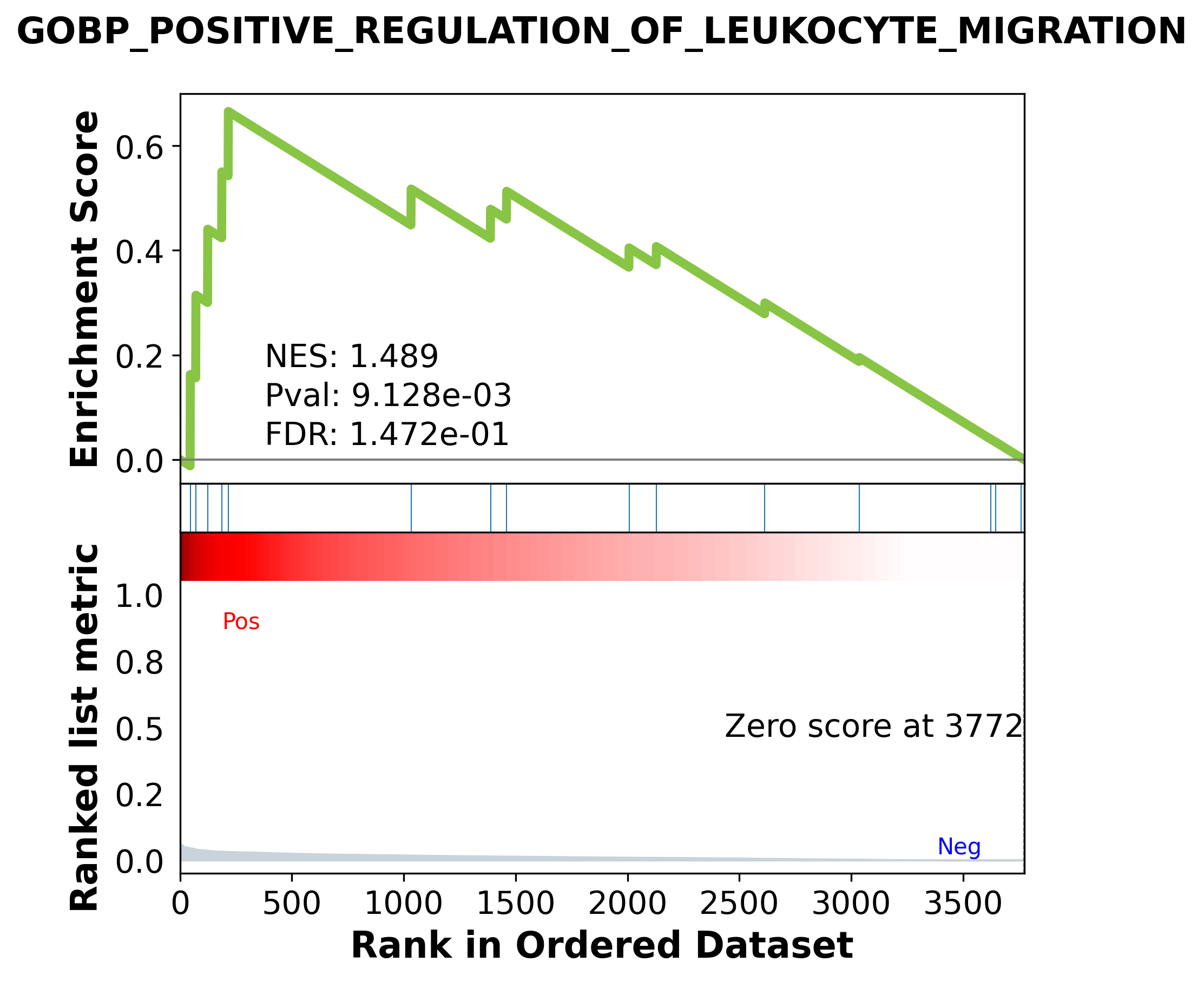

Supplement: Supplemental GSEA [file jciinsight-8-173374-s056.zip › GSEA/Factor 5/prerank/GOBP_POSITIVE_REGULATION_OF_LEUKOCYTE_MIGRATION.png]

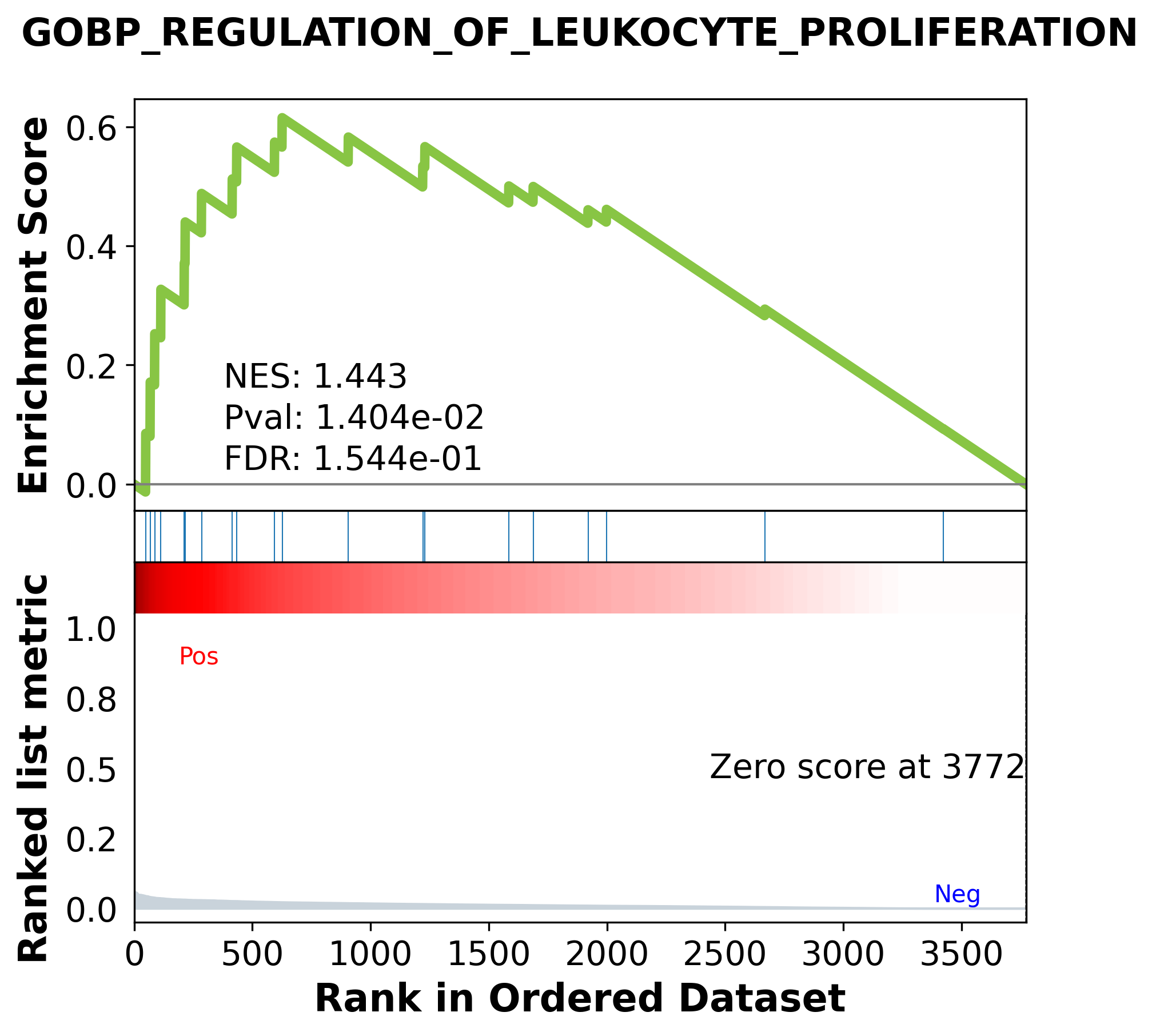

Supplement: Supplemental GSEA [file jciinsight-8-173374-s056.zip › GSEA/Factor 5/prerank/GOBP_REGULATION_OF_LEUKOCYTE_PROLIFERATION.png]

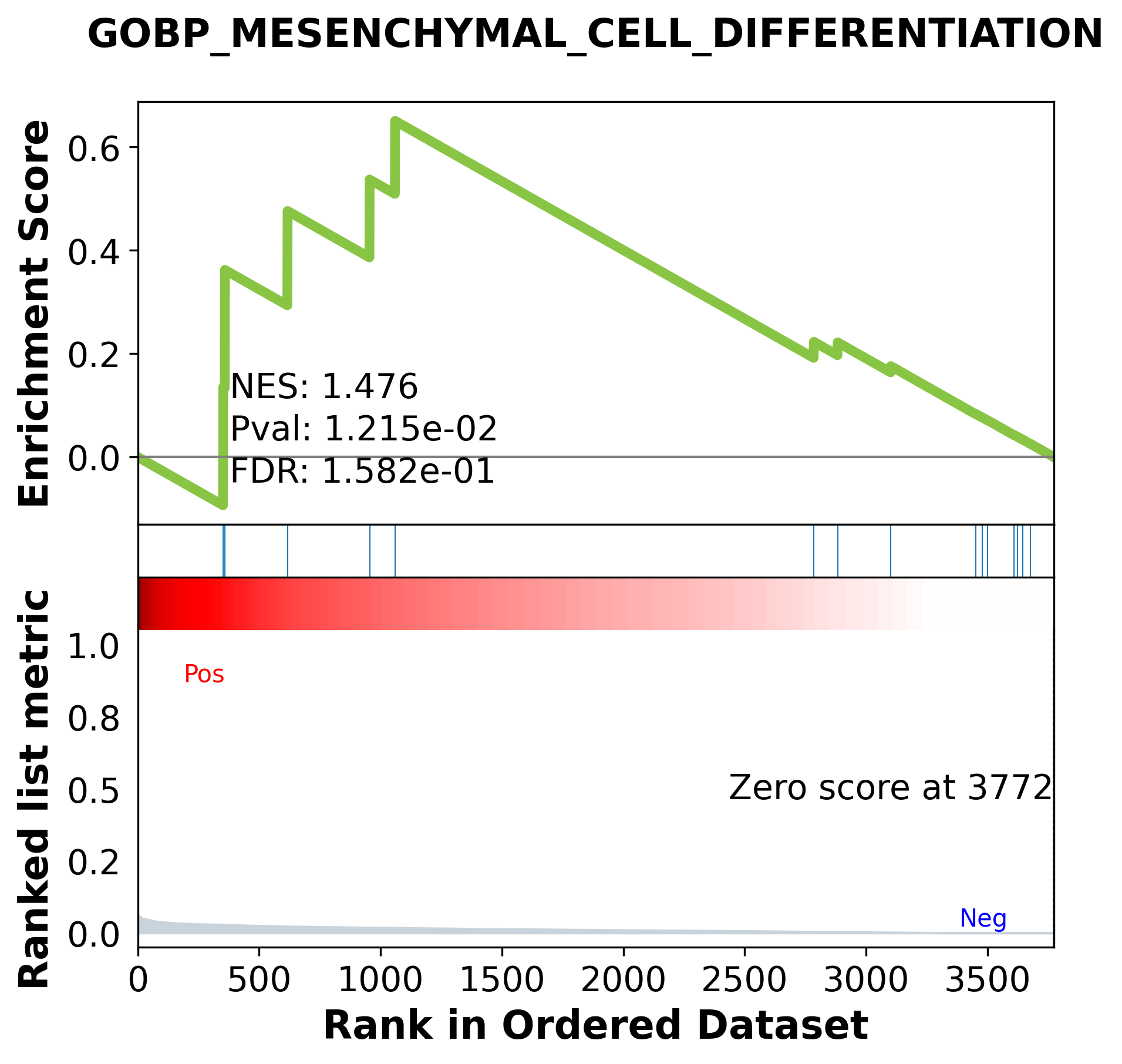

Supplement: Supplemental GSEA [file jciinsight-8-173374-s056.zip › GSEA/Factor 5/prerank/GOBP_MESENCHYMAL_CELL_DIFFERENTIATION.png]

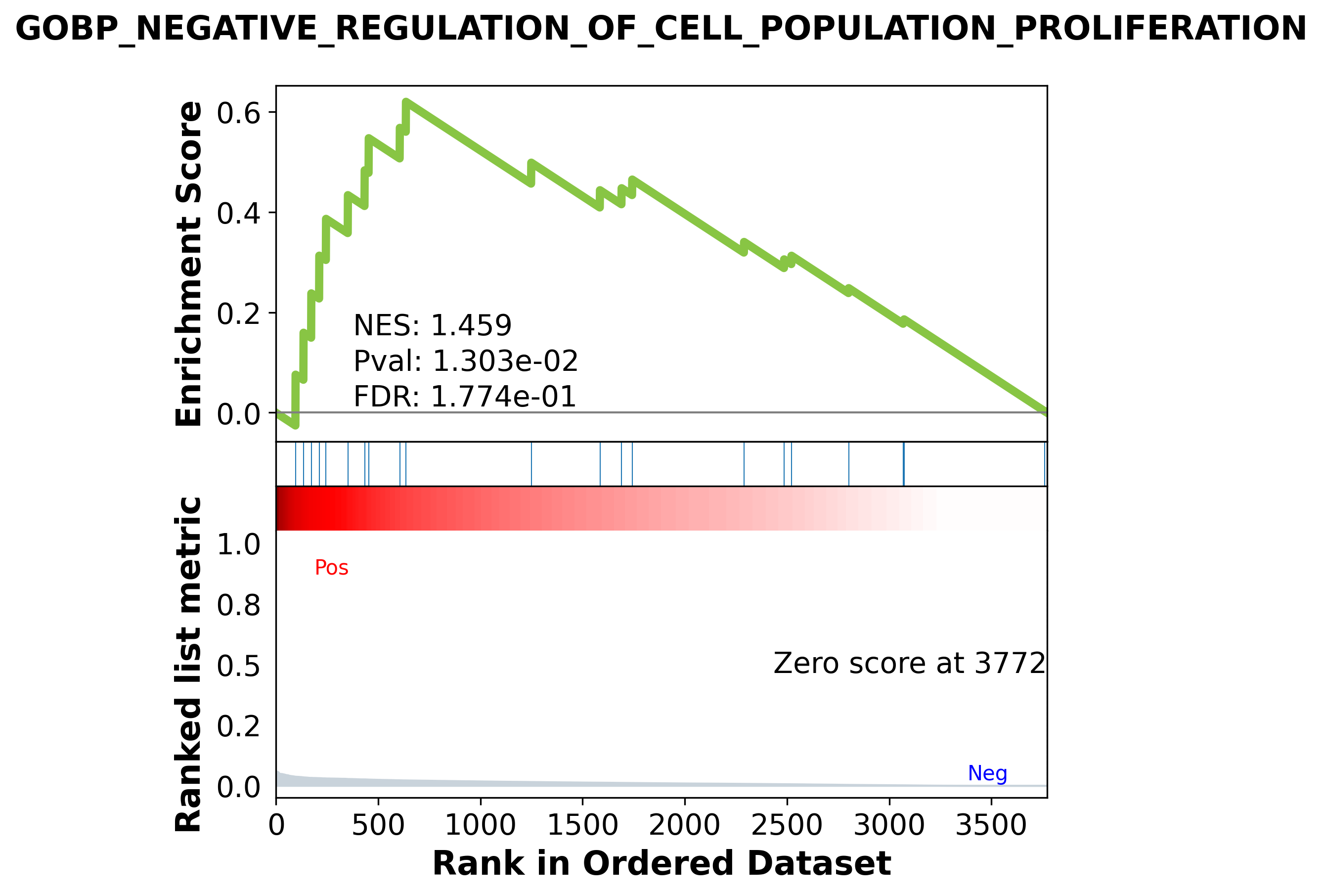

Supplement: Supplemental GSEA [file jciinsight-8-173374-s056.zip › GSEA/Factor 5/prerank/GOBP_NEGATIVE_REGULATION_OF_CELL_POPULATION_PROLIFERATION.png]

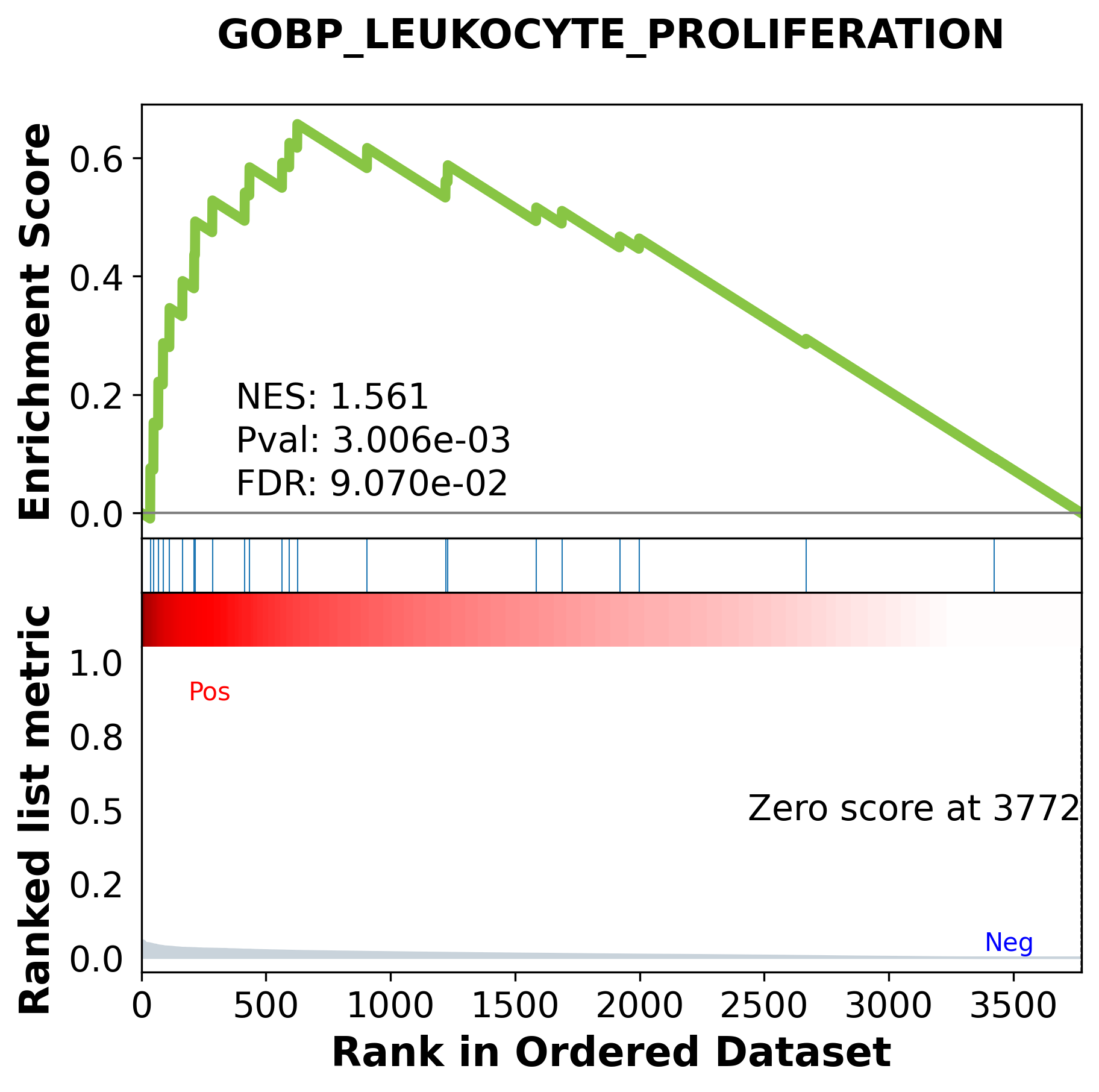

Supplement: Supplemental GSEA [file jciinsight-8-173374-s056.zip › GSEA/Factor 5/prerank/GOBP_LEUKOCYTE_PROLIFERATION.png]

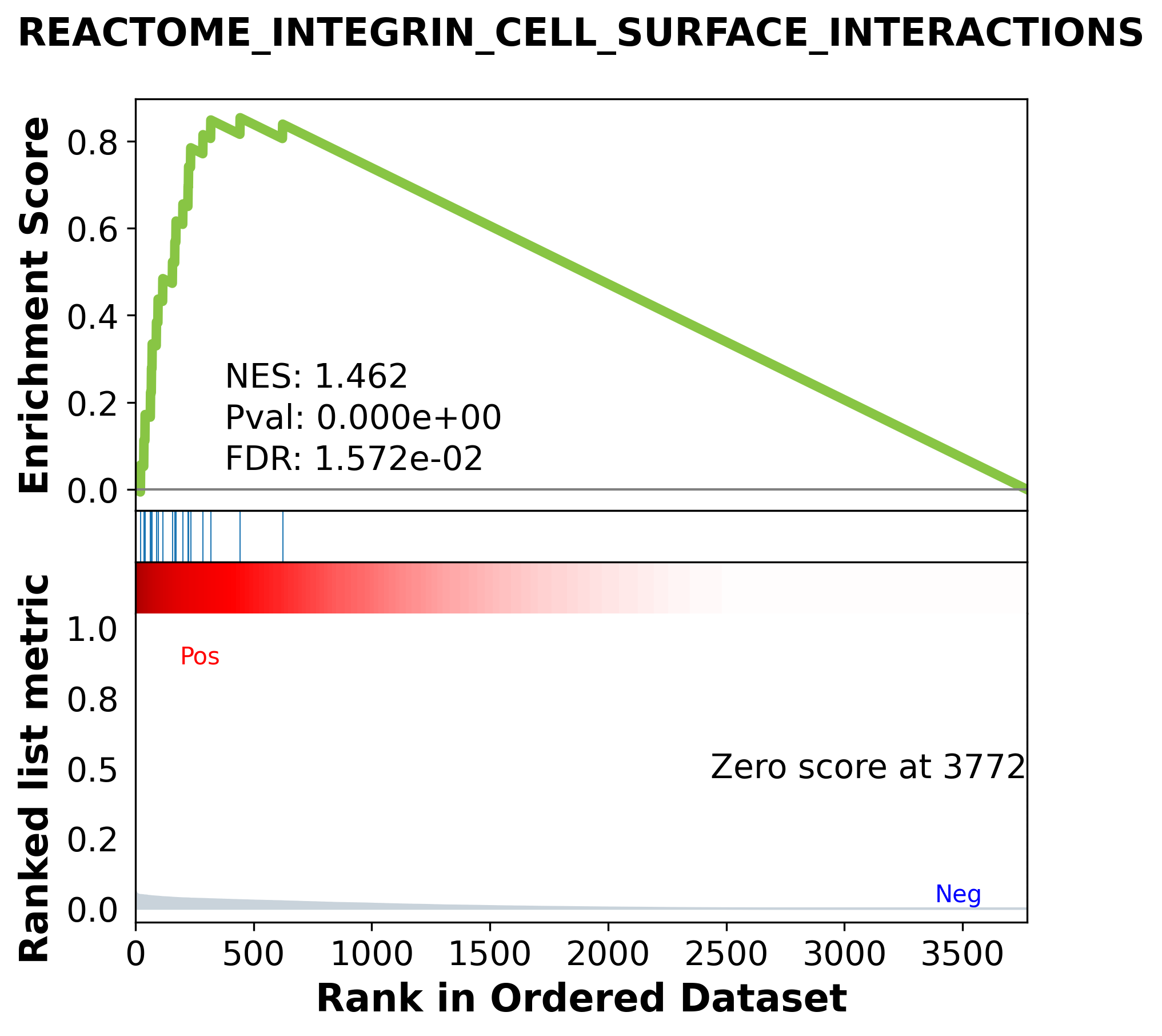

Supplement: Supplemental GSEA [file jciinsight-8-173374-s056.zip › GSEA/Factor 2/prerank/REACTOME_INTEGRIN_CELL_SURFACE_INTERACTIONS.png]

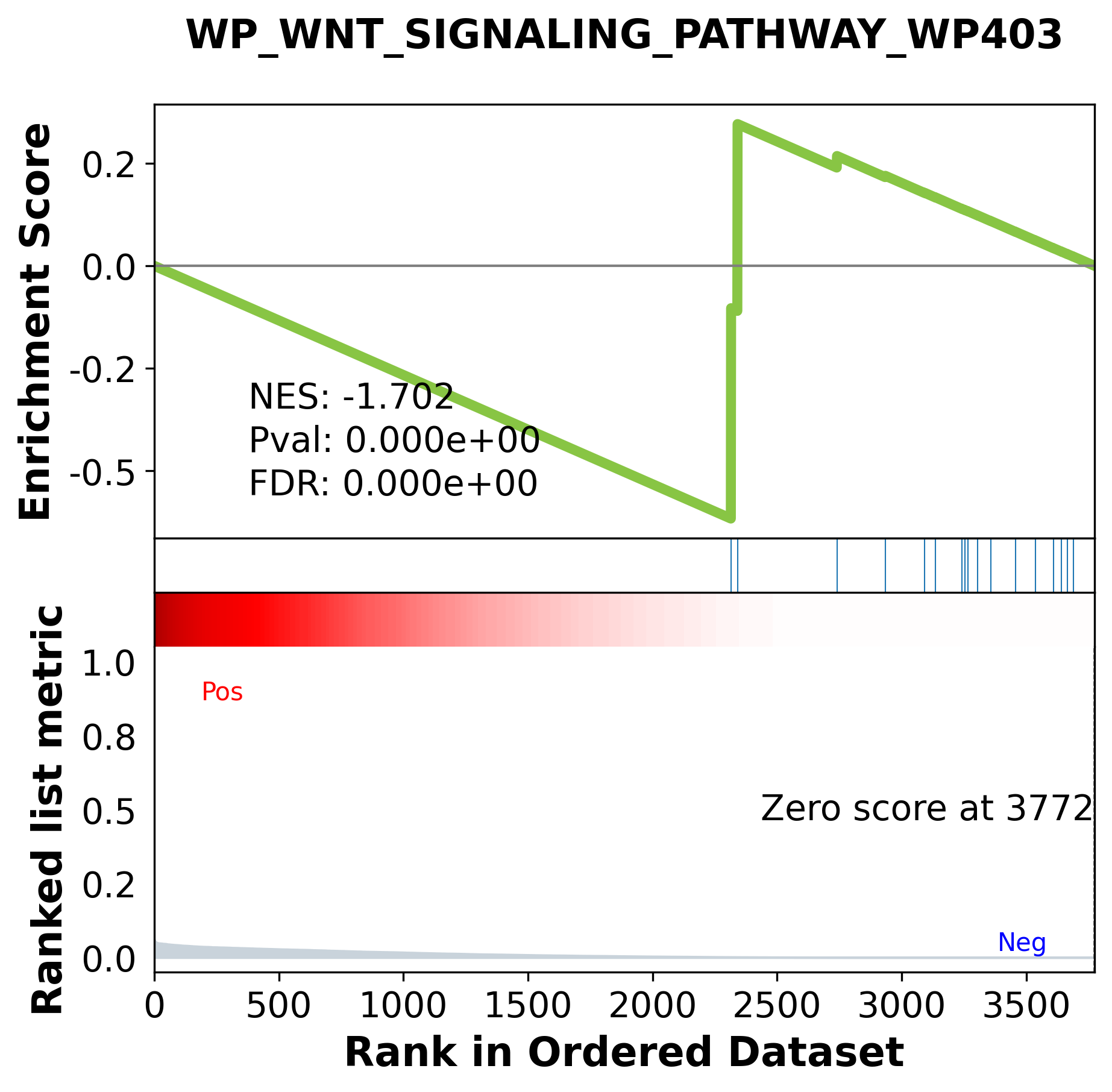

Supplement: Supplemental GSEA [file jciinsight-8-173374-s056.zip › GSEA/Factor 2/prerank/WP_WNT_SIGNALING_PATHWAY_WP403.png]

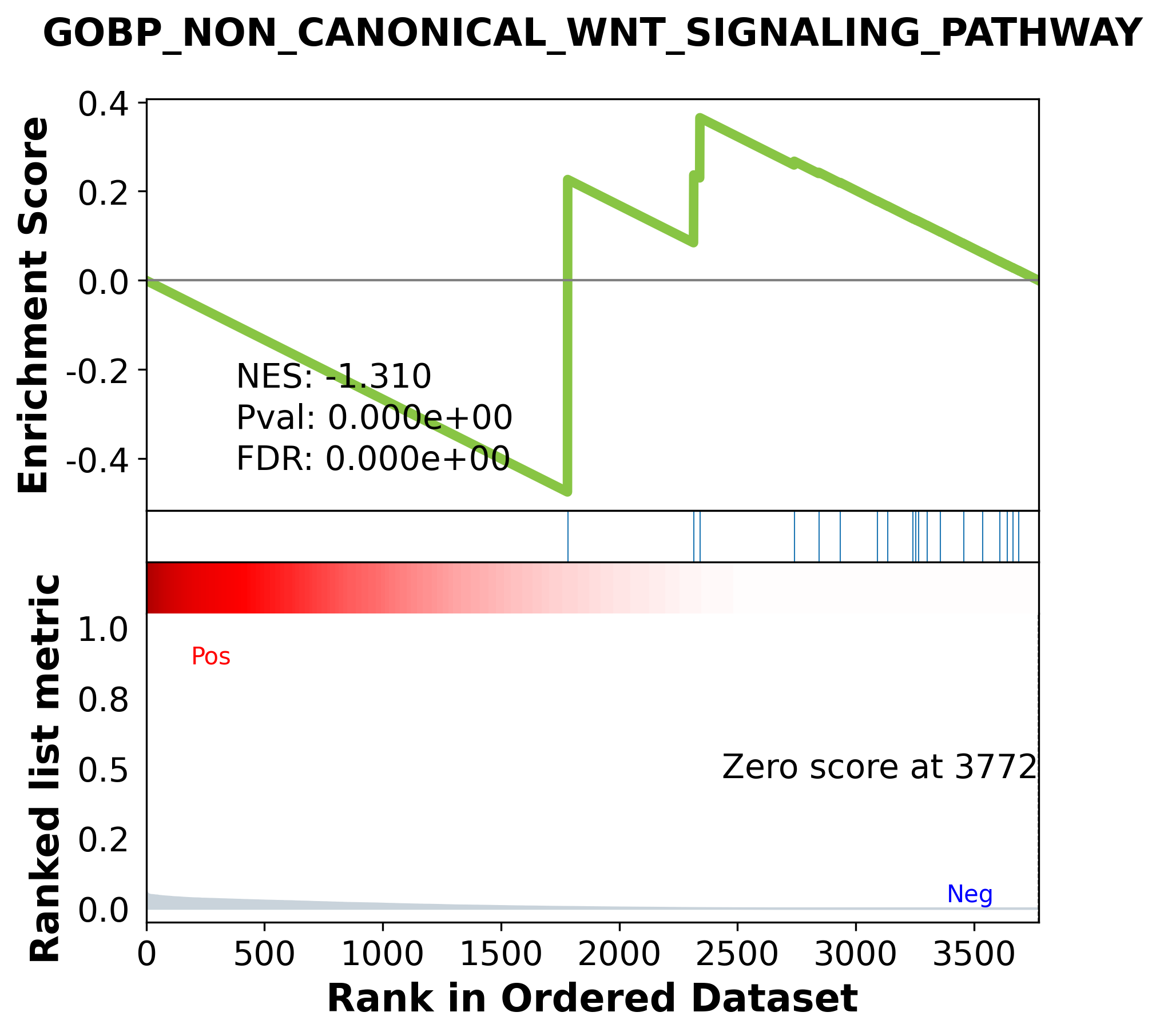

Supplement: Supplemental GSEA [file jciinsight-8-173374-s056.zip › GSEA/Factor 2/prerank/GOBP_NON_CANONICAL_WNT_SIGNALING_PATHWAY.png]

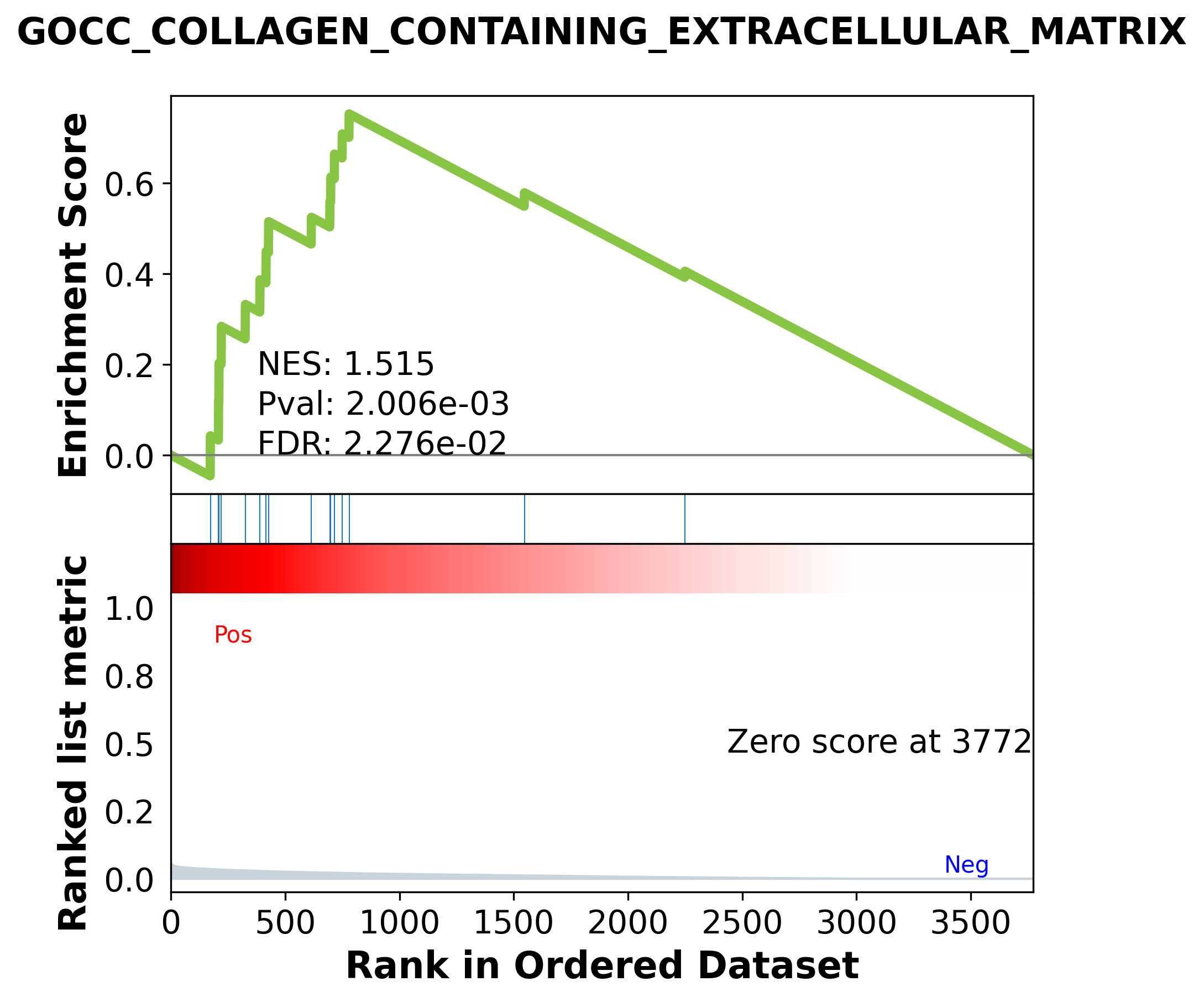

Supplement: Supplemental GSEA [file jciinsight-8-173374-s056.zip › GSEA/Factor 1/prerank/GOCC_COLLAGEN_CONTAINING_EXTRACELLULAR_MATRIX.png]

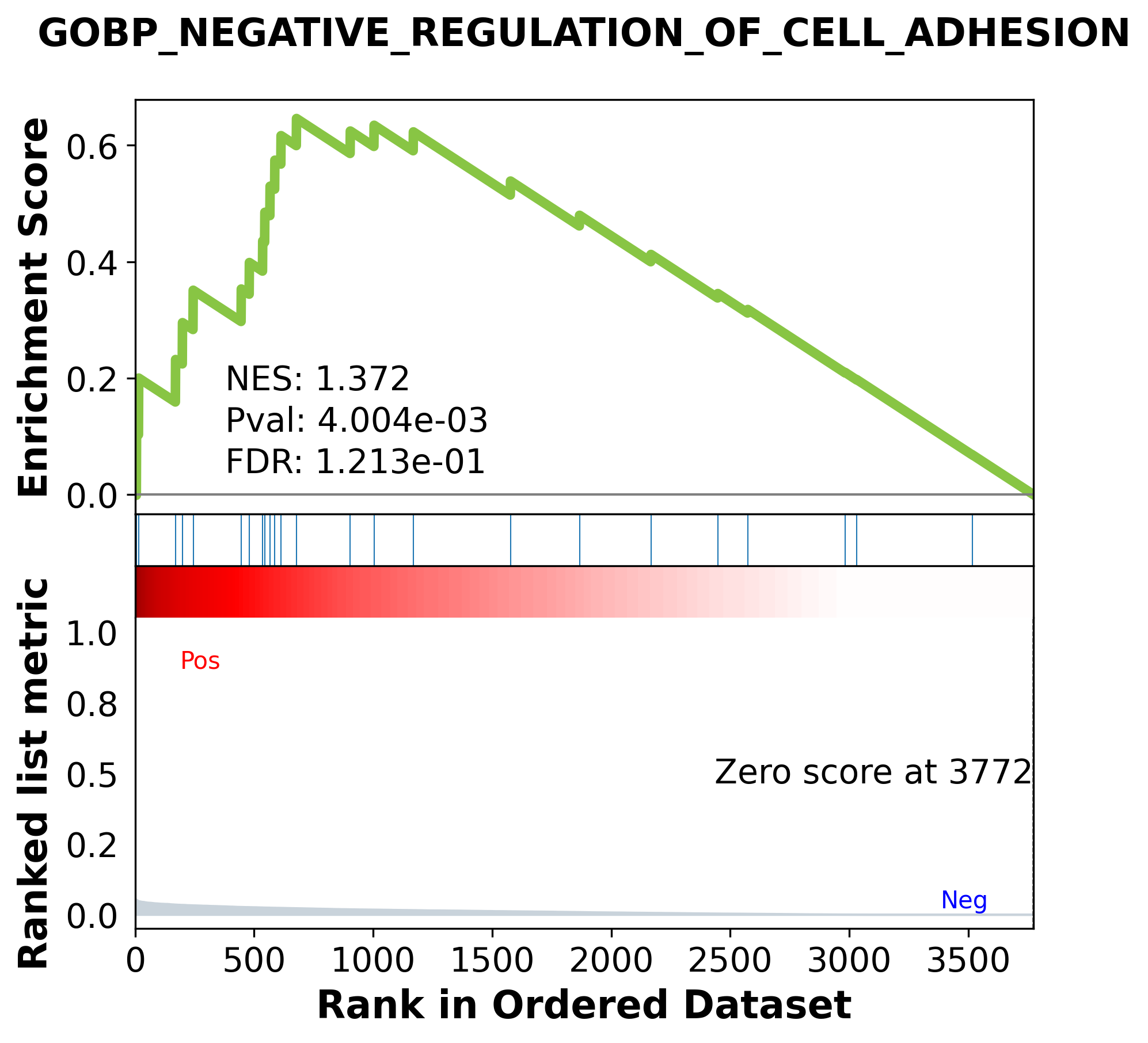

Supplement: Supplemental GSEA [file jciinsight-8-173374-s056.zip › GSEA/Factor 1/prerank/GOBP_NEGATIVE_REGULATION_OF_CELL_ADHESION.png]

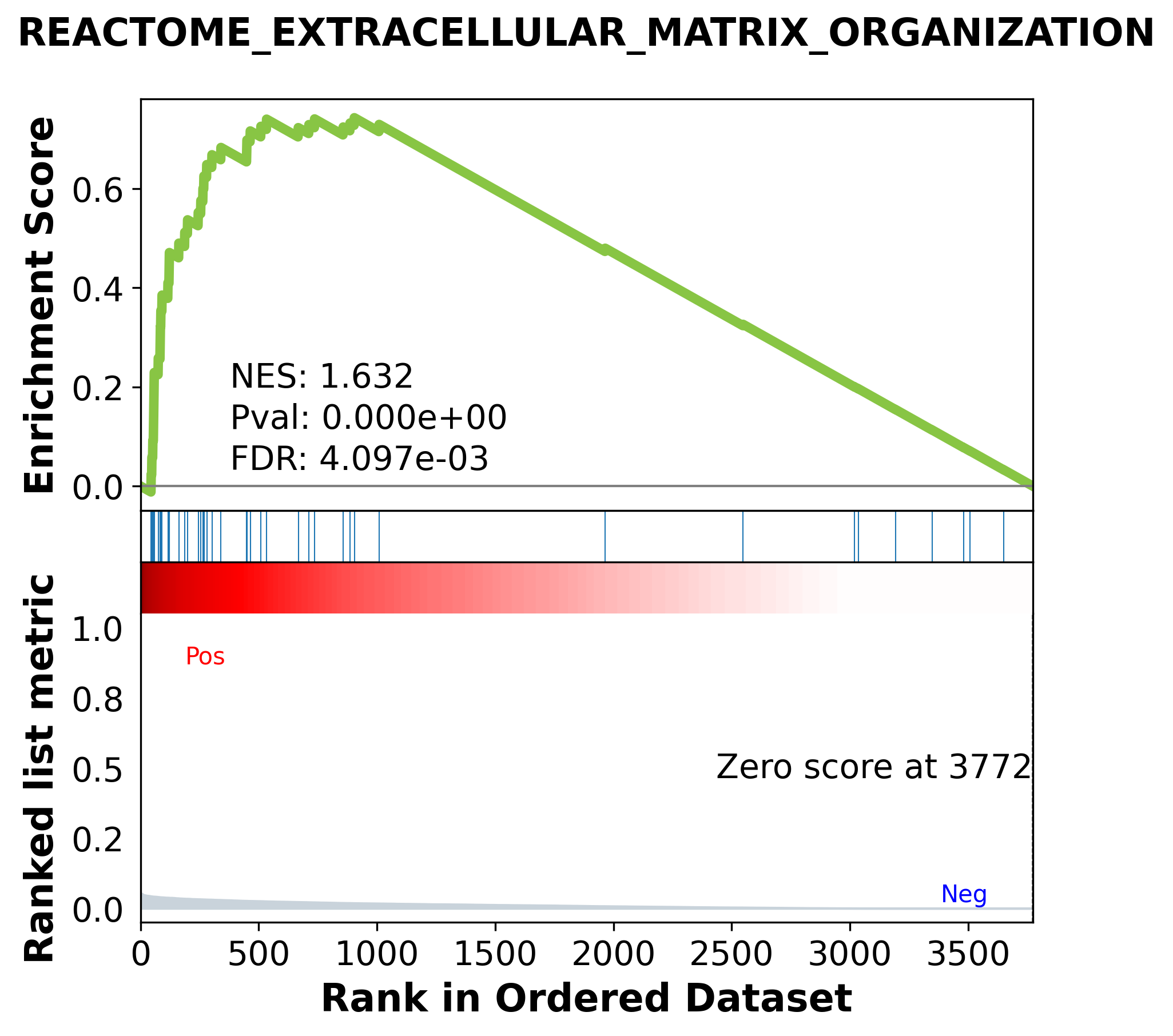

Supplement: Supplemental GSEA [file jciinsight-8-173374-s056.zip › GSEA/Factor 1/prerank/REACTOME_EXTRACELLULAR_MATRIX_ORGANIZATION.png]

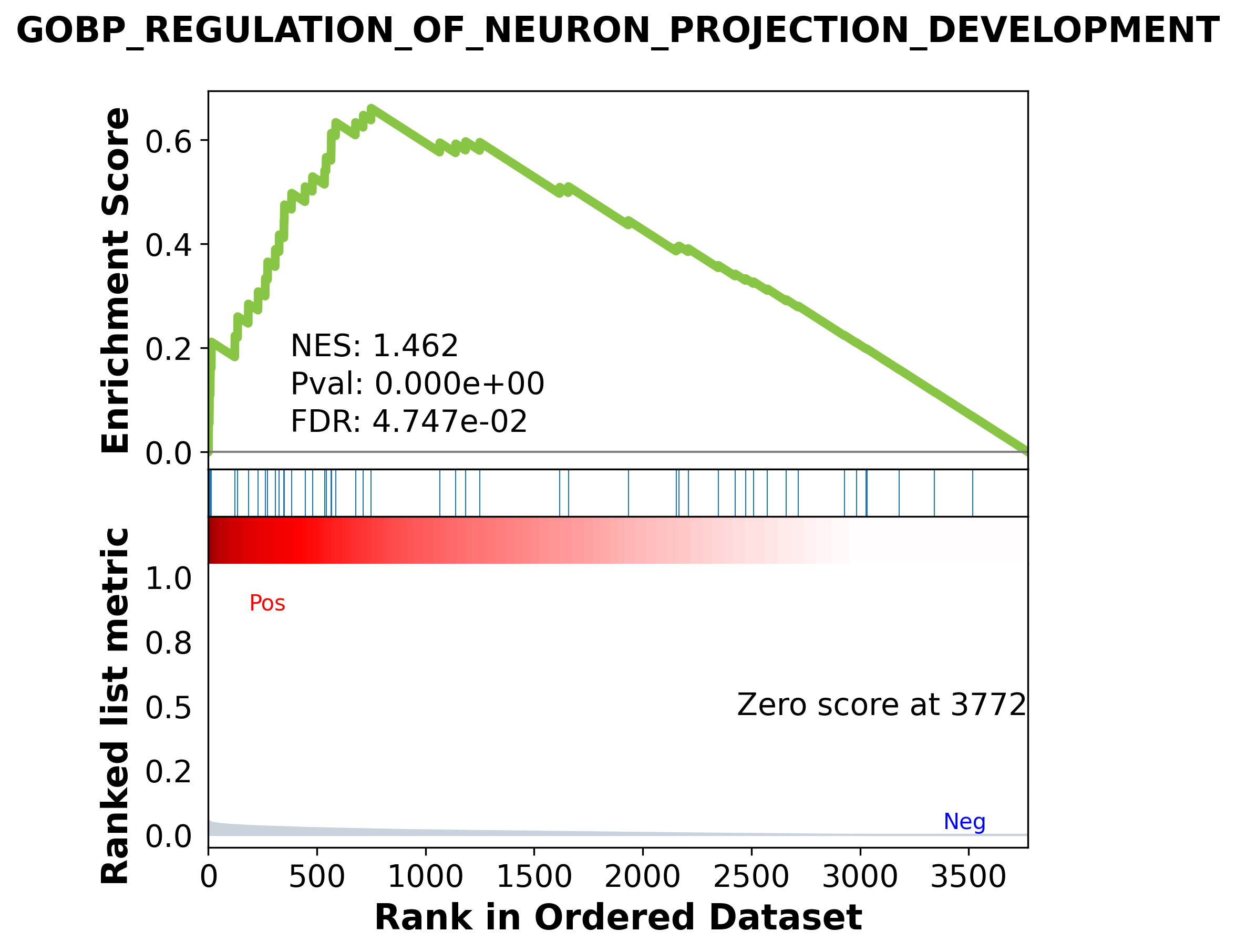

Supplement: Supplemental GSEA [file jciinsight-8-173374-s056.zip › GSEA/Factor 1/prerank/GOBP_REGULATION_OF_NEURON_PROJECTION_DEVELOPMENT.png]

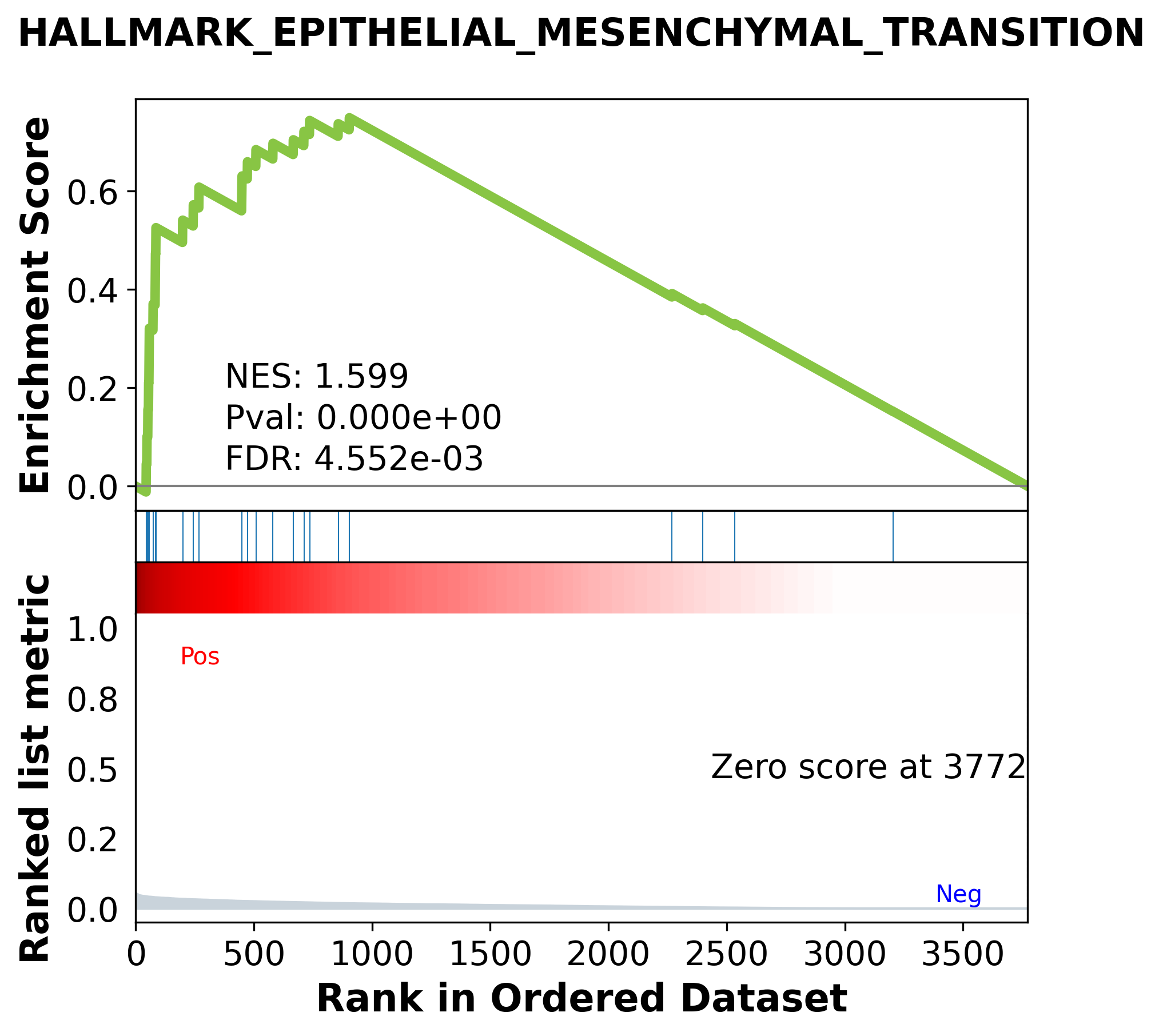

Supplement: Supplemental GSEA [file jciinsight-8-173374-s056.zip › GSEA/Factor 1/prerank/HALLMARK_EPITHELIAL_MESENCHYMAL_TRANSITION.png]

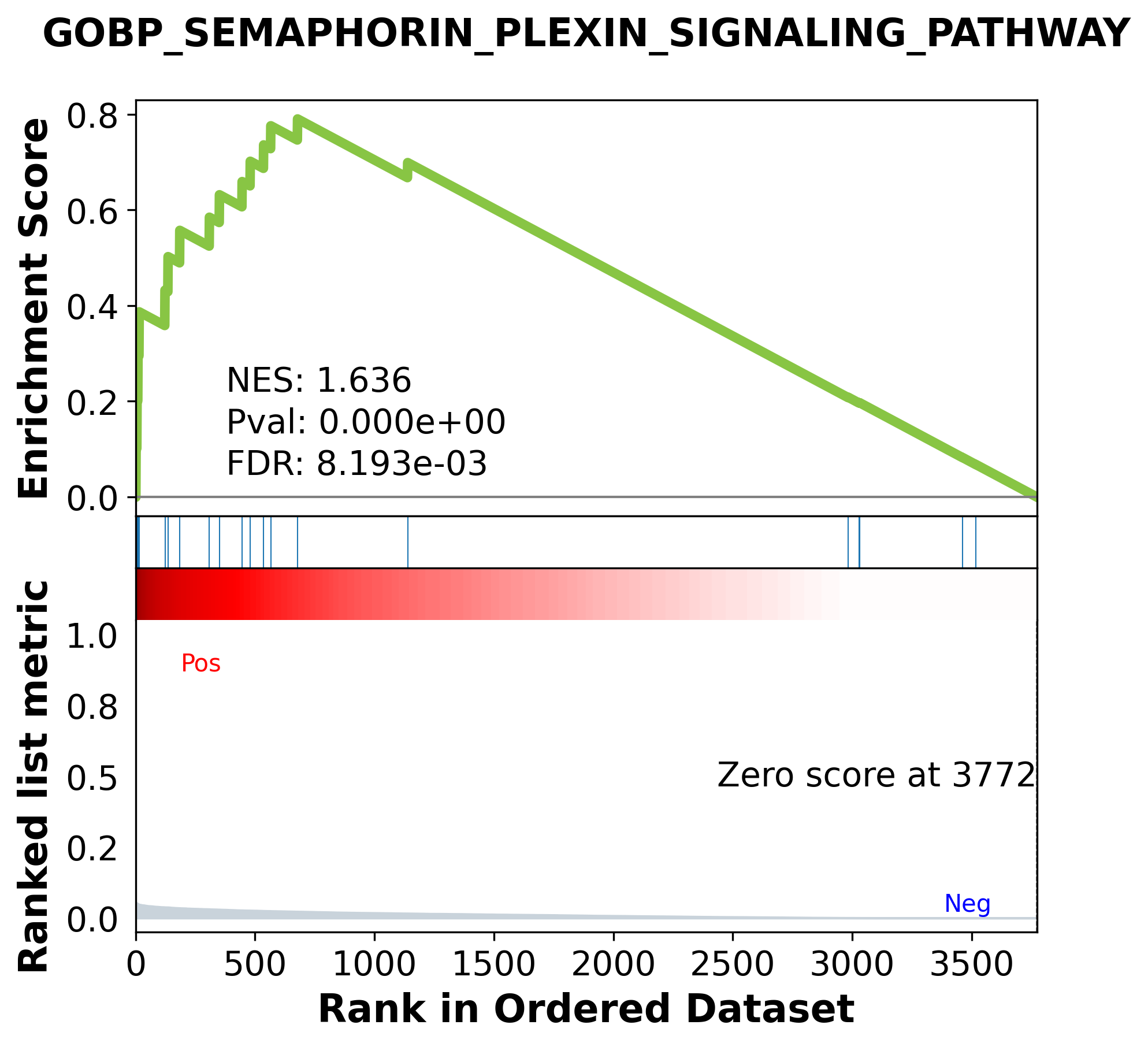

Supplement: Supplemental GSEA [file jciinsight-8-173374-s056.zip › GSEA/Factor 1/prerank/GOBP_SEMAPHORIN_PLEXIN_SIGNALING_PATHWAY.png]

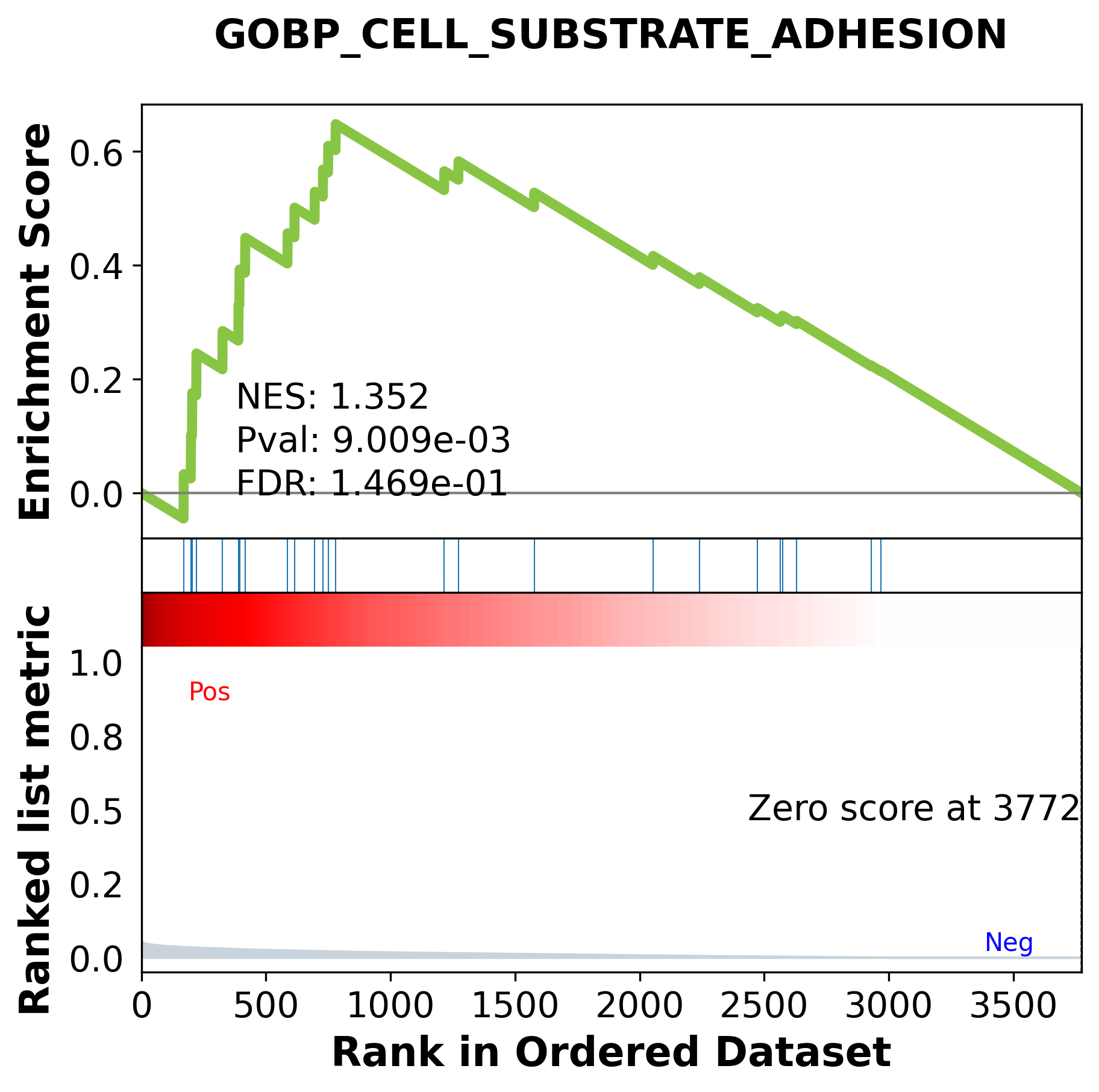

Supplement: Supplemental GSEA [file jciinsight-8-173374-s056.zip › GSEA/Factor 1/prerank/GOBP_CELL_SUBSTRATE_ADHESION.png]

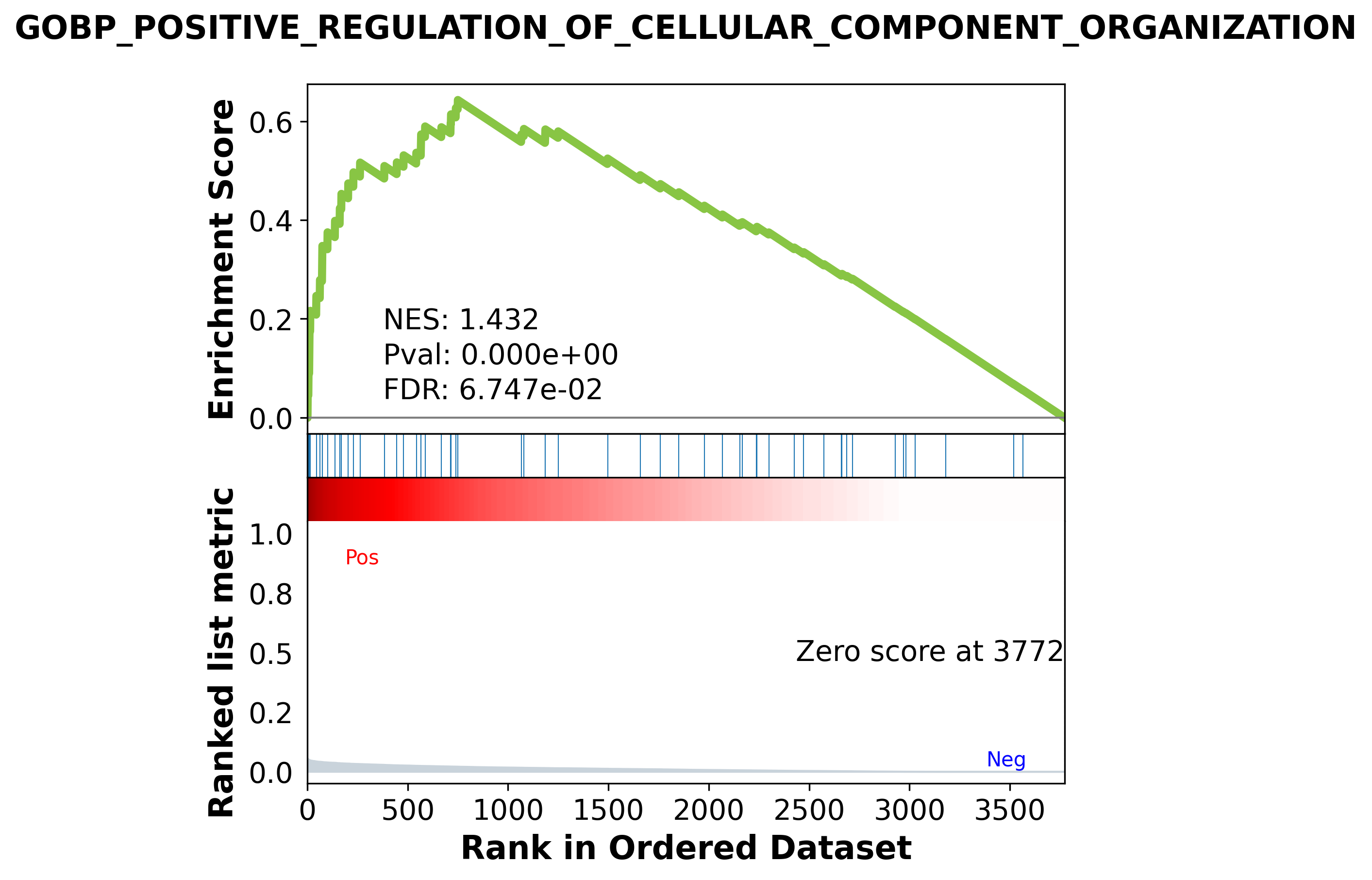

Supplement: Supplemental GSEA [file jciinsight-8-173374-s056.zip › GSEA/Factor 1/prerank/GOBP_POSITIVE_REGULATION_OF_CELLULAR_COMPONENT_ORGANIZATION.png]

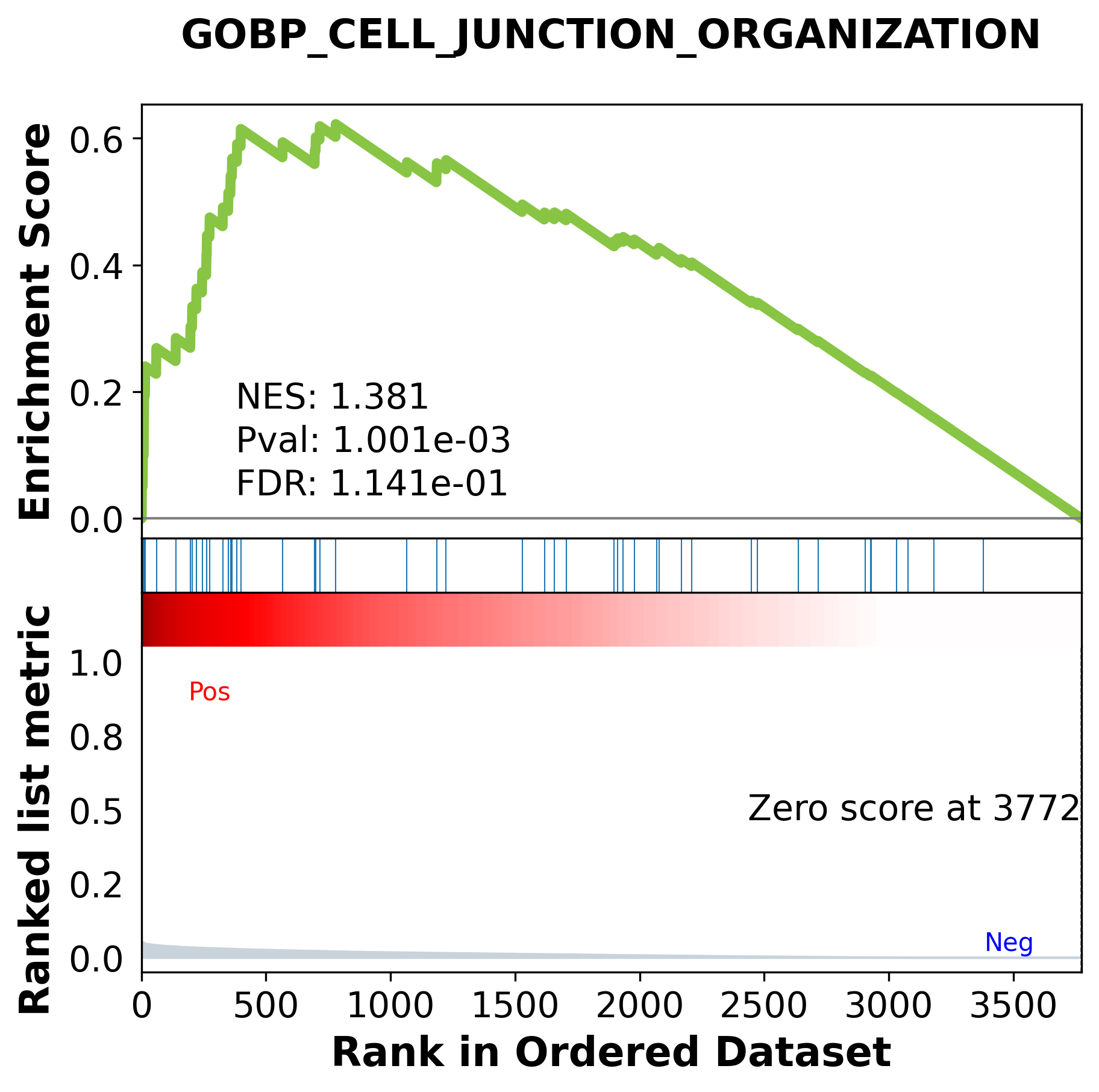

Supplement: Supplemental GSEA [file jciinsight-8-173374-s056.zip › GSEA/Factor 1/prerank/GOBP_CELL_JUNCTION_ORGANIZATION.png]

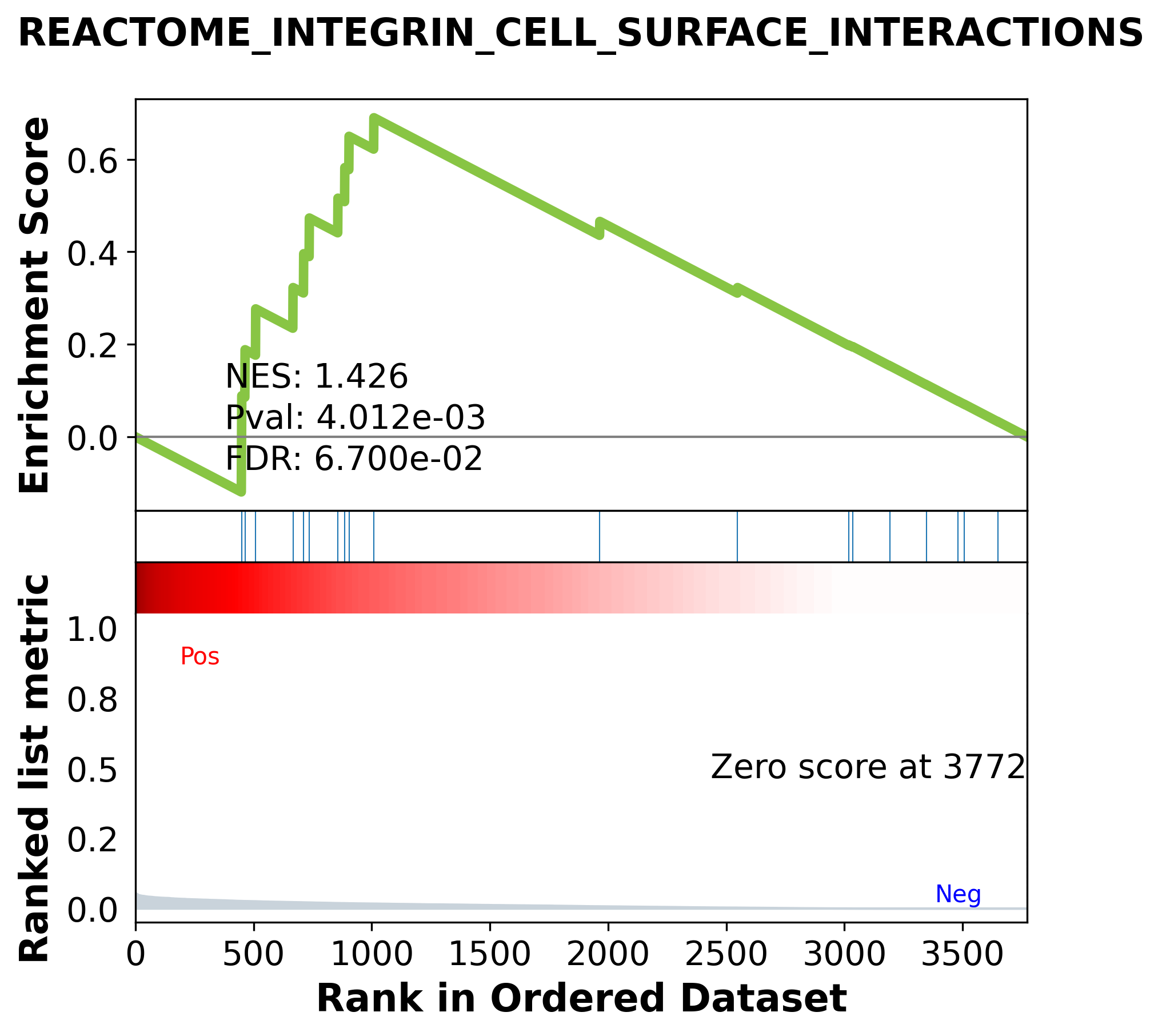

Supplement: Supplemental GSEA [file jciinsight-8-173374-s056.zip › GSEA/Factor 1/prerank/REACTOME_INTEGRIN_CELL_SURFACE_INTERACTIONS.png]

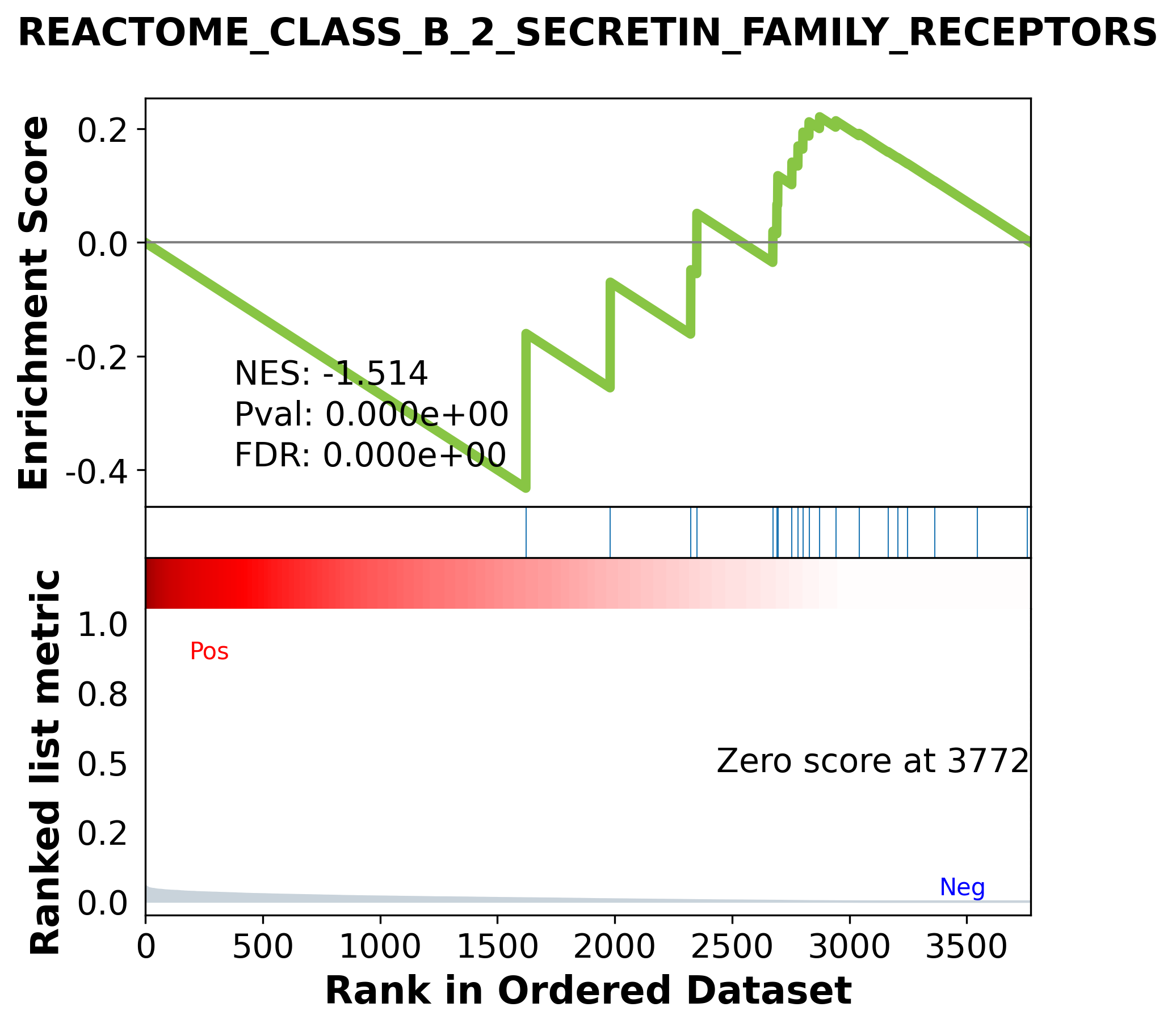

Supplement: Supplemental GSEA [file jciinsight-8-173374-s056.zip › GSEA/Factor 1/prerank/REACTOME_CLASS_B_2_SECRETIN_FAMILY_RECEPTORS.png]

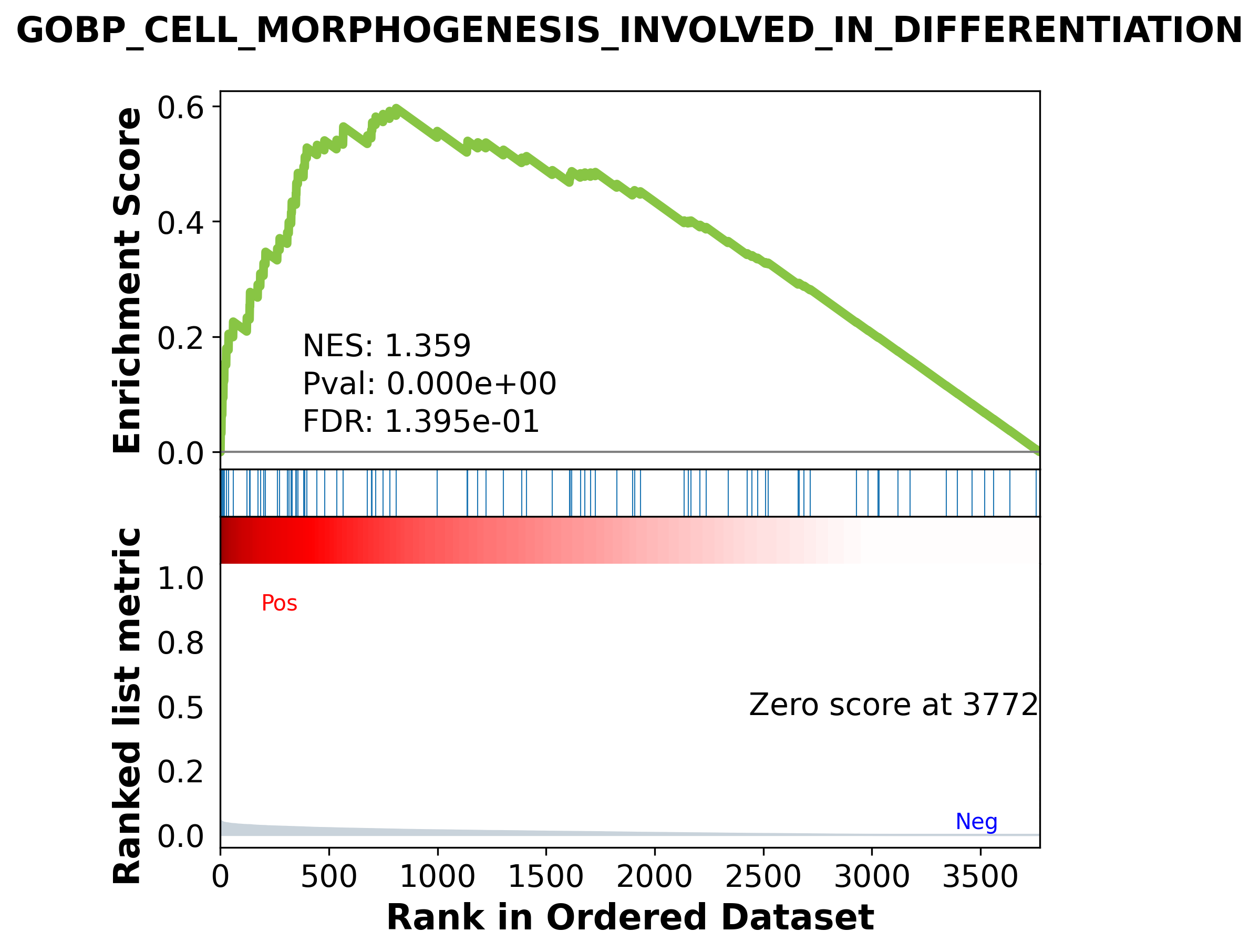

Supplement: Supplemental GSEA [file jciinsight-8-173374-s056.zip › GSEA/Factor 1/prerank/GOBP_CELL_MORPHOGENESIS_INVOLVED_IN_DIFFERENTIATION.png]

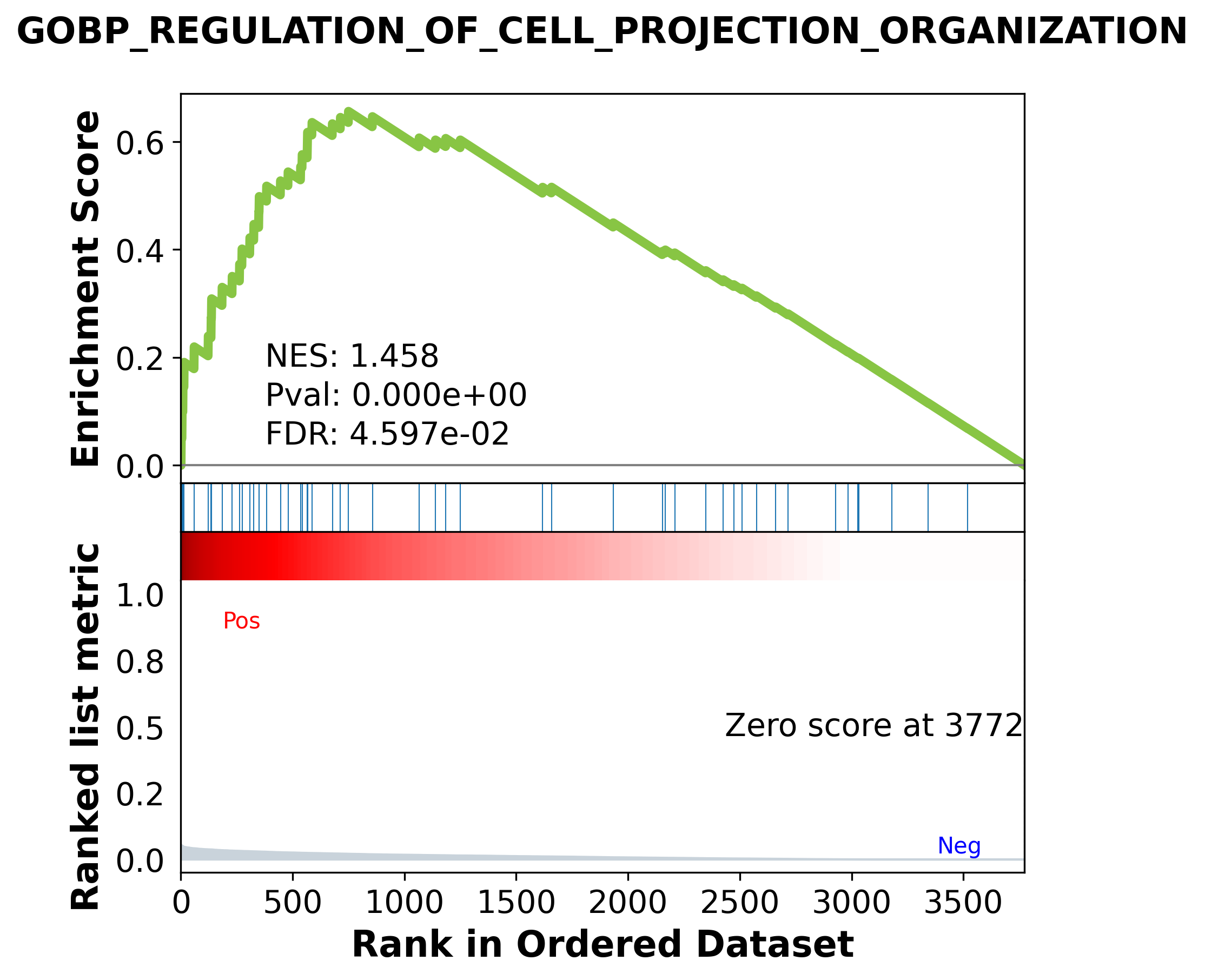

Supplement: Supplemental GSEA [file jciinsight-8-173374-s056.zip › GSEA/Factor 1/prerank/GOBP_REGULATION_OF_CELL_PROJECTION_ORGANIZATION.png]

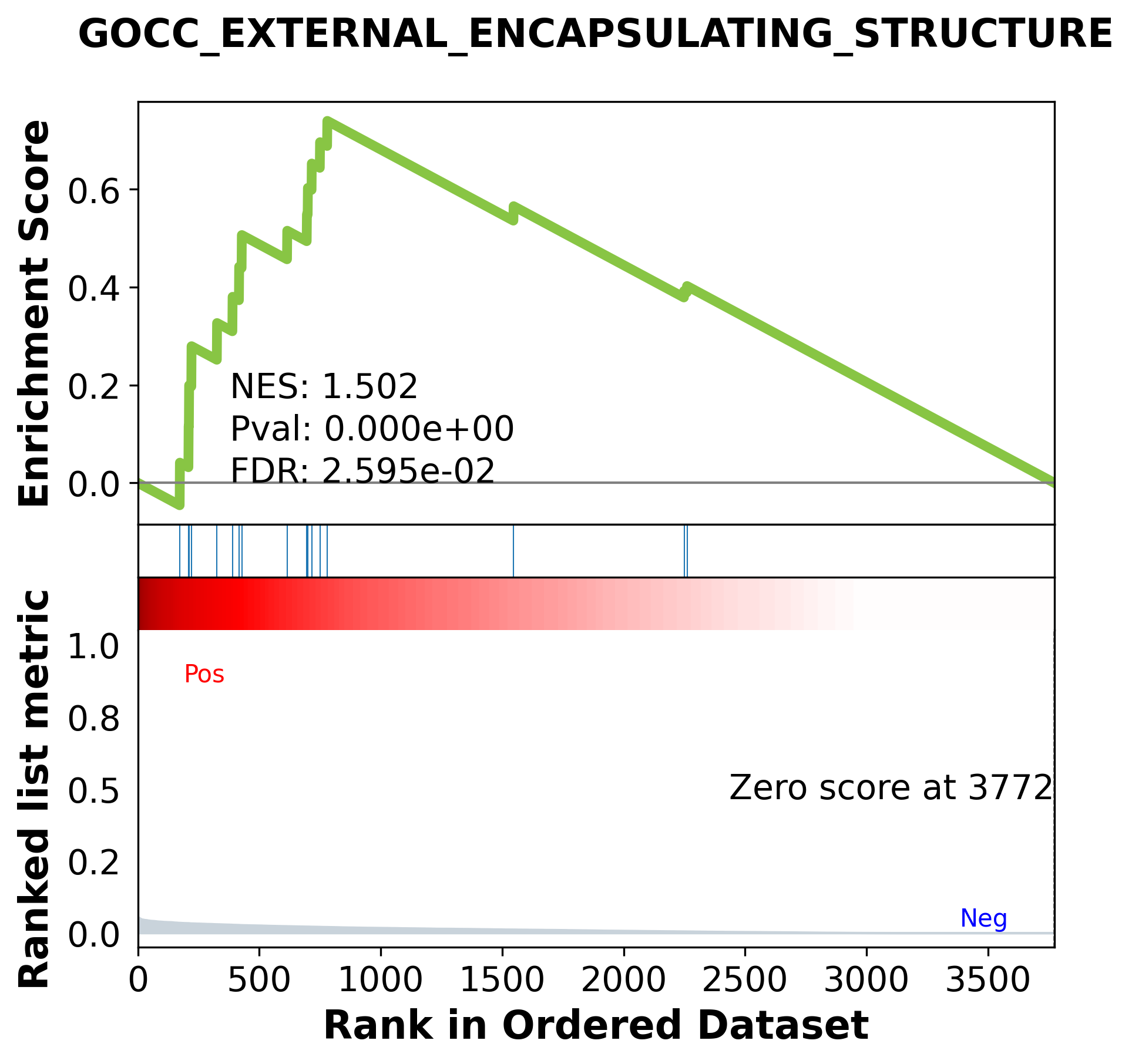

Supplement: Supplemental GSEA [file jciinsight-8-173374-s056.zip › GSEA/Factor 1/prerank/GOCC_EXTERNAL_ENCAPSULATING_STRUCTURE.png]

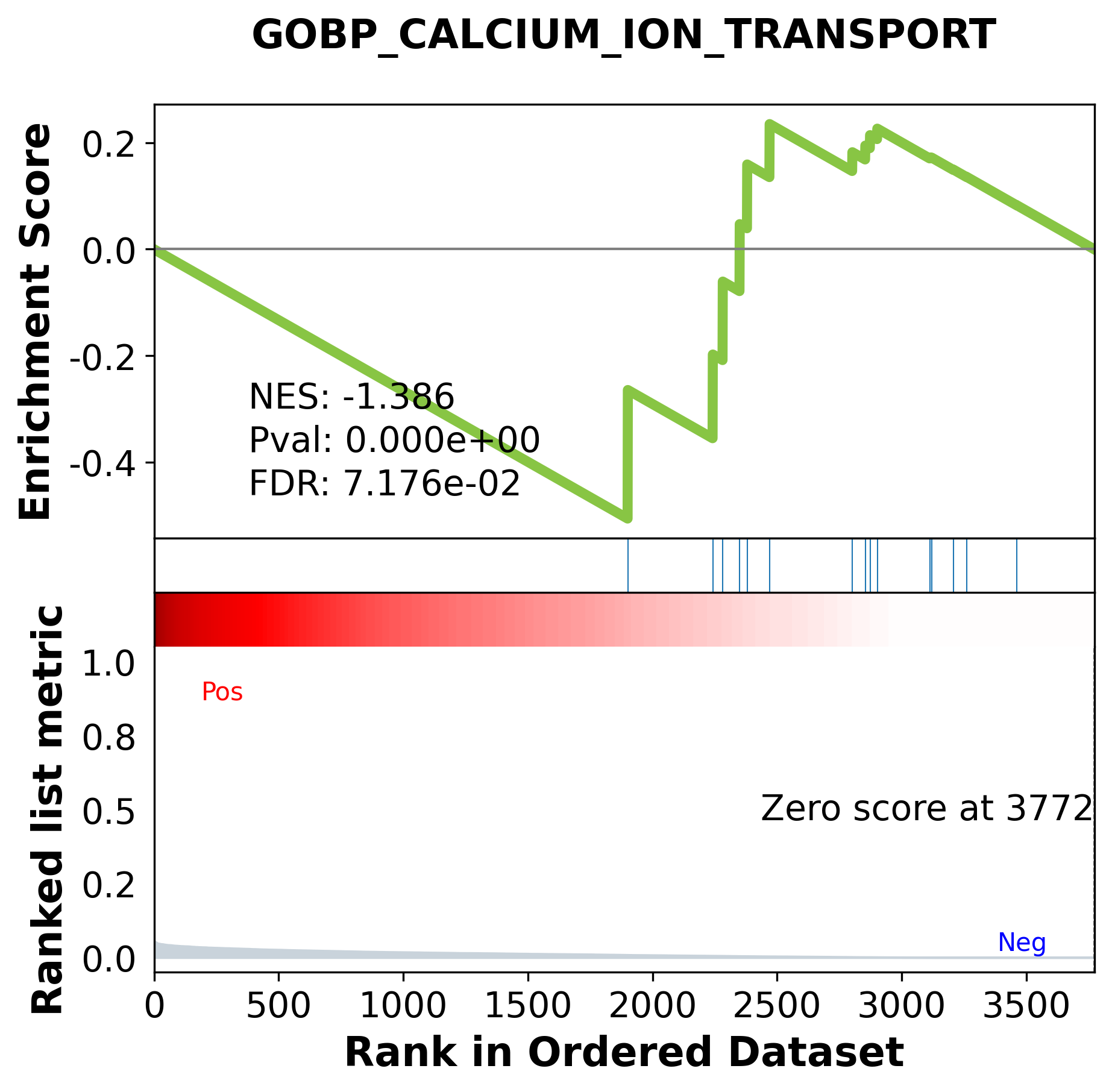

Supplement: Supplemental GSEA [file jciinsight-8-173374-s056.zip › GSEA/Factor 1/prerank/GOBP_CALCIUM_ION_TRANSPORT.png]

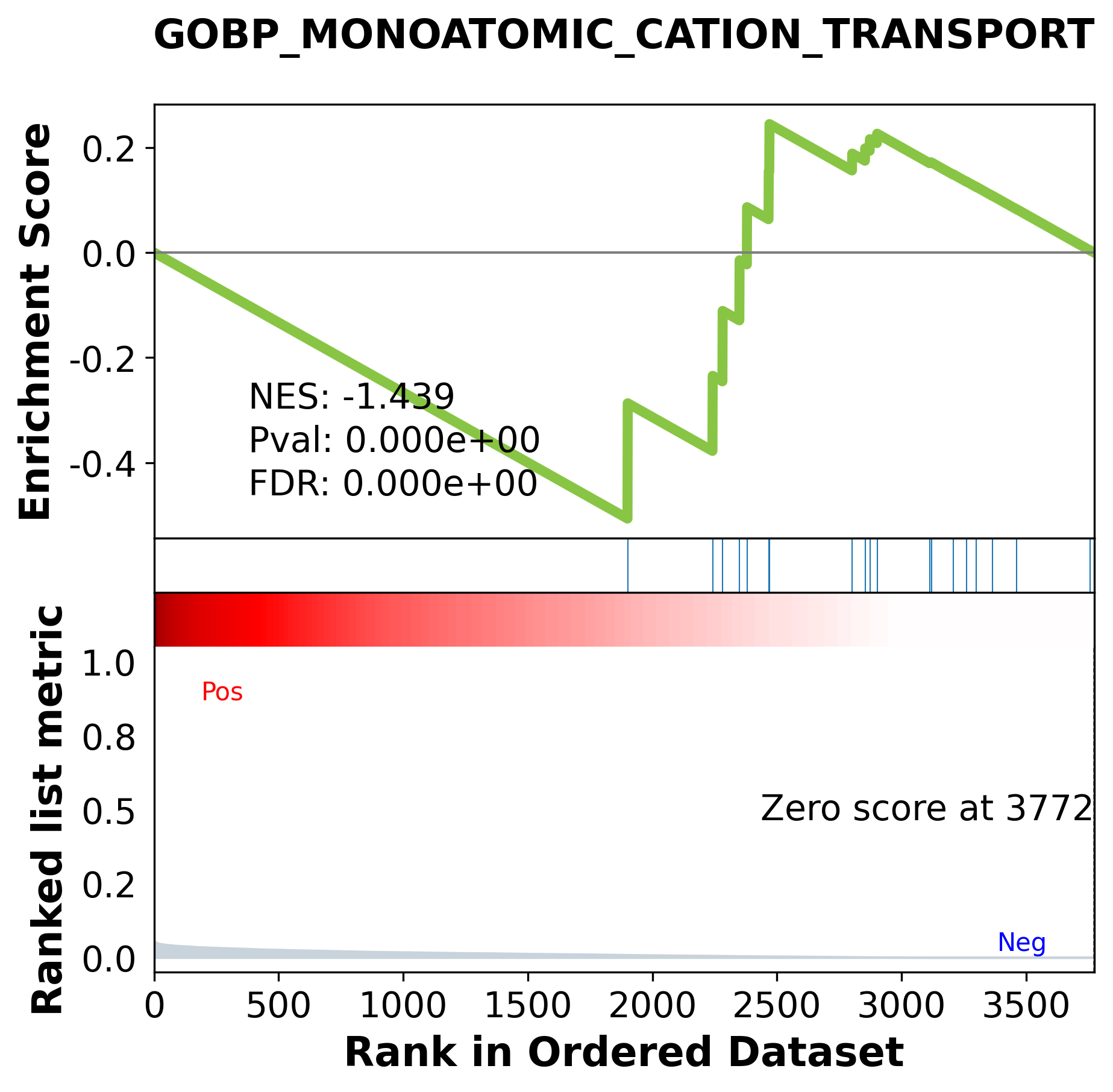

Supplement: Supplemental GSEA [file jciinsight-8-173374-s056.zip › GSEA/Factor 1/prerank/GOBP_MONOATOMIC_CATION_TRANSPORT.png]

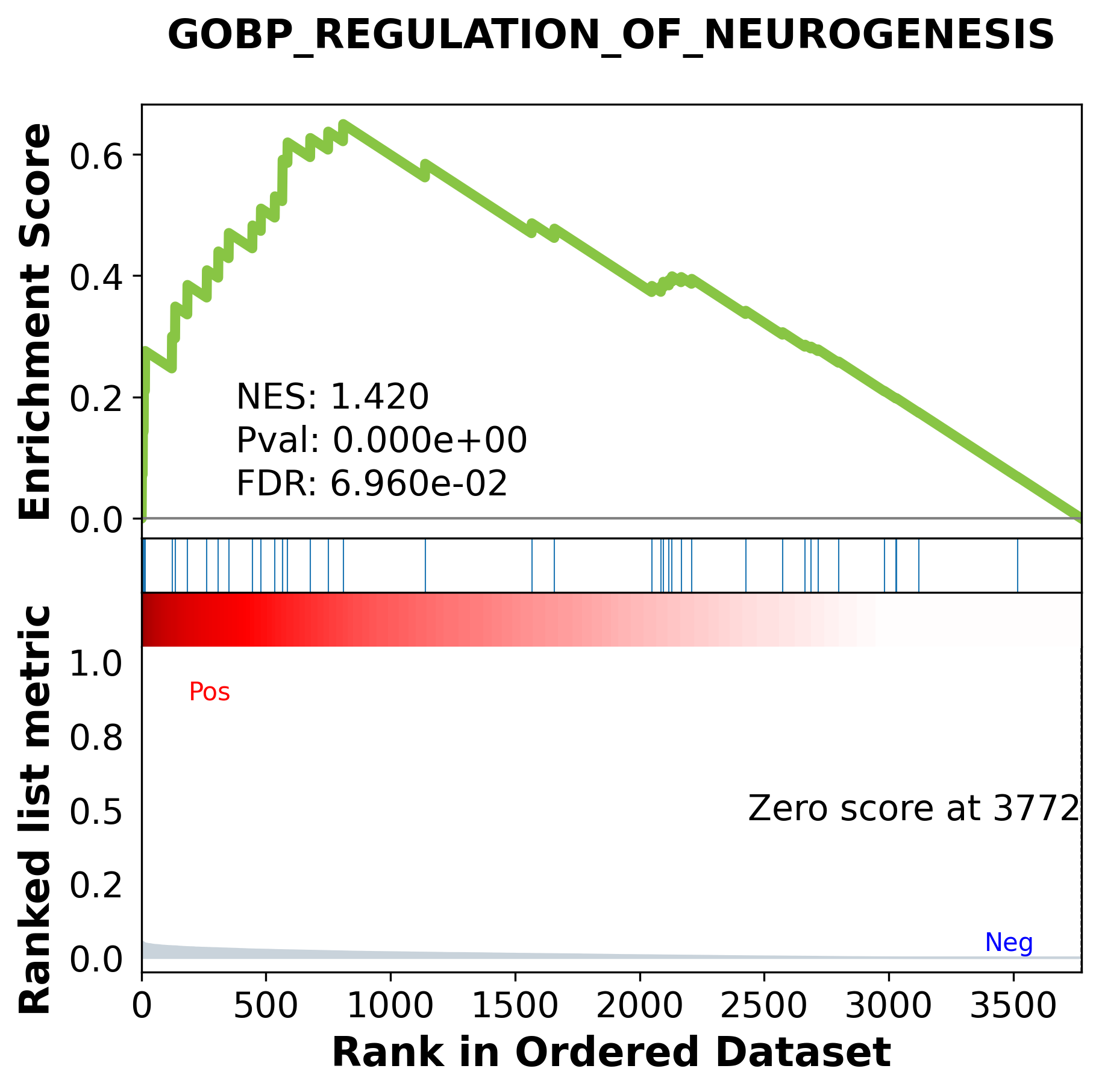

Supplement: Supplemental GSEA [file jciinsight-8-173374-s056.zip › GSEA/Factor 1/prerank/GOBP_REGULATION_OF_NEUROGENESIS.png]

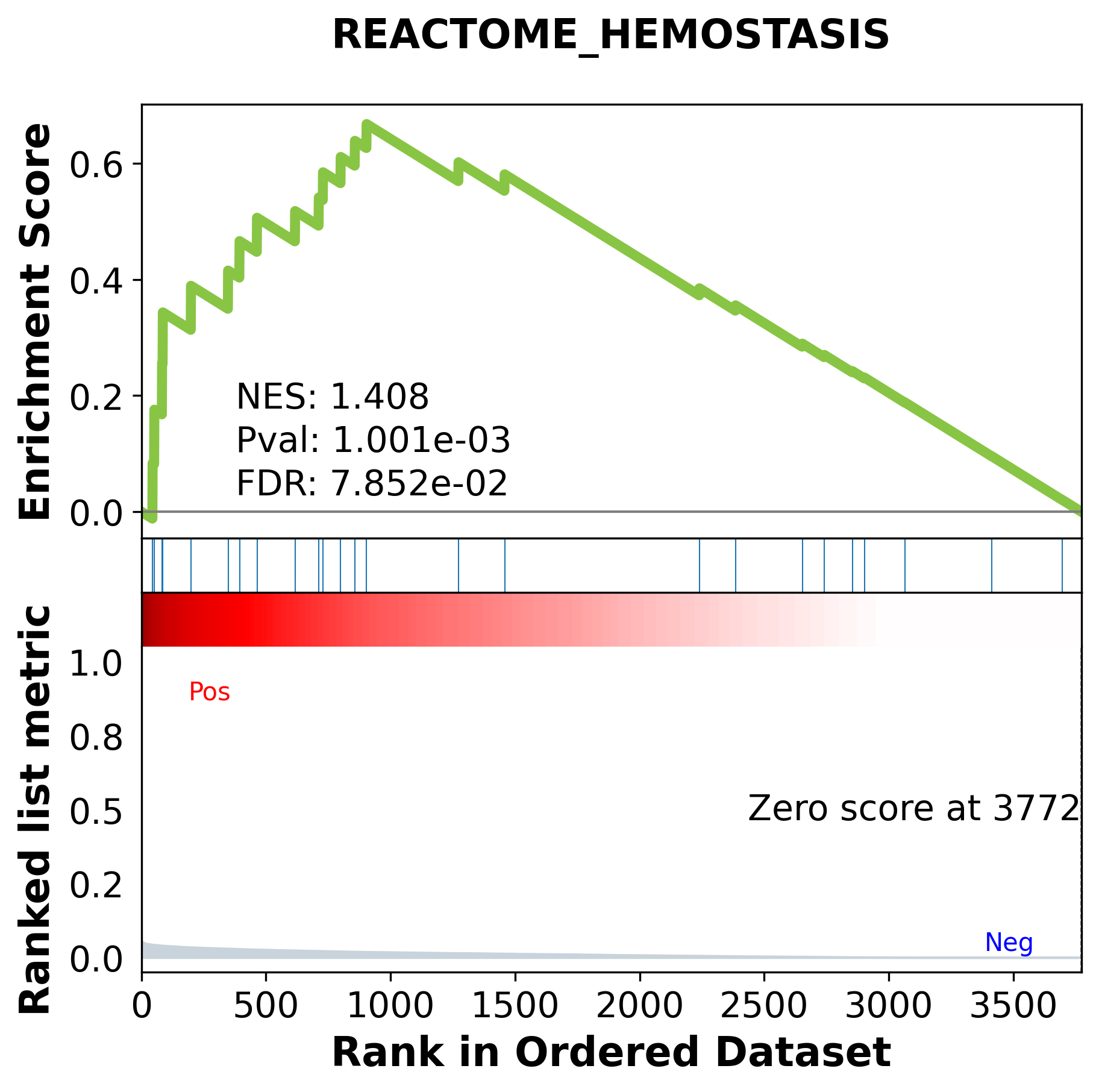

Supplement: Supplemental GSEA [file jciinsight-8-173374-s056.zip › GSEA/Factor 1/prerank/REACTOME_HEMOSTASIS.png]

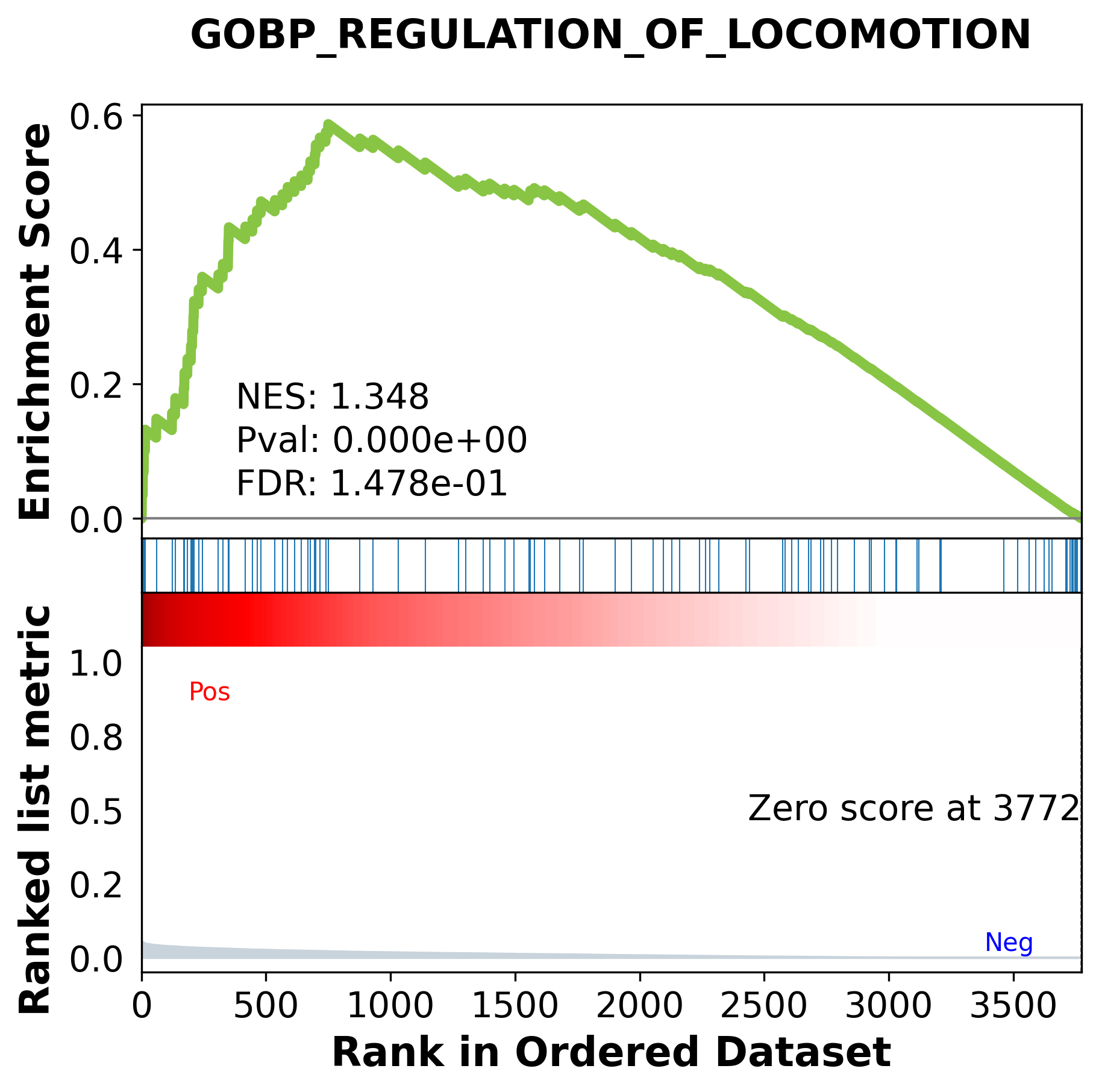

Supplement: Supplemental GSEA [file jciinsight-8-173374-s056.zip › GSEA/Factor 1/prerank/GOBP_REGULATION_OF_LOCOMOTION.png]

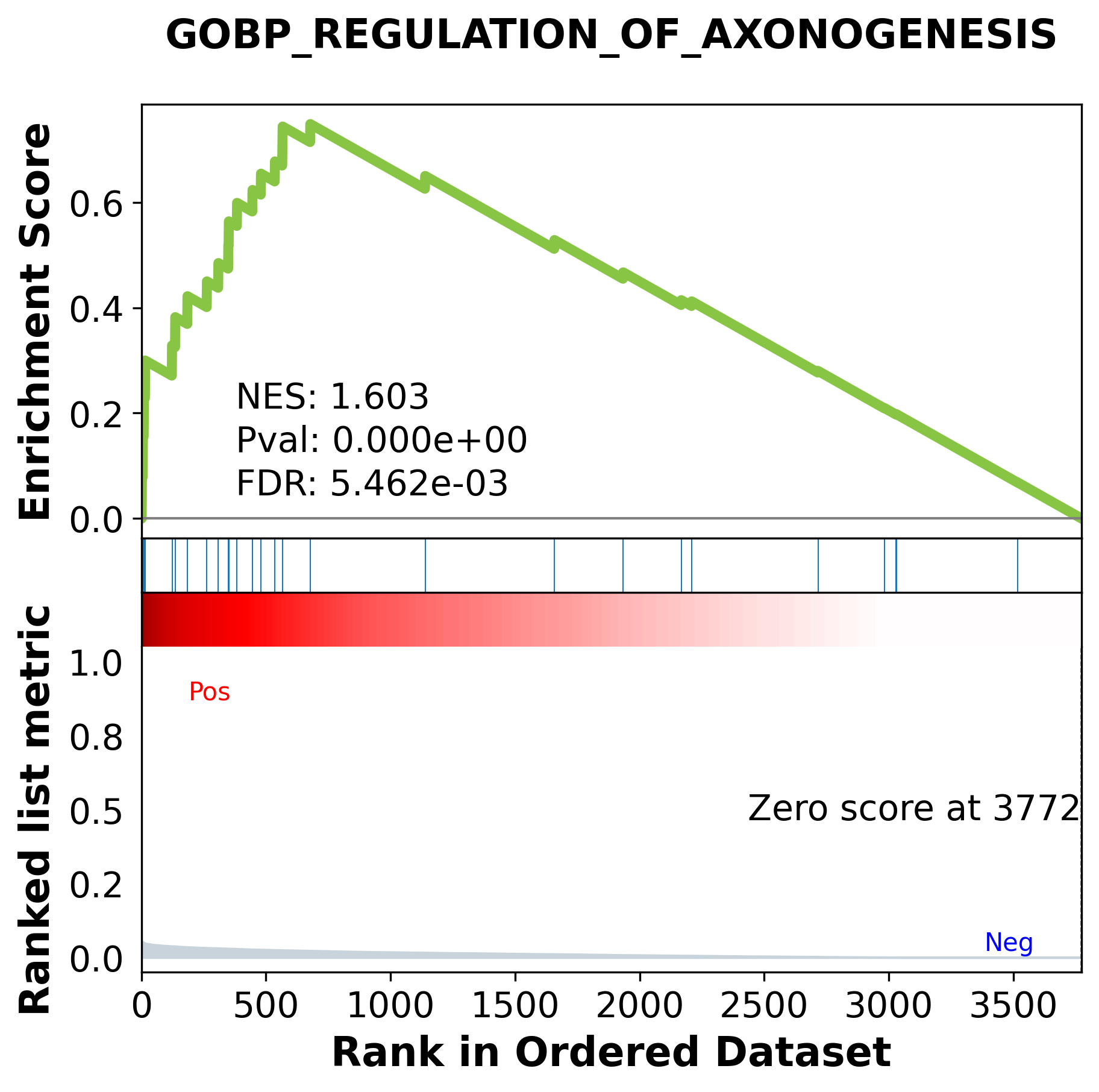

Supplement: Supplemental GSEA [file jciinsight-8-173374-s056.zip › GSEA/Factor 1/prerank/GOBP_REGULATION_OF_AXONOGENESIS.png]

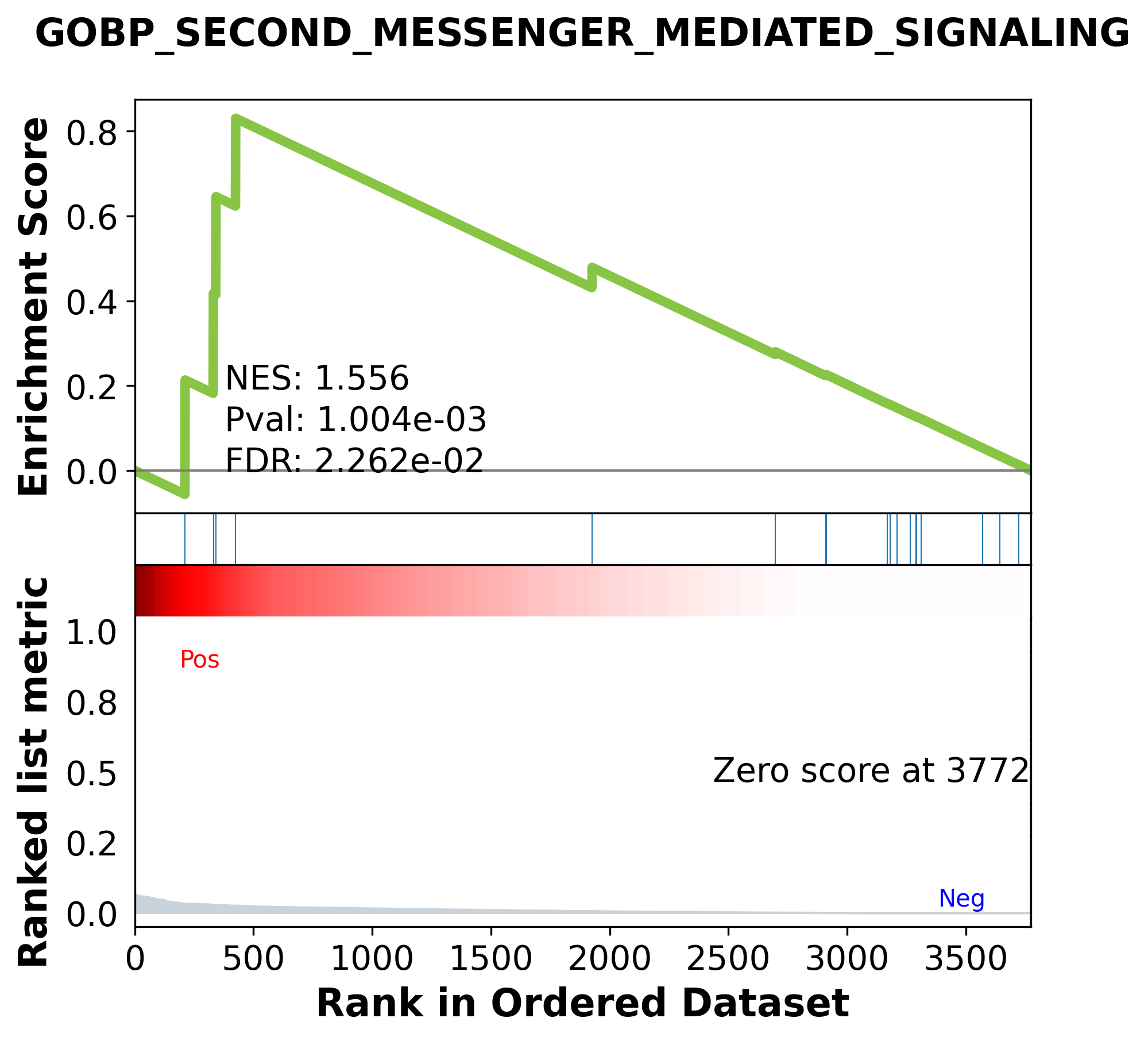

Supplement: Supplemental GSEA [file jciinsight-8-173374-s056.zip › GSEA/Factor 6/prerank/GOBP_SECOND_MESSENGER_MEDIATED_SIGNALING.png]

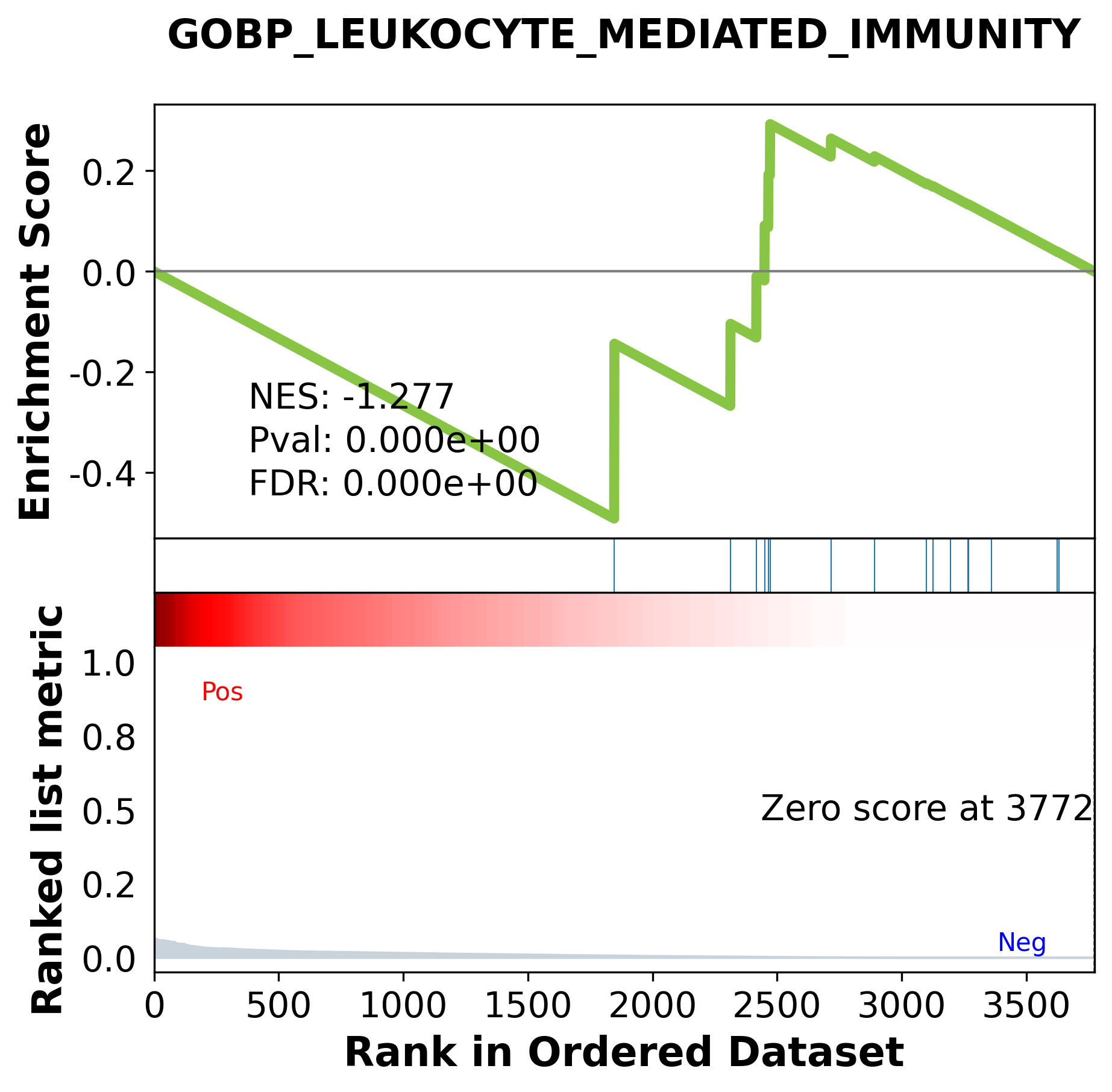

Supplement: Supplemental GSEA [file jciinsight-8-173374-s056.zip › GSEA/Factor 6/prerank/GOBP_LEUKOCYTE_MEDIATED_IMMUNITY.png]
